# Genomic Alteration in Head and Neck Squamous Cell Carcinoma (HNSCC) Cell Lines Inferred from Karyotyping, Molecular Cytogenetics, and Array Comparative Genomic Hybridization

**DOI:** 10.1371/journal.pone.0160901

**Published:** 2016-08-08

**Authors:** Worapong Singchat, Ekarat Hitakomate, Budsaba Rerkarmnuaychoke, Aorarat Suntronpong, Beiyuan Fu, Winai Bodhisuwan, Surin Peyachoknagul, Fengtang Yang, Sittichai Koontongkaew, Kornsorn Srikulnath

**Affiliations:** 1 Laboratory of Animal Cytogenetics and Comparative Genomics, Department of Genetics, Faculty of Science, Kasetsart University, 50 Ngamwongwan, Chatuchak, Bangkok, 10900, Thailand; 2 Faculty of Dentistry, Thammasart University, Pathum Thani, 12121, Thailand; 3 Department of Pathology, Faculty of Medicine, Ramathibodi Hospital, Mahidol University, Bangkok, 10400, Thailand; 4 Wellcome Sanger Institute, Wellcome Trust Genome Campus, Hinxton, Cambridge, CB10 1SA, United Kingdom; 5 Department of Statistics, Faculty of Science, Kasetsart University, 50 Ngamwongwan, Chatuchak, Bangkok, 10900, Thailand; 6 Center of Advanced Studies in Tropical Natural Resources, National Research University-Kasetsart University, Kasetsart University, Thailand (CASTNAR, NRU-KU, Thailand); University of Navarra, SPAIN

## Abstract

Genomic alteration in head and neck squamous cell carcinoma (HNSCC) was studied in two cell line pairs (HN30-HN31 and HN4-HN12) using conventional C-banding, multiplex fluorescence *in situ* hybridization (M-FISH), and array comparative genomic hybridization (array CGH). HN30 and HN4 were derived from primary lesions in the pharynx and base of tongue, respectively, and HN31 and HN12 were derived from lymph-node metastatic lesions belonging to the same patients. Gain of chromosome 1, 7, and 11 were shared in almost all cell lines. Hierarchical clustering revealed that HN31 was closely related to HN4, which shared eight chromosome alteration cases. Large C-positive heterochromatins were found in the centromeric region of chromosome 9 in HN31 and HN4, which suggests complex structural amplification of the repetitive sequence. Array CGH revealed amplification of 7p22.3p11.2, 8q11.23q12.1, and 14q32.33 in all cell lines involved with tumorigenesis and inflammation genes. The amplification of 2p21 (*SIX3*), 11p15.5 (*H19*), and 11q21q22.3 (*MAML2*, *PGR*, *TRPC6*, and *MMP* family) regions, and deletion of 9p23 (*PTPRD*) and 16q23.1 (*WWOX*) regions were identified in HN31 and HN12. Interestingly, partial loss of *PTPRD* (9p23) and *WWOX* (16q23.1) genes was identified in HN31 and HN12, and the level of gene expression tended to be the down-regulation of *PTPRD*, with no detectable expression of the *WWOX* gene. This suggests that the scarcity of *PTPRD* and *WWOX* genes might have played an important role in progression of HNSCC, and could be considered as a target for cancer therapy or a biomarker in molecular pathology.

## Introduction

Genomic reorganizations have played an important role in the process of tumor development from a single precursor cell to invasive carcinoma. The occurrence of non-homologous recombination and gene conversion result in chromosomal rearrangements (translocations, insertions, or deletions), amplifications, point mutations, and epigenetics, which often alter the function of proteins [1, 2]. A consequence of chromosome number alteration and genomic copy number variations (CNVs) is the dysregulation of proto-oncogenes or tumor suppressor gene expression, leading to numerous types of dysplasia and neoplasia [3].

Head and neck squamous cell carcinoma (HNSCC) is one of the major causes of global cancer-related mortality, estimated at between 223,000 and 300,000 deaths per year [4]. From 2002 to 2004, 1,186 head and neck cancer cases were diagnosed in Thailand, consisting of 34.6% oral cavity cases, 30.1% oropharynx cases, 16.7% hypopharynx cases, and 18.6% larynx cases [5]. Major risk factors are known to be tobacco use, alcohol consumption, betel quid chewing, and bidi smoking. Over 26,000 Thai people were diagnosed with head and neck cancers in 2010 [6]. Although numerous advances in diagnosis and treatment of oral cancer are available, mortality and morbidity rates for head and neck cancers are still high. This could reflect a high variation of genetic instability or molecular heterogeneity, and complexities of subcellular abnormalities through oral carcinogenesis. Several reports have investigated the molecular mechanisms of HNSCC development [3, 7, 8]. However, most analyzed gene expression patterns with small populations, and tumor progression stages from different patients. It is necessary to confirm the results from larger groups of patients, because HNSCCs are probably derived from different subsites within the oral cavity, known as ‘field cancerization’ [9]. Within a single tumor mass, the occurrence of a single clone that evolves into different subpopulations, such as ‘second field tumor’ (SFT), resulting from accumulation of diverse genetic variation, is designated as the clonal evolution model [10]. Two genetically heterogeneous subpopulations coexist within a single tumor mass, and the predominant population is probably replaced by other subpopulations based on the effects of environmental selection pressure and/or the stage of tumor progression from primary tumor to lymph node metastasis [11]. Alternatively, if the individual tumors show different genomic alterations within the same site, the secondary lesion might be regarded as a second primary tumor (SPT) [12]. The study of genome profiling of HNSCC is required to elucidate genetic loci relating to tumor classification and homogeneity, novel diagnosis, and therapeutic clinical management. Previous research has elucidated the genomic profile of HNSCC or cell lines using comparative genomic hybridization (CGH), and multiplex ligation-dependent probe amplification (MLPA) to determine various CNVs [13, 14]. However, these methods were not able to detect balanced chromosomal rearrangements (e.g., translocations or inversions), interchromosomal rearrangements, and low frequency mosaicism. Cytogenetic techniques such as karyotyping and multiplex fluorescence *in situ* hybridization (M-FISH) are thus needed to extensively examine genome alterations in HNSCC.

The study of tumor cell lines is a useful strategy to obtain insights into cell-specific gene regulation. Many tumor cell lines have been studied to identify regions which relate to oncogenic or tumor-suppressive genes in HNSCC [3]; however, little data is available on primary tumor and metastasis derived cell lines isolated from the same patients [15, 16]. The relevance of chromosome alteration and genomic CNVs, corresponding to the stage of phenotypic progression and invasive malignancy, has not been fully characterized. The HN cell lines are commonly used for cellular function studies. Transcriptomic and proteomic analyses in HNSCC have been obtained from these cell lines [3, 13, 15, 17]. In this study, the genome profiling of four HN cell lines (HN30, HN31, HN4, and HN12) derived from two patients was analyzed. Two cell lines were primary tumor, and the other two were lymph node metastases. We performed multiplex-fluorescence *in situ* hybridization (M-FISH) to investigate structural and numerical chromosome alterations, and analyzed cell clonality within each cell line by hierarchical clustering analysis. The heterochromatin distribution was also examined using C-banding in each cell line. High-resolution array comparative genomic hybridization (array CGH) was then conducted to identify CNVs. The mRNA expression of specific genes was identified using real time quantitative reverse transcription PCR (real time qRT-PCR), and the candidate genes and regions that related to the molecular pathology and tumor progression in HNSCC were discussed.

## Materials and Methods

### Cell lines

Head and neck squamous cell carcinoma (HNSCC) cell lines (HN30, HN31, HN4, and HN12) were provided by J. Silvio Gutkind (NIDCR, Bethesda, MD, USA). HN30 (T3N0M0) and HN31 (T3N0M0) were both derived from the same male patient, and HN4 (T4N1M0) and HN12 (T4N1M0) were derived from another male patient. HN30 and HN4 were obtained from primary lesions in the pharynx and the base of tongue, respectively. HN12 and HN31 were derived from lymph node metastases [17]. Whole genomic DNA was isolated from the four HNSCC cell lines using a salting out method as described previously [18]. Cross-contamination for each cell line was examined using STR DNA profiling analysis (Investigator® IDplex Plus, Qiagen, GmbH, Hilden, Germany) to confirm the purity of each cell line (data not shown). A human mucoepidermoid pulmonary carcinoma NCI-H292 (ATCC number CRL-1848), and a non-tumorigenic human skin keratinocyte cell line HaCaT provided by Stitya Sirisingha (Mahidol University, Thailand) were used as control groups in the expression analysis of candidate genes. This study was approved by the Institute Biosafety Committee of Thammasart University (TU-IBC, Approval No 011/2558).

### Cell culture and chromosome preparation

HN30, HN31, HN4, and HN12 were cultured in Dulbecco's modified Eagle's medium (Life Technologies-Gibco, Carlsbad, CA, USA) supplemented with 10% fetal bovine serum (Life Technologies-Gibco), 100 mg/ml penicillin—streptomycin (Life Technologies-Gibco), 100 mg/ml Amphotericin B and incubated at 37°C in a humidified atmosphere of 5% CO_2_ in air. Chromosome preparation was conducted as described previously [19]. Briefly, before harvest, HNSCC cells in the logarithmic growth phase were incubated with 120 ng/ml colcemid (Life Technologies-Gibco) for 45 min. The cells were harvested by treatment with 0.025% trypsin-EDTA, suspended in 0.075 M KCl at room temperature for 20 min, and fixed with methanol/ acetic acid (3:1) three times. The cell suspension was dropped on cleaned glass slides and air-dried. The slides were kept at -80°C until used. HaCaT and NCI cell lines were cultured in medium under conditions described previously [20, 21].

### Multiplex fluorescence *in situ* hybridization (M-FISH)

Chromosome-specific DNA libraries were generated from 5,000 copies of flow-sorted chromosomes, provided by Flow Cytometry Core Facility of the Wellcome Trust Sanger Institute, using GenomePlex Whole Genome Amplification (WGA2) kit (Sigma-Aldrich, St. Louis, MO, USA). Human 24-color painting probe was made following the pooling strategy [22]. Five human chromosome pools were labeled with ATTO 425-, ATTO 488-, Cy3-, Cy5-, and Texas Red dUTPs (Jena Bioscience, Jena, Germany), respectively, using WGA3 re-amplification kit (Sigma-Aldrich) as described previously [23]. The labeled products were pooled and sonicated to achieve a size range of 200–1,000 base pairs, optimal for use in chromosome painting. Then, the sonicated DNA samples were precipitated with ethanol together with human Cot-1 DNA (Thermo Fisher Scientific, Waltham, MA USA) and resuspended in a hybridization buffer (50% formamide, 2 × SSC [saline sodium citrate], 10% dextran sulfate, 0.5 M phosphate buffer, 1 × Denhardt’s solution [pH 7.4]). Metaphase spreads on slides were denatured by immersing in an alkaline denaturation solution (0.5 M NaOH and 1.0 M NaCl) for 50–60 s, followed by rinsing in 1M Tris-HCl (pH 7.4) solution for 3 min, 1 × PBS for 3 min, and dehydration through a 70, 90, and 100% ethanol series. The M-FISH probe was denatured at 65°C for 10 min before being applied onto the denatured slides. The hybridization area was sealed with a 22 × 22-mm coverslip and rubber cement. Hybridization was carried out in a 37°C incubator for 2 nights. The posthybridization washes included a 5-min stringent wash in 0.5 × SSC at 75°C, followed by a 5-min rinse in 2 × SSC containing 0.05% Tween20 (VWR, Radnor, PA, USA) and a 2-min rinse in 1 × PBS, both at room temperature. Finally, the slides were mounted with SlowFade Gold mounting solution containing 4′6-diamidino-2-phenylindole (Life Science, Waltham, MA, USA). Images were visualized on a Zeiss AxioImager D1 fluorescent microscope equipped with narrow band-pass filters for DAPI, DEAC, FITC, Cy3, Texas RED, and Cy5 fluorescence and an ORCA-EA CCD camera (Hamamatsu, Shizuoka, Japan). M-FISH digital images were captured using the SmartCapture software (Digital Scientific, Cambridge, UK) and processed using the SmartType Karyotyper software (Digital Scientific). Ten metaphase cells per cell line were karyotyped.

### C-banding

To examine the chromosomal distribution of constitutive heterochromatin, C-banding was performed using the standard barium hydroxide/saline/Giemsa method [24] with the following slight modification: chromosome slides were treated with 0.2 N HCl at room temperature for 60 min, and then with 5% Ba(OH)_2_ at 50°C for 1 min, followed by 2× SSC at 65°C for 60 min.

### Hierarchical clustering

A cluster analysis was performed to assess the chromosome alterations among the four cell lines by considering the type of chromosome aberrations within metaphases. Each aberration that was recorded in over five of the ten metaphase cells was computed as present or absent within the karyotype of different metaphases. The frequency (%) of each chromosome alteration was then compared among the four cell lines by Cluster 3.0 software [25] using the commonly used average linkage method and visualized with Java TreeView 1.1.6 [26].

### Array comparative genomic hybridization (array CGH)

Whole genomic DNA was used as a template for array CGH analysis with 60-mer oligonucleotide CGH arrays (Agilent Technologies, Santa Clara, CA, USA). Briefly, 20 μg of genomic DNA in each cell line and reference genomic DNA [Hg19; Genome Reference Consortium Human Reference (GRCh37), http://www.ncbi.nlm.nih.gov/assembly/GCF_000001405.13/] were digested using *Alu*I and *Rsa*I (Agilent Technologies). The digested tumor and reference DNAs were labeled with cyanine 3-dUTP (Cy3-dUTP) and cyanine 5-dUTP (Cy5-dUTP), respectively, using SureTag DNA Labeling Kit (Agilent Technologies) following the manufacturer’s instruction. Equal amounts of tumor and reference DNAs were subsequently pooled and mixed with human Cot-1 DNA (1.0 mg/ml), dissolved in Hybridization Master Mix, 10× a CGH Blocking Agent and 2× HI-RPM Hybridization Buffer (Agilent Technologies), denatured and hybridized to the CGH array at 65°C for 24 h. Glass slides were washed and then scanned in accordance with the manufacturer’s instructions. Microarray images were analyzed using Agilent C microarray scanner (Agilent Technologies) with linear normalization according to manufacturer's instructions, and the data were imported into Default Analysis Method–CGH v2 (Agilent Technologies). After normalization of the raw data, the log2 ratio of Cy3 (tumor) to Cy5 (reference) was calculated. Aberrant regions were determined using the Aberration Detection Method—2 (ADM-2) algorithm at a threshold of 6.0. To detect amplification and deletion, we set the values of parameters for the aberration filters as: minimum number of probes for amplification ≥ 3, nesting level ≤ 100, minimum average, absolute log ratio for amplification ≥ 0.25, minimum size (Kb) of region for amplification ≥ 0.0, minimum size (Kb) of region for deletion ≥ 0.0, minimum number of probes for deletion ≥ 3, and minimum average, absolute log ratio for deletion ≥ 0.25. Differences at *P* ≤ 0.05 were considered to be statistically significant.

### RNA isolation and expression analysis of candidate genes

The levels of four gene [*SIX3* (2p21), *H19* (11p15.5), *PTPRD* (9p23), and *WWOX* (16q23.1)] expressions were detected from four HNSCC cell lines, and control group: HaCaT and NCI. All cell lines were lysed with TRIzol Reagent (Life Technologies-Invitrogen, Carlsbad, CA, USA), and total RNA was isolated following the manufacturer's instructions. cDNA fragments were synthesized using iScript™ cDNA Synthesis Kit (Bio-Rad, Hercules, California USA), and used as real time PCR templates to amplify the four gene fragments. The mRNA expression of these four genes was measured by Bio-Rad iCycler Thermal Cycler with iQ5 Multicolor Real Time PCR Detection System (Bio-Rad) as signal reporter. Fifty nanograms of cDNA amplification was carried out in 10 μl of Master Mix that contained 2X iTaq^TM^ Universal SYBR^®^ Green supermix (Bio-Rad), and 10.0 μM gene-specific primers (Table 1), designed from the NCBI reference sequence of *SIX3* (LC106310), *H19* (LC106311), *PTPRD* (LC106313), and *WWOX* (LC106312) genes. PCR conditions were as follows: an initial denaturation at 95°C for 30 s, followed by 50 cycles that each involved incubation at 95°C for 15 s, 58°C for 30 s and 95°C for 1 min, and 55°C for 1 min. Dissociation melting curves, created by running a heat dissociation protocol after the PCR (81 cycles of 55.0°C– 95.0°C for 10 s, increase set point temperature by 0.5°C), confirmed the specific amplification of cDNA target and the absence of nonspecific products. The *GAPDH* gene was also used as the internal reference to normalize the expression levels between samples, using GAPDH_01_F (5′-GATAACGGATTTGGTCGTATTG-3′) and GAPDH_01_R (5′-CATGGGTGGAATCATATTGGAA-3′) primers [27]. Quantitative real time PCR reactions of all cell lines were performed in biological triplicates. Threshold cycle (Ct) values were used to calculate ∆Ct values from the products of *GAPDH* and *SIX3*, *H19*, *PTPRD*, and *WWOX* genes for all cell lines. The relative expression levels of different cell lines were calculated using the 2^−∆∆Ct^ method [28]. Statistical differences of *SIX3*, *H19*, *PTPRD*, and *WWOX* genes between cell lines were detected by one-way analysis of variance (ANOVA) with Tukey's multiple comparison test, using GraphPad Prism V5 (GraphPad Software, La Jolla, CA, USA), and the level of statistical significance was tested and represented as * for *P* ≤ 0.05, ** for *P* ≤ 0.01, and *** for *P* ≤ 0.001. Estimated values were expressed as mean ± standard deviation. The PCR products of cDNA fragments from *SIX3*, *H19*, *PTPRD*, and *WWOX* genes were also cloned using pGEM-T Easy Vector System I (Promega, Madison, WI, USA), and the nucleotide sequences of the DNA fragments were determined using 1^st^ Base DNA sequencing service (First Base Laboratories Sdn Bhd, Seri Kembangan, Selangor, Malaysia). Nucleotide sequences of three to seven DNA clones for each gene of each cell line, and their consensus sequences were searched for homologies with the nucleotide sequences in the National Center for Biotechnology Information (NCBI) database to confirm DNA fragments of the target gene, using the BLASTx and BLASTn programs (http://blast.ncbi.nlm.nih.gov/Blast.cgi). They were then deposited in the DNA Data Bank of Japan (http://www.ddbj.nig.ac.jp/index-e.html) (Table 1).

**Table 1:** Primer sequences for real time quantitative reverse transcription PCR to evaluate the expression level of *SIX3*, *H19*, *PTPRD*, and *WWOX* genes.

target gene	primer name	primer sequence (5'-3')	size of PCR product (bp)	accession number
*SIX3*	SIX3(ex)f	CCC ACA CAA GTA GGC AAC TG	192	LC106310
	SIX3(ex)r	TTA CCG AGA GGA TGG AGG TG		
*H19*	H19(ex)f	CTC TCA GGA GGG AGG ATG GT	195	LC106311
	H19(ex)r	CAT GTC CTG CTT GTC ACG TC		
*PTPRD*	PTPRD(ex)f	AAG GCC TGG GTG CTT CTA CT	160	LC106313
	PTPRD(ex)r	GTC ATC TTC CCC ATC CAC TG		
*WWOX*	WWOX(ex)f	GGG CAC TTC TAC CTT GTC CA	160	LC106312
* *	WWOX(ex)r	ATG TTG CAG AGC TTG GAC CT		

## Results

### Chromosome alteration profile and cluster analysis

**Fig 1:**
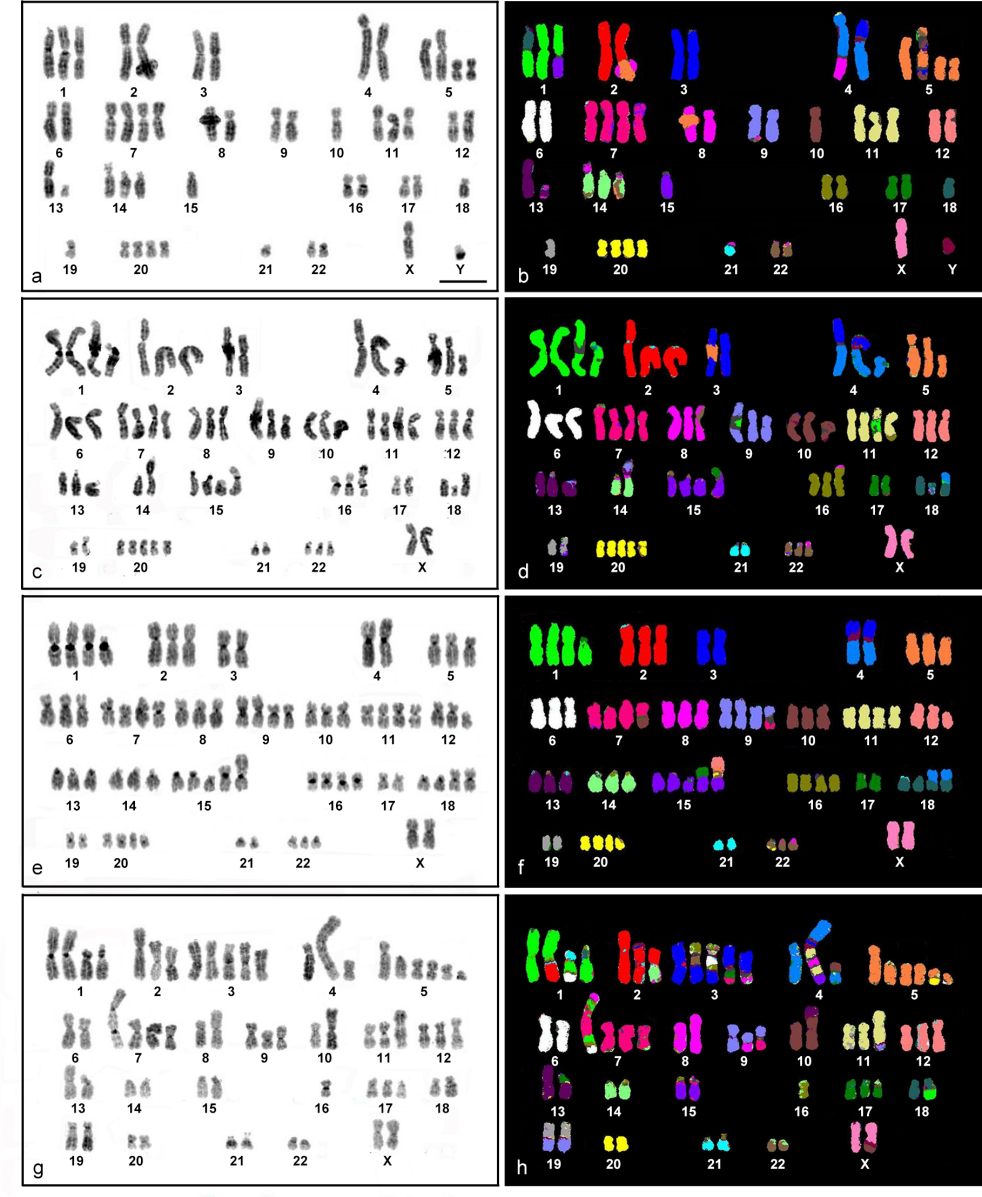
DAPI stained- and M-FISH (multiplex fluorescence *in situ* hybridization) karyotypes of four head and neck squamous cell carcinoma cell lines. DAPI stained and M-FISH karyotype of a representative metaphase indicates for HN30 (a and b), HN31(c and d), HN4 (e and f), and HN12 (g and h). Scale bars represent 10 μm.

At least 50 metaphase spreads/cell line were observed to examine the ploidy level, and 10 metaphase spreads/cell line determined chromosome alteration in both structure and number. Chromosome analysis of HN30 cell line revealed 56 different chromosome alterations (25 numerical and 31 structural). Chromosome number was 48–52 (Table 2). Chromosome gains were frequently found in the cell line, particularly chromosomes 1, 11, and 14 as trisomy, and chromosomes 5, 7, and 20 as tetrasomy (Table 2). Structural aberrations (translocations and insertions) were found in almost all chromosomes except for X, 6, 11, and 12 (Fig 1A and 1B). A cluster analysis indicated four groups (S1 Fig), all of which represented the gain of chromosome 1, 5, 7, and 20, and loss of chromosome 18, with structural aberration of chromosome 1 as der(1)t(1;15) and dic(1;18). The first group (HN30_4) showed +9, +22, ins(13;20), der(14)t(14;16)t(14;15), and der(22)t(22;15). The second group (HN30_3) showed +2, -12, dic(5;15) and ins(9;13), and the third group (HN30_2) presented -X, -13 and der(16)t(16;13). The remaining seven metaphase cells were included in the last group.

**Table 2:** Chromosome number and aneuploidy for each head and neck squamous cell carcinoma (HNSCC) cell line. Numbers in parenthesis indicate the chromosome numbers in each cell line. Numbers in square brackets indicate the quantity of metaphase cells.

chromosome	Cell line			
	HN30 (48–52)	HN31 (66–73)	HN4 (67–72)	HN12 (57–69)
1	trisomy [10]	tetrasomy [9]	tetrasomy [10]	tetrasomy [3], pentasomy [6]
2		trisomy [8]	trisomy [9]	trisomy [8]
3				pentasomy [9]
4		trisomy [10]		
5	tetrasomy [8]	trisomy [8]	trisomy [7]	hexasomy [6]
6		trisomy [9]	trisomy [10]	trisomy [7]
7	tetrasomy [7]	tetrasomy [9]	tetrasomy [10]	trisomy [4], tetrasomy [4], pentasomy [2]
8		trisomy [9]	trisomy [9]	trisomy [8]
9		trisomy [10]	tetrasomy [9]	monosomy [6]
10	monosomy [9]	trisomy [10]	trisomy [10]	
11	trisomy [9]	tetrasomy [9]	tetrasomy [10]	trisomy [6], tetrasomy [2]
12		trisomy [9]	trisomy [9]	trisomy [8]
13		trisomy [8]	trisomy [10]	
14	trisomy [8]	trisomy [7]	trisomy [7]	
15	monosomy [7]	tetrasomy [9]	pentasomy [7]	
16		trisomy [7]	trisomy [6]	
17				trisomy [10]
18	monosomy [9]	trisomy [9]	tetrasomy [8]	
19				
20	tetrasomy [9]	pentasomy [8]	tetrasomy [9]	
21				monosomy [8]
22		trisomy [7]	trisomy [8]	

For HN31, 45 different chromosome alterations (26 numerical and 19 structural) were found in all metaphase cells (Fig 1C and 1D). Chromosome number was nearly triploid (66–73) (Table 2). Chromosome gains were more often found than chromosome losses as tetrasomy in 1, 7, 11, and 15 (Table 2). Structural aberrations were found in all chromosomes except for X, 5, 6, 7, 10, 12, and 20. Eight aberration types: +1, +15, +20, -3, -17, -21, der(4)t(3;Y)t(4;Y), and der(18)t(18;4) were found in the cell lines, and a cluster analysis indicated the presence of three chromosome aberration groups (S2 Fig). Eight metaphase cells were clustered together within the second group. The first cluster (HN31_5) represented -2, -5, -6, and -18, and the third group (HN31_7) indicated +X, +8, -6, der(1)t(11;17)t(1;17), der(8)t(8;16), ins(9;Y), and der(14)t(14;8).

For HN4, 48 different chromosome alterations (22 numerical and 26 structural) were found in 10 metaphases (Fig 1E and 1F). Chromosome number was nearly triploid (67–72) (Table 2). Numerical aberrations were observed in almost all chromosomes, most of which were tetrasomic. Within 10 metaphase cells, chromosomes 1, 7, 9, 11, 18, and 20 were tetrasomic (Table 2). Structural aberrations were found in all chromosomes except for chromosome X, and 6. Ten types, +1, +7, +11,+20, -3, -4, -17, -19, der(4)t(3;Y)t(4;Y), and der(18)t(18;4) were found in all metaphases, and the types: +9, +15, +18, -Y, -21, der(1)t(1;17), der(15)t(12;20)t(15;20), der(15)t(15;19), and der(15)t(15;17) were almost homogenously distributed among the 10 metaphases. Analysis of the frequency of chromosome alteration as shown by hierarchical clustering indicated four groups (S3 Fig). Seven metaphase cells were clustered together within the second cluster. The first cluster represented chromosome alterations: der(4)t(Y;3)t(4;Y), +2, der(2)t(2;9), der(16)t(16;9), der(7)t(7;5), and der(13)t(13;21) as found in the metaphase HN4_1. Group three (HN4_10) showed +8, der(3)t(3;5), dic(2;17), der(22)t(22;4), and -22, and group four (HN4_8) showed der(14)t(14;5), der(7)t(7;Y), der(15)t(12;21)t(21;22)t(15;22), der(16)t(16;21), and der(20)t(20;4).

For HN12, 82 different chromosomal alterations (24 numerical and 58 structural chromosomal aberrations) were found (Fig 1G and 1H), and chromosome number was nearly triploid (59–67) (Table 2). Chromosome 1 was tetrasomic or pentasomic, while chromosome 3 and 5 were often pentasomic and hexasomic, respectively (Table 2). Structural aberrations were found in all chromosomes. The presence of +5, -10, der(3)t(17;7)t(3;17), +3, -Y, -22, -21, der(19)t(19;9), -19, der(18)t(18;1), -18, -15, and -13 were found in all metaphases. Clustering by the frequency of the chromosomal aberration produced four groups (S4 Fig). Six metaphase cells were clustered together within the second cluster. The first group (HN12_2) represented der(3)t(17;15)t(3;15), ins(12;6), der(8)t(8;12), and der(7)t(1;2)t(7;1)ins(15;1)t(7;15). Group three (HN12_6) showed der(5)t(5;2), der(3)t(3;22), dic(1;7)ins(1;7)t(1;8), der(2)t(2;3), der(14)t(14;1)t(12;2)t(14;12), (+11), der(6)t(6;21), der(6)t(6;16) and der(6)t(6;4), and group four (HN12_9 and HN12_10) showed der(4)t(11;15)ins(11;8)t(11;Y)t(4;Y), der(4)t(4;10), der(3)t(3;8), der(3)t(16;6)t(3;6), der(X)t(10;Y)t(X;Y), der(1)t(1;18), der(10)t(10;13), der(9)t(9;8), der(9)t(9;7), -8, dic(1;7)ins(1;7)t(1;6)t(1;8), and -6.

**Fig 2:**
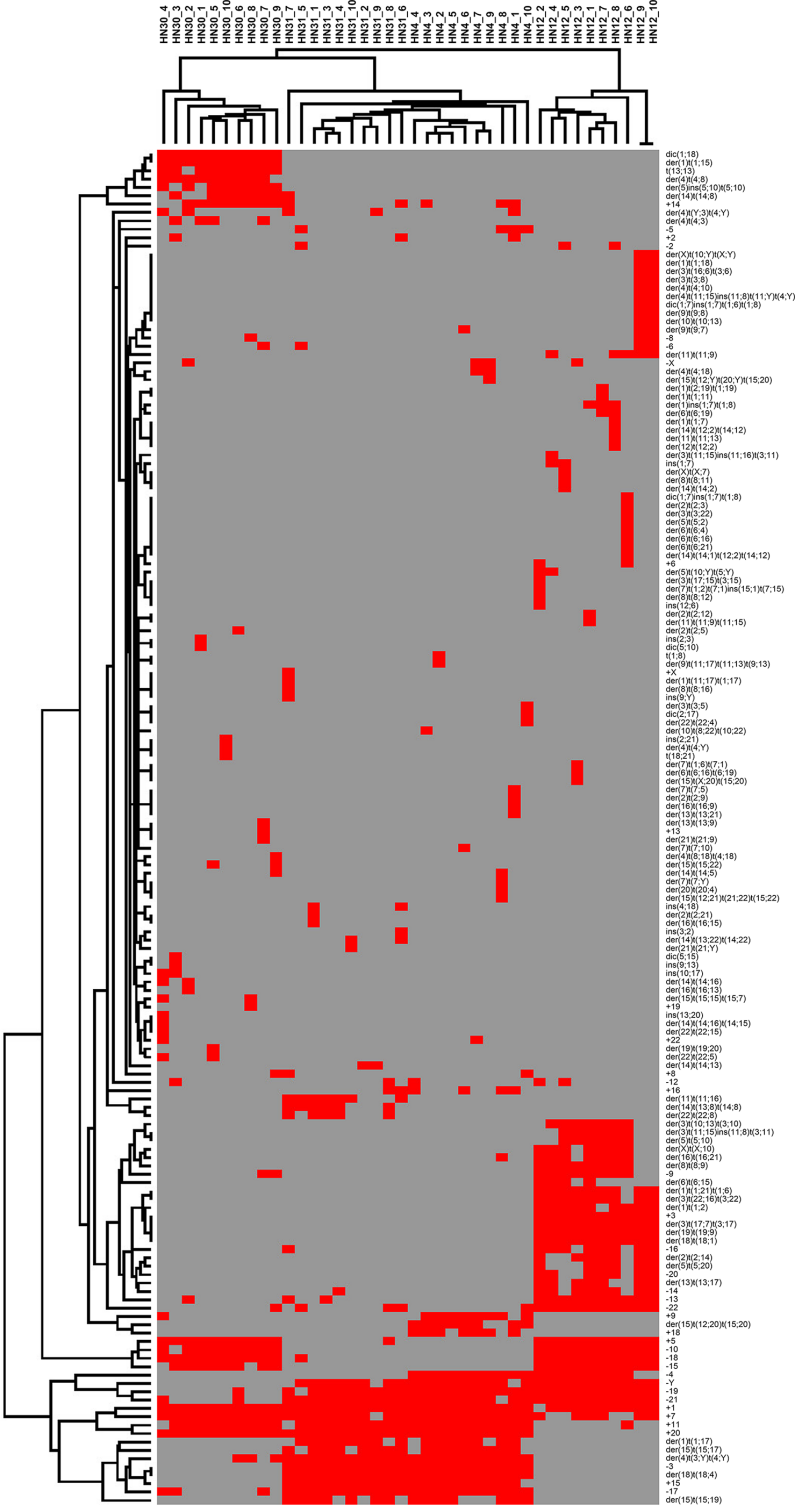
Graphical representation of two-dimensional unsupervised hierarchical clustering of chromosomal aberrations in four head and neck squamous cell carcinoma cell lines (10 metaphases per cell line). Each column refers to a metaphase in each cell line, and each row to type of chromosomal abnormality. Red indicates the presence of each abnormality. Black indicates the absence of each abnormality.

Comparison of hierarchical clustering among the four cell lines showed that all metaphases of HN30 and HN12 were clustered together in each cell line as monophyletic clade (Fig 2). HN31 was closely related to HN4, which shared aberrations: der(1)t(1;17), -3, der(4)t(3;Y)t(4;Y), +15, der(15)t(15;17), der(15)t(15;19), -17, and der(18)t(18;4).

## C-banding

**Fig 3:**
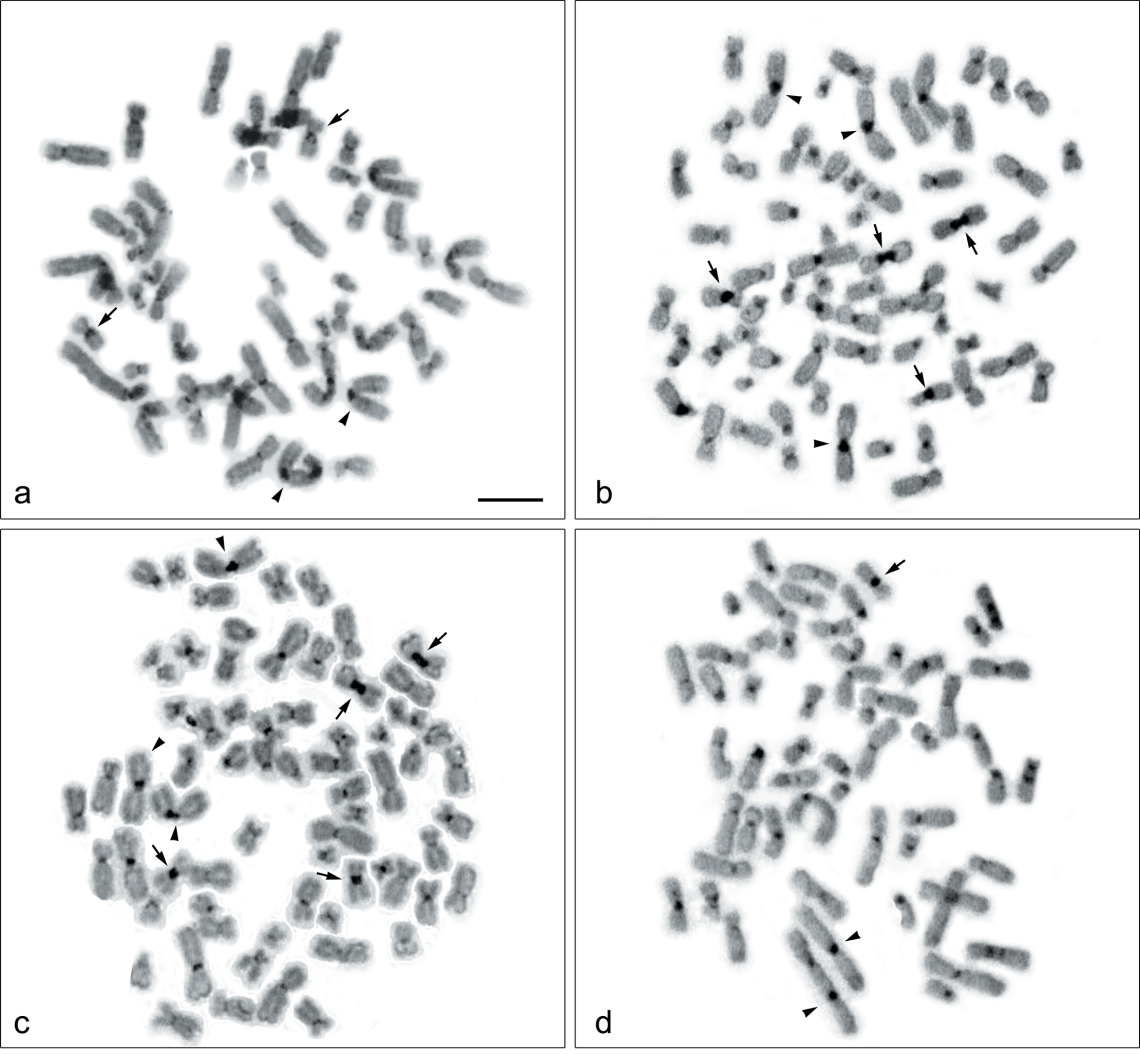
C-banded metaphase spread of four head and neck squamous cell carcinoma cell lines. C-banded metaphase spread of HN30, HN31, HN4, and HN12 are shown in (a, b, c, and d). Arrows indicate chromosome 9, and arrowheads indicate chromosomes 1. Scale bars represent 10 μm.

C-positive heterochromatins were found in the centromeric regions of all chromosomes; however, large C positive-bands were observed on chromosome 9 of HN31 and HN4 (Fig 3B and 3C). Two heterochromatin regions involving translocation between chromosome 9 and 19 were also found in HN12 (Figs 1H and 3D). A dicentric chromosome involving chromosome 1 and 7 was found in HN12 (Figs 1H and 3D), and chromosome 1 and 18 in HN30 (Figs 1B and 3A), all of which showed C-heterochromatin bands in each centromeric region.

### Amplifications and deletions in HNSCC cell lines detected by array comparative genomic hybridization

Genome wide analysis was performed using array CGH to identify 487 amplification-deletion genomic regions based on G-band metaphase chromosomes in the four cell lines: HN30 (149 amplification and 46 deletion regions), HN31 (76 amplification and 6 deletion regions), HN4 (84 amplification and 5 deletion regions), and HN12 (80 amplification and 41 deletion regions). Our data were compared with The Cancer Genome Atlas (TCGA) [29], and the results showed 251 for new CNVs (S1–S5 Tables and S5–S8 Figs).

**Fig 4:**
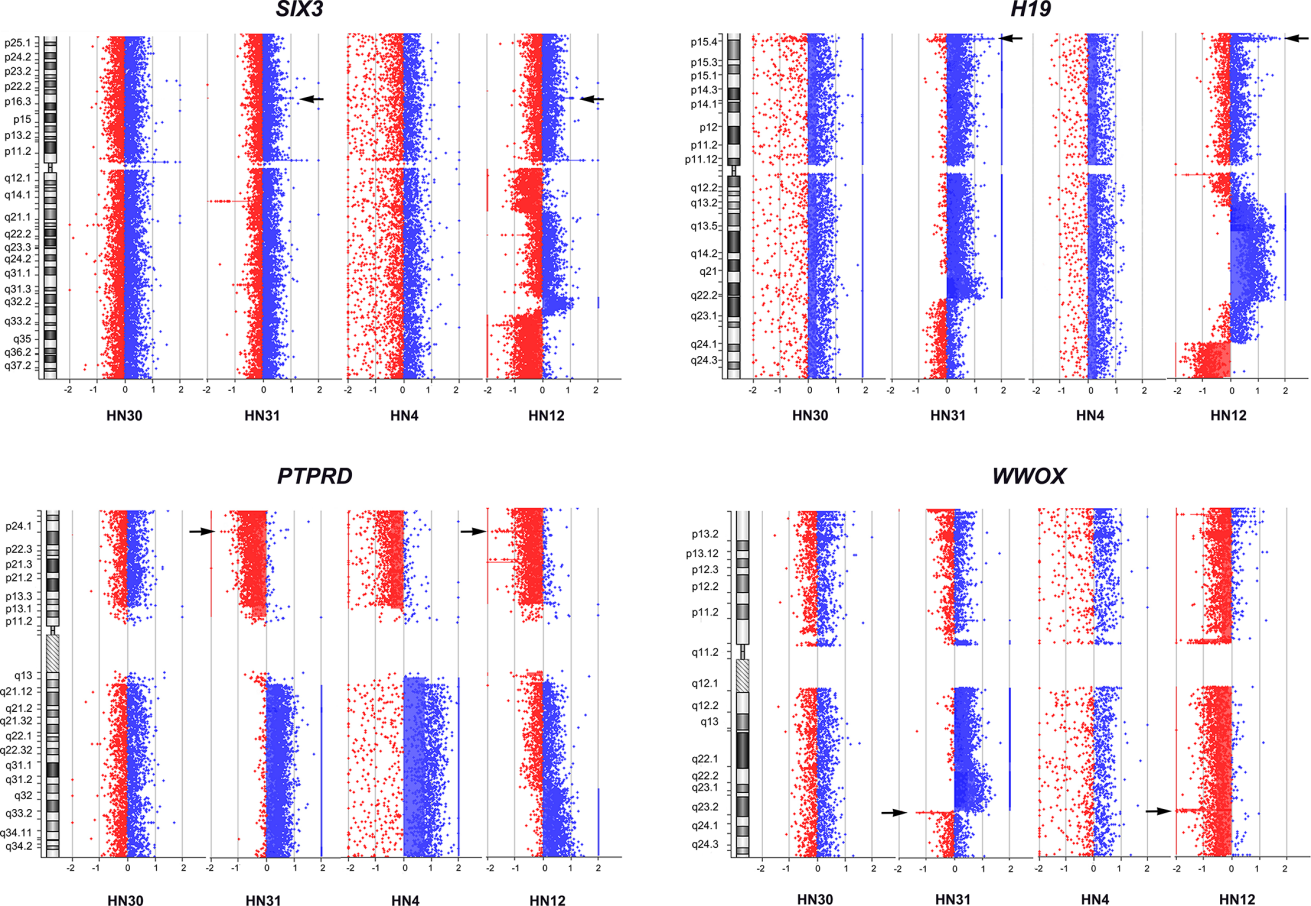
Amplification of *SIX3* and *H19*, and deletion of *PTPRD* and *WWOX* in four head and neck squamous cell carcinoma cell lines. The X-axis represents the normalize log2 ratio fluorescence intensity thresholds -0.9 (loss) and 0.53 (gain), while the Y-axis represents the ideogram of human chromosome. Arrows indicate amplification of *SIX3* (2p21) and *H19* (11p15.5), and deletion of *PTPRD* (9p23) and *WWOX* (16q23.1) in HN31 and HN12 cell lines.

Interestingly, three regions of amplification were found in all cell lines: 7p22.3p11.2, 8q11.23q12.1, and 14q32.33. Comparison of the same patient individual, showed three regions (amplification: 8p22 and 20p13p11.1, and deletion: 4q13.2) found only in both HN30 and HN31, and no amplification and deletion regions were found in HN4 and HN12. Alternatively, one region (deletion: 1p13.2) was detected in both HN30 and HN4, which were derived from primary lesion stages, and five regions (amplification: 2p21 (*SIX3*), 11p15.5 (*H19*), and 11q21q22.3 (*MAML2*, *PGR*, *TRPC6*, *MMP* family), and deletion: 9p23 (*PTPRD*) and 16q23.1 (*WWOX*) were identified in HN31 and HN12, which were lymph node metastatic stages (Fig 4).

### Validation of *SIX3*, *H19*, *PTPRD*, and *WWOX* genes in four HNSCC cell lines using expression levels

**Fig 5:**
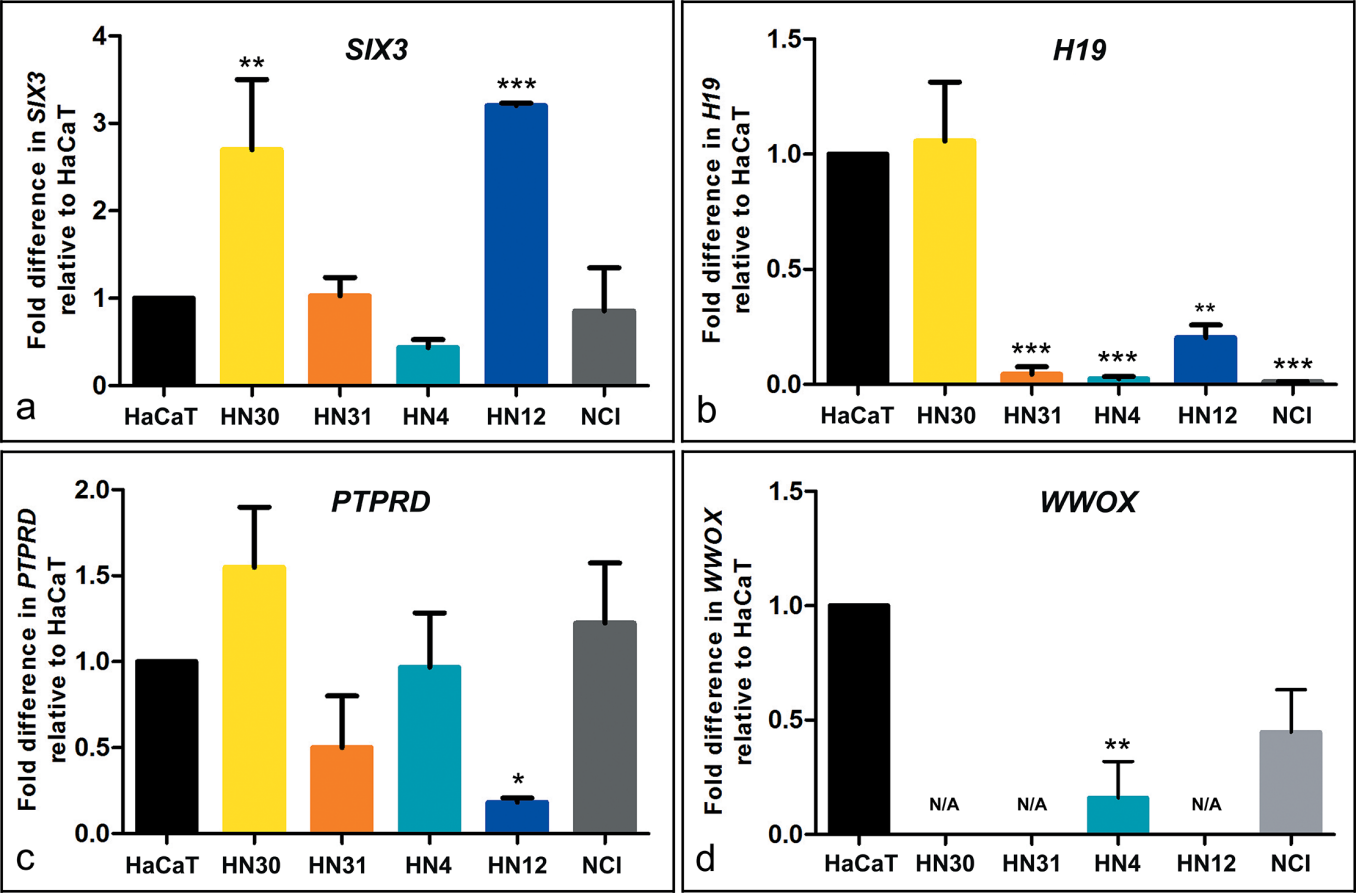
mRNA expression of candidate genes in four head and neck squamous cell carcinoma cell lines and a human mucoepidermoid pulmonary carcinoma NCI as compared with a non-tumorigenic human skin keratinocyte cell line HaCaT by real time quantitative reverse transcription PCR. The X-axis indicates relative expression level to HaCaT, and the Y-axis indicates expression level of each cell line. The level of mRNA expression of *SIX3*, *H19*, *PTPRD*, and *WWOX* are shown in (a, b, c, and d). Data were expressed as mean ± standard deviation. The levels of statistical significance were represented as * for *P* ≤ 0.05, ** for *P* ≤ 0.01, and *** for *P* ≤ 0.001. N/A indicates expression not detectable.

The over-expression of *SIX3* gene was found in HN30 (*P* < 0.01) and HN12 (*P* < 0.001) cell lines, but not for HN31 and HN4. *H19* showed down-regulation in HN31, HN4 (*P* < 0.001), and HN12 (*P* < 0.01). The partial deletion of *PTPRD* exhibited down-regulation in HN12 (*P* < 0.05), and tended to be low level of expression in HN31. No expression level of the *WWOX* gene was found in HN30, HN31, or HN12, and down-regulation in HN4 (*P* < 0.01) (Fig 5).

## Discussion

Genomic alteration of four HNSCC cell lines was characterized by M-FISH and array CGH. Chromosome alteration profiles were analyzed for each cell line, and the types and frequencies of abnormalities were observed using hierarchical clustering. No shared structural aberration case was found in HN30 and HN31, HN4 and HN12, or in primary lesion (HN30 and HN4) and metastatic lesion (HN31 and HN12). However, most of the cell lines shared numerical aberrations with gain of chromosome 1, 7, and 11. Basically, both chromosome 7 and 11 contain genes that are involved in the tumor progression such as *EGFR*, *HGF*, *HOX*, *BRAF*, *PAFAH1B2*, *FLI1*, and *ETS1* [30–35], in particular *LAMB1*, *MET*, *MMP1*, *CCND1*, *ORAOV1*, *FADD*, *PPFIA1*, and *CTTN* genes which related to HNSCC [36–38]. In addition, the gain of chromosome 7 was predominantly found in colorectal cancer, glioblastoma, and renal cell carcinoma [32, 39, 40], and the gain of chromosome 11 was reported in ovarian cancer and myeloid leukemia [41, 42]. This suggests that the gain of both chromosome 7 and 11 have significance in carcinogenesis, when compared to other chromosomes in numerical aberration. Further investigations are required to elucidate the relationship between chromosome 7 and 11. Most numerical and structural aberrations were often found in chromosome 1, which related to genes (*COL11A1*, *GSTM3*, *CKS1B*, *CDT2*, *ETV3*, *ELF3*, *ASPM*, *KIF14*, *NEK2*, *DTL*, *CENPF*, *CKS1B*, *EXO1*, *GPR137B*, and *CD1D*) in carcinogenesis [43–48]. No shared chromosome number aberration case was found in HN30 and HN31, or in primary lesion (HN30 and HN4) and metastatic lesion (HN31 and HN12), except for the loss of chromosome 4 found in HN4 and HN12. This chromosome contains genes that are involved in carcinogenesis such as *PDLIM3*, *ANK2*, *SORBS2*, and *PPARGCIA* [49]. Hierarchical clustering analysis revealed several clusters in each cell line, suggesting that tumor heterogeneity was found in both primary and metastatic lesions. Surprisingly, the consequence of clustering with four cell lines suggested that three major clusters were grouped as HN30, HN12, and HN31-HN4. HN31 was closely related to HN4, which shared eight chromosome alteration cases, most of which were structural aberrations. This suggests the possibility of complex structural aberration groupings of HN31 and HN4 into the same cluster analysis. Although this cluster relationship was not correlated to the tumor clinical stages, previous studies showed the same phenotypic progress with the low level of *MMP9* (20q13.12) activity in the potential of HN31 and HN4 invasion, but not for HN30 and HN12 [16, 50]. Moreover, large C-positive heterochromatins were found in the centromeric region of chromosome 9 for both HN31 and HN4, which suggests the specific amplification of repetitive sequences in these two cell lines. However, the results of array CGH showed many different CNVs between the two cell lines (HN31 and HN4). The grouping of the two cell lines might result from analyzing a few markers (chromosome alteration types) that make close relationship unintentional.

On the basis of chromosome alterations in this study, the presence of tumor progression from a single precursor cell might be explained by the occurrence of aneuploidy, polyploidy, and deletion, followed by various chromosome structural aberrations such as translocations and insertions [51–53]. This agreed with similar numbers of numerical aberration among four cell lines (HN30 = 25 cases, HN31 = 26 cases, HN4 = 22 cases, and HN12 = 24 cases). By contrast, the number of structural aberration cases tended to occur in HN12 (58 cases), which is the metastatic cell line in stage four of cancer. This suggests that structural alterations were predominantly observed in invasive malignancy [54].

Genome wide profiling of the four cancer cell lines was alternatively carried out using high resolution array CGH, at a resolution of approximately 0.06 kb with 180,000 probes to determine CNVs. Large CNVs were found in 5p15.33p11 (amplification), 7p22.3p11.2 (amplification), 8q11.23q24.3 (amplification), which were also reported as chromosome arm level CNVs in the previous work [55]. We were able to identify large additional regions: 9q13q34.3 (amplification), 10p15.3p11.1 (deletion), 14q11.2q32.33 (amplification), 20p13p11.1 (amplification), and 20q11.21q13.33 (amplification). Most agreed with the results of M-FISH analysis except for 8q11.23q24.3 (amplification) which might be segmental duplication (intrachromosomal aberration) and M-FISH could not detect. A few well documented CNVs were identified in HNSCC that included amplification of 3q27.3q28 (*CLDN1*), 7q11.1q31.31 (*LAMB1*), 8q11.23q24.3 (*MYC* and *PTK2*), 11q13.2q22.3 (*PPFIA1*, *CTTN*, *FGF3*, *FGF4*, *FADD*, *CCND1*, *MMP7*, *MMP20*, *MMP27*, *MMP8*, *MMP10*, *MMP1*, *MMP3*, *MMP12*, and *MMP13*), and deletion of 1p21.1p11.2 (*COL11A1* and *GSTM3*), 3p14.2 (*FHIT*), 3p26.1 (*GRM7*), 3p13 (*FOXP1*), 9p21.3 (*CDKN2A* and *CDKN2B*), and 18q12.1q23 (*SERPINB2*) [56–61], all of which were involved in tumor cell proliferation and angiogenesis in oral carcinogenesis [50, 62–65]. Notably, the most complex patterns of amplifications or deletions were identified as 22q11.21 region in HN30, HN31, and HN12, and this region comprises *TBX1* that also involved negative regulator of tumor cell growth [66]. Another site was observed in the 4q13.2 region of HN31 and HN12, and found both amplification and deletion in HN30 in this study. This phenomenon was also observed in studies by Ambatipudi et al. [61] and Vincent-Chong et al. [55] which suggests possible tumor heterogeneity. The 4q13.2 region contained the *UGT2B17* gene that involved tongue and larynx SCC. The copy number polymorphisms of this gene in cancer are required to understand the role of this gene in HNSCC.

Three common regions of amplification of chromosome segments found in all four cell lines were related to *PDGFA*, *MAD1L1*, *ACTB*, *IL6*, *CYCS*, *GLI3*, *DDC*, *EGFR*, *SDK1*, *KIAA0125*, and *ADAM6* genes. These genes involved tumorigenesis and inflammation in various tumor types [30, 67–77]. Three regions: 3p14.2 (deletion), 9p21.2q21.31 (amplification), and 20q13.12 (amplification) were commonly found in HN4 and HN31 as the same cluster in our analysis, and five genes (*FHIT*, *MMP9*, *GNA14*, *GNAQ*, and *PSAT1*) were involved in tumorigenesis in lung cancer and colon cancer [50, 63, 78–80]. One region: 1p13.2 (deletion) was found in both HN30 and HN4, derived from primary lesion stages, and *SYCP1* gene involved in carcinogenesis in medulloblastoma [81]. Five regions: 2p21 (amplification), 11p15.5 (amplification), 11q21q22.3 (amplification), 9p23 (deletion), and 16q23.1 (deletion) were commonly found in HN31 and HN12. These genes: *SIX3*, *H19*, *PTPRD*, *WWOX*, *MAML2*, *PGR*, *TRPC6*, *MMP20*, *MMP13*, *MMP1*, and *MMP3* were involved in tumor progression of mucoepidermoid carcinoma, prostate adenocarcinoma, glioblastoma, pancreatic cancer, lung cancer, and epithelial breast cancer [50, 82–90]. To extensively investigate the regulation of these functional genes, four of five regions: 2p21 (*SIX3*), 11p15.5 (*H19*), 9p23 (*PTPRD*), and 16q23.1 (*WWOX*) that showed amplification or deletion in both HN31 and HN12 were examined for the level of gene expression by qRT-PCR. Sine oculis homeobox homolog 3 (*SIX3*) gene plays a role in embryogenesis in vertebrates, as a transcription repressor to regulate proliferation and metastasis related genes [89]. Basically, genomic amplification which increases gene dosage can lead to over-expression at mRNA level. This agreed with the level of *SIX3* expression in HN12, but not for HN31 whose *SIX3* expression was the same level with the control group (HaCaT and NCI). Over-expression was also found in HN30, though no amplification or deletion were found in array CGH. This suggests that the number of *SIX3* gene copy is not related to gene expression in HNSCC. The mechanism of *SIX3* over-expression remains unclear, and further upstream analysis is required to elucidate its role in HNSCC. A similar case was found in *H19*, which is an imprinted oncofetal gene with maternal expression in fetal tissues. It is expressed in the first stage of embryogenesis, but down-regulated or turned off in most tissues after birth as a tumor suppressor role [84, 91]. Studies of various tumors have demonstrated an overexpression of the *H19* RNA when compared to normal tissues, leading to targeting for gene therapy [92, 93]. However, both HN31 and HN12 showed very low levels of expression for *H19*. This pattern was also found in HN4 and NCI, whereas HN30 showed the same expression level as found in HaCaT. Human bladder carcinoma derived from adult bladder mucosa cells had also lost their ability to express *H19* gene. However, the significant increase of *H19* during the process of tumor formation in mice was accomplished by injected carcinoma cell lines [93]. This suggests that *H19* is expressed at substantial levels in several different tumor types [94], and might re-activate expression within the process of tumorigenesis. Protein tyrosine phosphatase receptor type D (*PTPRD*) is a receptor type tyrosine-protein phosphatase (membrane-integral enzymes) that helps to remove phosphate groups from specific proteins [95]. Mutation or epigenetic silencing of *PTPRD* has been reported for the loss of tumor suppressor role in several cancers [96, 97]. In this study, although trisomy was found on chromosome 9 in HN31 and HN12, the presence of deletion still occurred at *PTPRD*. This agreed with the loci of genomic deletion which reduced gene dosage and resulted in down-expression at mRNA level in metastatic cells (HN31 and HN12) compared to less invasive cells (HN30 and HN4), suggesting that the loss of *PTPRD* function involved cell migration [98].

Chromosome region 16q23.3q24.1 is the same locus with the fragile site (FRA16D) and often breaks down in several cancer types [82, 99]. WW domain containing oxidoreductase (*WWOX*) gene, which is also located in these regions is considered an important role in tumor suppression through transcriptional repression and apoptosis [100]. Mutation and epigenetic silencing of the *WWOX* gene have been reported in various cancers [101–103], and the loss of *WWOX* expression is an important step in carcinogenesis [101, 104]. Although deletions of 16q23.1 were not found in HN30 and HN4, low levels of *WWOX* expression were observed in all cell lines. This agreed with many tumor types affected by *WWOX* deletions [102], which suggests that the loss of *WWOX* at both genomic or expression level is an important role of cell development in carcinogenesis. This gene is also a promising target to develop gene therapy plan [100, 105].

In this study, the genome profiles of primary tumor and metastasis derived cell lines isolated from the same patients were addressed by karyotyping, molecular cytogenetics, and array CGH. Results suggest that segments/regions corresponded to the stage of tumor progression/carcinogenesis. The presence of CNVs are further needed to gain insight into the association of clinical outcomes in HNSCC, compared to other cancers. Although the level of gene expression is not significantly different between the primary and metastatic pairs for any of the four genes, it is also interesting to understand the role of *PTPRD* in invasive processes and *WWOX* genes in carcinogenesis, or as biomarkers to reflect molecular progression in pathogenesis. Transcriptomics and proteomics analysis for these four cell lines are required to gain insight into validating relevant targets. Many candidate genes from the present genomic alteration data are also required to further examine the precision, incidence, and significant target in HNSCC.

## Supporting Information

S1 Fig Hierarchical cluster analysis of the presence or absence of chromosomal aberrations observed in HN30 cell line. Each column refers to a metaphase and each row to type of a chromosomal abnormality. Red indicates the presence of each abnormality. Black indicates the absence of each abnormality.
(TIF)

S2 Fig Hierarchical cluster analysis of the presence or absence of chromosomal aberrations observed in HN31 cell line. Each column refers to a metaphase and each row to type of a chromosomal abnormality. Red indicates the presence of each abnormality. Black indicates the absence of each abnormality.
(TIF)

S3 Fig Hierarchical cluster analysis of the presence or absence of chromosomal aberrations observed in HN4 cell line. Each column refers to a metaphase and each row to type of a chromosomal abnormality. Red indicates the presence of each abnormality. Black indicates the absence of each abnormality.
(TIF)

S4 Fig Hierarchical cluster analysis of the presence or absence of chromosomal aberrations observed in HN12 cell line. Each column refers to a metaphase and each row to type of a chromosomal abnormality. Red indicates the presence of each abnormality. Black indicates the absence of each abnormality.
(TIF)

S5 Fig Representative genomic profile of HN30. Detailed genomic profiles on chromosome, the X-axis represents the normalize log2 ratio fluorescence intensity thresholds -0.9 (loss) and 0.53 (gain), while the Y-axis represents the ideogram of human chromosome.
(JPG)

S6 Fig Representative genomic profile of HN31. Detailed genomic profiles on chromosome, the X-axis represents the normalize log2 ratio fluorescence intensity thresholds -0.9 (loss) and 0.53 (gain), while the Y-axis represents the ideogram of human chromosome.
(JPG)

S7 Fig Representative genomic profile of HN4. Detailed genomic profiles on chromosome, the X-axis represents the normalize log2 ratio fluorescence intensity thresholds -0.9 (loss) and 0.53 (gain), while the Y-axis represents the ideogram of human chromosome.
(JPG)

S8 Fig Representative genomic profile of HN12. Detailed genomic profiles on chromosome, the X-axis represents the normalize log2 ratio fluorescence intensity thresholds -0.9 (loss) and 0.53 (gain), while the Y-axis represents the ideogram of human chromosome.
(JPG)

S1 Table Genome view of chromosome copy number variation (CNV). (DOCX)

S2 Table Genome view of chromosome copy number variation (CNV) in HN30 cell line. (DOCX)

S3 Table Genome view of chromosome copy number variation (CNV) in HN31 cell line. (DOCX)

S4 Table Genome view of chromosome copy number variation (CNV) in HN4 cell line. (DOCX)

S5 Table Genome view of chromosome copy number variation (CNV) in HN12 cell line. (DOCX)

## References

[1] Jones PA, Baylin SB. The fundamental role of epigenetic events in cancer. Nat Rev Genet. 2002; 3: 415–428. 10.1038/nrg816 . 12042769

[2] Albertson DG, Collins C, McCormick F, Gray JW. Chromosome aberrations in solid tumors. Nat Genet. 2003; 34: 369–376. 10.1038/ng1215 . 12923544

[3] Perez-Ordonez B, Beauchemin M, Jordan RCK. Molecular biology of squamous cell carcinoma of the head and neck. J Clin Pathol. 2006; 59: 445–453. 10.1136/jcp.2003.007641 . 16644882 PMC1860277

[4] Chaturvedi AK, Anderson WF, Tieulent JL, Curado MP, Ferlay J, Franceschi S, et al Worldwide trends in incidence rates for oral cavity and oropharyngeal cancers. J Clin Oncol. 2013; 31: 4550–4559. 10.1200/JCO.2013.50.3870 . 24248688 PMC3865341

[5] Pruegsanusak K, Peeravut S, Leelamanit V, Sinkijcharoenchai W, Jongsatitpaiboon J, Phungrassami T, et al Survival and prognostic factors of different sites of head and neck cancer: an analysis from Thailand. Asian Pac J Cancer P. 2012; 13: 885–890. 10.7314/APJCP.2012.13.3.885 . 22631666

[6] Vatanasapt P, Thanaviratananich S, Ratanaanekchai T, Thepsuthammarat K. The burden of head and neck cancers in Thailand. J Med Assoc Thai. 2012; 7: 182–189. . 23130452

[7] Walsh JE, Lathers DM, Chi AC, Gillespie MB, Day TA, Young MR. Mechanisms of tumor growth and metastasis in head and neck squamous cell carcinoma. Curr Treat Options Oncol. 2007; 8: 227–238. 10.1007/s11864-007-0032-2 . 17712533

[8] Zhang P, Mirani N, Baisre A, Fernandes H. Molecular heterogeneity of head and neck squamous cell carcinoma defined by next-generation sequencing. Am J Pathol. 2014; 184: 1323–1330. 10.1016/j.ajpath.2014.01.028 . 24767105

[9] Mohan M, Jagannathan N. Oral field cancerization: an update on current concepts. Oncol Rev. 2014; 8: 244 10.4081/oncol.2014.244 . 25992232 PMC4419611

[10] Shlush LI, Hershkovitz D. Clonal evolution models of tumor heterogeneity. Am Soc Clin Oncol Educ Book. 2015; 2015: e662–e665. 10.14694/EdBook_AM.2015.35.e662 . 25993239

[11] Chapman A, del Ama LF, Ferguson J, Kamarashev J, Wellbrock C, Hurlstone D. Heterogeneous tumor subpopulations cooperate to drive invasion. Cell Rep. 2014; 8: 688–695. 10.1016/j.celrep.2014.06.045 . 25066122 PMC4542310

[12] Braakhuis BJ, Tabor MP, Leemans CR, van der Waal I, Snow GB, Brakenhoff RH. Second primary tumors and field cancerization in oral and oropharyngeal cancer: molecular techniques provide new insights and definitions. Head Neck. 2002; 24: 198–206. 10.1002/hed.10042 . 11891950

[13] Squire JA, Bayani J, Luk C, Unwin L, Tokunaga J, MacMillan C, et al Molecular cytogenetic analysis of head and neck squamous cell carcinoma: By comparative genomic hybridization, spectral karyotyping, and expression array analysis. Head Neck. 2002; 24: 874–887. 10.1002/hed.10122 . 12211052

[14] Sethi S, Benninger MS, Lu M, Havard S, Worsham MJ. Noninvasive molecular detection of head and neck squamous cell carcinoma: an exploratory analysis. Diagn Mol Pathol. 2009; 18: 81–87. 10.1097/PDM.0b013e3181804b82 . 19430297 PMC2693294

[15] Koontongkaew S, Amornphimoltham P, Yapong B. Tumor-stroma interactions influence cytokine expression and matrix metalloproteinase activities in paired primary and metastatic head and neck cancer cells. Cell Biol Int. 2009; 33: 165–173. 10.1016/j.cellbi.2008.10.009 . 18996211

[16] Koontongkaew S, Amornphimoltham P, Monthanpisut P, Saensuk T, Leelakriangsak M. Fibroblasts and extracellular matrix differently modulate MMP activation by primary and metastatic head and neck cancer cells. Med Oncol. 2012; 29: 690–703. 10.1007/s12032-011-9871-6 . 21380786

[17] Cardinali M, Pietraszkiewicz H, Ensley JF, Robbins K. Tyrosine phosphorylation as a marker for aberrantly regulated growth-promoting pathways in cell lines derived from head and neck malignancies. Int J Cancer. 1995; 61: 98–103. . 7705939 10.1002/ijc.2910610117

[18] Supikamolseni A, Ngaoburanawit N, Sumontha M, Chanhome L, Suntrarachun S, Peyachoknagul S, et al Molecular barcoding of venomous snakes and species-specific multiplex PCR assay to identify snake groups for which antivenom is available in Thailand. Genet Mol Res. 2015; 14: 13981–13997. 10.4238/2015.October.29.18 . 26535713

[19] Srikulnath K, Uno Y, Nishida C, Ota H, Matsuda Y. Karyotype reorganization in the Hokou Gecko (*Gekko hokouensis*, Gekkonidae): The process of microchromosome disappearance in Gekkota. PLOS ONE. 2015; 10: e0134829 doi: 10.1371/ journal.pone.0134829 . 26241471 10.1371/journal.pone.0134829 PMC4524605

[20] Boukamp P, Petrussevska RT, Breitkreutz D, Hornung J, Markham A, Fusenig NE. Normal keratinization in a spontaneously immortalized aneuploid human keratinocyte cell line. J Cell Biol. 1988; 106: 761–771. . 2450098 10.1083/jcb.106.3.761 PMC2115116

[21] Gooding RP, Bybee A, Cooke F, Little A, Marsh SG, Coelho E, et al Phenotypic and molecular analysis of six human cell lines derived from patients with plasma cell dyscrasia. Br J Haematol. 1999; 106: 669–681. 10.1046/j.1365-2141.1999.01602.x . 10468855

[22] Geigl JB, Uhrig S, Speicher MR. Multiplex-fluorescence *in situ* hybridization for chromosome karyotyping. Nat Protoc. 2006; 1: 1172–1184. 10.1038/nprot.2006.160 . 17406400

[23] Gribble SM, Wiseman FK, Clayton S, Prigmore E, Langley E, Yang F, et al Massively parallel sequencing reveals the complex structure of an irradiated human chromosome on a mouse background in the Tc1 model of down syndrome. PLOS ONE. 2013; 8: e60482 10.1371/journal.pone.0060482 . 23596509 PMC3626651

[24] Sumner AT. A simple technique for demonstrating centromeric heterochromatin. Exp Cell Res. 1972; 75: 304–306. . 4117921 10.1016/0014-4827(72)90558-7

[25] de Hoon MJL, Imoto S, Nolan J, Miyano S. Open source clustering software. Bioinformatics. 2004; 20: 1453–1454. 10.1093/bioinformatics/bth078 . 14871861

[26] Saldanha AJ. Java Treeview—extensible visualization of microarray data. Bioinformatics. 2004; 20: 3246–3248. 10.1093/bioinformatics/bth349 . 15180930

[27] Wattanapanitch M, Klincumhom N, Potirat P, Amornpisutt R, Lorthongpanich C, U-pratya Y, et al Dual small-molecule targeting of SMAD signaling stimulates human induced pluripotent stem cells toward neural lineages. PLOS ONE. 2014; 9: e106952 10.1371/journal.pone.0106952 PMCID: PMC4160199. 25207966 PMC4160199

[28] Livak KJ, Schmittgen TD. Analysis of relative gene expression data using real-time quantitative PCR and the 2^−∆∆CT^ method. Methods. 2001; 25: 402–408. 10.1006/meth.2001.1262 . 11846609

[29] Cancer Genome Atlas Network. Comprehensive genomic characterization of head and neck squamous cell carcinomas. Nature. 2015; 517: 576–582. 10.1038/nature14129 . 25631445 PMC4311405

[30] Kanata K, AL Sheikh Ali M, Nagatsuka H, Liu GR, Ryoun PH, Takayama M, et al Detecting the (Epidermal Growth Factor Receptor) EGFR gene amplification in oral carcinogenesis. J Hard Tissue Biology. 2005; 14: 251–252. 10.2485/jhtb.14.251

[31] Ferreiro JF, Morscio J, Dierickx D, Marcelis L, Verhoef G, Vandenberghe P, et al Post-transplant molecularly defined Burkitt lymphomas are frequently MYC-negative and characterized by the 11q-gain/loss pattern. Haematologica. 2015; 100: e275–e279. 10.3324/haematol.2015.124305 . 25795716 PMC4486241

[32] Kurscheid S, Bady P, Sciuscio D, Samarzija I, Shay T, Vassallo I, et al Chromosome 7 gain and DNA hypermethylation at the *HOXA10* locus are associated with expression of a stem cell related HOX-signature in glioblastoma. Genome Biol. 2015; 16: 16 10.1186/s13059-015-0583-7 . 25622821 PMC4342872

[33] Sasaki H, Maekawa M, Tatematsu T, Okuda K, Moriyama S, Yano M, et al Increased *BRAF* copy number in lung adenocarcinoma. Oncol Lett. 2015; 9: 709–712. 10.3892/ol.2014.2719 . 25621040 PMC4301492

[34] Seneviratne D, Ma J, Tan X, Kwon YK, Muhammad E, Melhem M, et al Genomic instability causes HGF gene activation in colon cancer cells, promoting their resistance to necroptosis. Gastroenterology. 2015; 148: 181–191. 10.1053/j.gastro.2014.09.019 . 25244939 PMC4274190

[35] Higaki E, Kuwata T, Nagatsuma A K, Nishida Y, Kinoshita T, Aizawa M, et al Gene copy number gain of *EGFR* is a poor prognostic biomarker in gastric cancer: evaluation of 855 patients with bright-field dual in situ hybridization (DISH) method. Gastric Cancer. 2016; 19: 63–73. 10.1007/s10120-014-0449-9 . 25487305

[36] Hensen EF, De Herdt MJ, Goeman JJ, Oosting J, Smit VT, Cornelisse CJ, et al Gene-expression of metastasized versus non-metastasized primary head and neck squamous cell carcinomas: a pathway-based analysis. BMC Cancer. 2008; 8: 168 10.1186/1471-2407-8-168 . 18544165 PMC2438367

[37] Jarmuz-Szymczak M, Pelinska K, Kostrzewska-Poczekaj M, Bembnista E, Giefing M, Brauze D, et al Heterogeneity of 11q13 region rearrangements in laryngeal squamous cell carcinoma analyzed by microarray platforms and fluorescence in situ hybridization. Mol Biol Rep. 2013; 40: 4161–4171. 10.1007/s11033-013-2496-4 . 23652995

[38] Brauswetter D, Dános K, Gurbi B, Félegyházi ÉF, Birtalan E, Meggyesházi N, et al Copy number gain of *PIK3CA* and *MET* is associated with poor prognosis in head and neck squamous cell carcinoma. Virchows Arch. 2016; 468: 579–587. 10.1007/s00428-016-1905-1 . 26832731

[39] Kohn L, Svenson U, Ljungberg B, Roos G. Specific genomic aberrations predict survival, but low mutation rate in cancer hot spots, in clear cell renal cell carcinoma. Appl Immunohistochem Mol Morphol. 2015; 23: 334–342. 10.1097/PAI.0000000000000087 . 24992170 PMC4431677

[40] Seo AN, Park KU, Choe G, Kim WH, Kim DW, Kang SB, et al Clinical and prognostic value of *MET* gene copy number gain and chromosome 7 polysomy in primary colorectal cancer patients. Tumour Biol. 2015; 36: 9813–9821. 10.1007/s13277-015-3726-2 . 26159851

[41] Foulkes WD, Campbell IG, Stamp GW, Trowsdale J. Loss of heterozygosity and amplification on chromosome 11q in human ovarian cancer. Br J Cancer. 1993; 67: 268–273. PMCID: PMC1968175. 8094291 10.1038/bjc.1993.51 PMC1968175

[42] Krum EK, Hamed T, Yamamoto M, de Lourdes Chauffaille M. Isolated trisomy 11 in de novo acute myeloid leukemia. Rev Bras Hematol Hemoter. 2008; 30: 253–255.

[43] Zhang SG, Song WQ, Gao YT, Yang B, Du Z. *CD1d* gene is a target for a novel amplicon at 1q22–23.1 in human hepatocellular carcinoma. Mol Biol Rep. 2010; 37: 381–387. 10.1007/s11033-009-9817-7 . 19757161

[44] Mackintosh C, Ordóñez JL, García-Domínguez DJ, Sevillano V, Llombart-Bosch A, Szuhai K, et al 1q gain and *CDT2* overexpression underlie an aggressive and highly proliferative form of Ewing sarcoma. Oncogene. 2012; 31: 1287–1298. 10.1038/onc.2011.317 . 21822310

[45] Mesquita B, Lopes P, Rodrigues A, Pereira D, Afonso M, Leal C, et al Frequent copy number gains at 1q21 and 1q32 are associated with overexpression of the ETS transcription factors *ETV3* and *ELF3* in breast cancer irrespective of molecular subtypes. Breast Cancer Res Treat. 2013; 138: 37–45. 10.1007/s10549-013-2408-2 . 23329352

[46] Muthuswami M, Ramesh V, Banerjee S, Viveka Thangaraj S, Periasamy J, Bhaskar Rao D, et al Breast tumors with elevated expression of 1q candidate genes confer poor clinical outcome and sensitivity to Ras/PI3K inhibition. PLOS ONE. 2013; 8: e77553 10.1371/journal.pone.0077553 . 24147022 PMC3798322

[47] Stella F, Pedrazzini E, Baialardo E, Fantl DB, Schutz N, Slavutsky I. Quantitative analysis of *CKS1B* mRNA expression and copy number gain in patients with plasma cell disorders. Blood Cells Mol Dis. 2014; 53: 110–117. 10.1016/j.bcmd.2014.05.006 . 24973170

[48] Andrade VP, Morrogh M, Qin LX, Olvera N, Giri D, Muhsen S, et al Gene expression profiling of lobular carcinoma *in situ* reveals candidate precursor genes for invasion. Mol Oncol. 2015; 9: 772–782. 10.1016/j.molonc.2014.12.005 . 25601220 PMC4387062

[49] Stein L, Rothschild J, Luce J, Cowell JK, Thomas G, Bogdanova TI, et al Copy number and gene expression alterations in radiation-induced papillary thyroid carcinoma from chernobyl pediatric patients. Thyroid. 2010; 20: 475–487. 10.1089/thy.2009.0008 . 19725780

[50] Gialeli C, Theocharis AD, Karamanos NK. Roles of matrix metalloproteinases in cancer progression and their pharmacological targeting. FEBS J. 2011; 278: 16–27. 10.1111/j.1742-4658.2010.07919.x . 21087457

[51] Kost-Alimova M, Fedorova L, Yang Y, Klein G, Imreh S. Microcell-mediated chromosome transfer provides evidence that polysomy promotes structural instability in tumor cell chromosomes through asynchronous replication and breakage within late-replicating regions. Genes Chromosomes Cancer. 2004; 40: 316–324. 10.1002/gcc.20054 . 15188454

[52] Janssen A, van der Burg M, Szuhai K, Kops GJ, Medema RH. Chromosome segregation errors as a cause of DNA damage and structural chromosome aberrations. Science. 2011; 333: 1895–1898. 10.1126/science.1210214 . 21960636

[53] Burrell RA, McClelland SE, Endesfelder D, Groth P, Weller MC, Shaikh N, et al Replication stress links structural and numerical cancer chromosomal instability. Nature. 2013; 494: 492–496. 10.1038/nature11935 . 23446422 PMC4636055

[54] Qin Lun-Xiu. Chromosomal aberrations related to metastasis of human solid tumors. World J Gastroenterol. 2002; 8: 769–776. 10.3748/wjg.v8.i5.769 PMCID: PMC4656559. 12378613 PMC4656559

[55] Vincent-Chong VK, Anwar A, Karen-Ng LP, Cheong SC, Yang Y-H, Pradeep PJ, et al Genome wide analysis of chromosomal alterations in oral squamous cell carcinomas revealed over expression of *MGAM* and *ADAM9*. PLOS ONE. 2013; 8: e54705 10.1371/journal.pone.0054705 . 23405089 PMC3566089

[56] Garnis C, Campbell J, Zhang L, Rosin MP, Lam WL. OCGR array: an oral cancer genomic regional array for comparative genomic hybridization analysis. Oral Oncol. 2004; 40: 511–519. 10.1016/j.oraloncology.2003.10.006 . 15006624

[57] Snijders AM, Schmidt BL, Fridlyand J, Dekker N, Pinkel D, Jordan RC, et al Rare amplicons implicate frequent deregulation of cell fate specification pathways in oral squamous cell carcinoma. Oncogene. 2005; 24: 4232–4242. 10.1038/sj.onc.1208601 . 15824737

[58] Liu CJ, Lin SC, Chen YJ, Chang KM, Chang KW. Array-comparative genomic hybridization to detect genomewide changes in microdissected primary and metastatic oral squamous cell carcinomas. Mol Carcinog. 2006; 45: 721–731. 10.1002/mc.20213 . 16676365

[59] Järvinen AK, Autio R, Kilpinen S, Saarela M, Leivo I, Grénman R, et al High-resolution copy number and gene expression microarray analyses of head and neck squamous cell carcinoma cell lines of tongue and larynx. Genes Chromosomes Cancer. 2008; 47: 500–509. 10.1002/gcc.20551 . 18314910

[60] Freier K, Knoepfle K, Flechtenmacher C, Pungs S, Devens F, Toedt G, et al Recurrent copy number gain of transcription factor *SOX2* and corresponding high protein expression in oral squamous cell carcinoma. Genes Chromosomes Cancer. 2010; 49: 9–16. 10.1002/gcc.20714 . 19787784

[61] Ambatipudi S, Gerstung M, Gowda R, Pai P, Borges AM, Schäffer AA, et al Genomic profiling of advanced-stage oral cancers reveals chromosome 11q alterations as markers of poor clinical outcome. PLOS ONE. 2011; 6: e17250 10.1371/journal.pone.0017250 PMCID: PMC3046132. 21386901 PMC3046132

[62] Mao L, Fan YH, Lotan R, Hong WK. Frequent abnormalities of *FHIT*, a candidate tumor suppressor gene, in head and neck cancer cell lines. Cancer Res. 1996; 56: 5128–5131. . 8912845

[63] Croce CM, Sozzi G, Huebner K. Role of *FHIT* in human cancer. J Clin Oncol. 1999; 17: 1618–1624. . 10334551 10.1200/JCO.1999.17.5.1618

[64] Kornberg LJ. Focal adhesion kinase and its potential involvement in tumor invasion and metastasis. Head Neck. 1998; 20: 745–752. 10.1002/(sici)1097-0347(199812)20:8<745::aid-hed14>3.3.co;2-q . 9790298

[65] Dang CV. MYC, metabolism, cell growth, and tumorigenesis. Cold Spring Harb Perspect Med. 2013; 3: a014217 10.1101/cshperspect.a014217 . 23906881 PMC3721271

[66] Trempus CS, Wei S-J, Humble MM, Dang H, Bortner CD, Sifre MI, et al A novel role for the T-box transcription factor *Tbx1* as a negative regulator of tumor cell growth in mice. Mol Carcinog. 2011; 50: 981–991. 10.1002/mc.20768 . 21438027 PMC3125489

[67] Tsukasaki K, Miller CW, Greenspun E, Eshaghian S, Kawabata H, Fujimoto T, et al Mutations in the mitotic check point gene, MAD1L1, in human cancers. Oncogene. 2001; 20: 3301–3305. . 11423979 10.1038/sj.onc.1204421

[68] Dolloff NG, Shulby SS, Nelson AV, Stearns ME, Johannes GJ, Thomas JD, et al Bone-metastatic potential of human prostate cancer cells correlates with Akt/PKB activation by alpha platelet-derived growth factor receptor. Oncogene. 2005; 24: 6848–6854. 10.1038/sj.onc.1208815 . 16007172 PMC2712354

[69] Ancrile B, Lim KH, Counter CM. Oncogenic Ras-induced secretion of IL6 is required for tumorigenesis. Genes Dev. 2007; 21: 1714–1719. 10.1101/gad.1549407 . 17639077 PMC1920165

[70] Mochizuki S, Okada Y. ADAMs in cancer cell proliferation and progression. Cancer Sci. 2007; 98: 621–628. 10.1111/j.1349-7006.2007.00434.x . 17355265 PMC11160018

[71] Kontos CK, Papadopoulos IN, Fragoulis EG, Scorilas1 A. Quantitative expression analysis and prognostic significance of L-DOPA decarboxylase in colorectal adenocarcinoma. Br J Cancer. 2010; 102: 1384–1390. 10.1038/sj.bjc.6605654 . 20424616 PMC2865762

[72] Satih S, Chalabi N, Rabiau N, Bosviel R, Fontana L, Bignon YJ, et al Gene expression profiling of breast cancer cell lines in response to soy isoflavones using a pangenomic microarray approach. OMICS. 2010; 14: 231–238. 10.1089/omi.2009.0124 . 20455703 PMC3128303

[73] Majidzadeh-A K, Esmaeili R, Abdoli N. *TFRC* and *ACTB* as the best reference genes to quantify Urokinase Plasminogen Activator in breast cancer. BMC Res Notes. 2011; 4: 215 10.1186/1756-0500-4-215 . 21702980 PMC3141519

[74] Liu HY, Dong Z. Gli3 silencing enhances cyclopamine suppressive effects on ovarian cancer. Onco Targets Ther. 2014; 7: 2007–2011. 10.2147/OTT.S57346 . 25378935 PMC4218910

[75] Nguyen DP, Li J, Tewari AK. Inflammation and prostate cancer: the role of interleukin 6 (IL-6). BJU Int. 2014; 113: 986–992. 10.1111/bju.12452 . 24053309

[76] Lv W, Wang L, Lu J, Mu J, Liu Y, Dong P. Long noncoding RNA KIAA0125 potentiates cell migration and invasion in gallbladder cancer. Biomed Res Int. 2015; 2015: 108458 10.1155/2015/108458 . 26448925 PMC4584029

[77] Zhang Y, Mao XY, Liu X, Song RR, Berney D, Lu YJ, et al High frequency of the *SDK1*: *AMACR* fusion transcript in Chinese prostate cancer. Int J Clin Exp Med. 2015; 8: 15127–15136. . 26628996 PMC4658885

[78] Sozzi G, Veronese ML, Negrini M, Baffa R, Cotticelli MG, Inoue H, et al The *FHIT* gene 3p14.2 is abnormal in lung cancer. Cell. 1996; 85: 17–26. 10.1016/S0092-8674(00)81078-8 . 8620533

[79] Vie N, Copois V, Bascoul-Mollevi C, Denis V, Bec N, Robert B, et al Overexpression of phosphoserine aminotransferase PSAT1 stimulates cell growth and increases chemoresistance of colon cancer cells. Mol Cancer. 2008; 7: 14 10.1186/1476-4598-7-14 . 18221502 PMC2245978

[80] Oshima H, Ishikawa T, Yoshida GJ, Naoi K, Maeda Y, Naka K, et al *TNF-α*/*TNFR1* signaling promotes gastric tumorigenesis through induction of *Noxo1* and *Gna14* in tumor cells. Oncogene. 2014; 33: 3820–3829. 10.1038/onc.2013.356 . 23975421

[81] Oba-Shinjo SM, Caballero OL, Jungbluth AA, Rosemberg S, Old LJ, Simpson AJ, et al Cancer-testis (CT) antigen expression in medulloblastoma. Cancer Immun. 2008; 8: 7 . 18426187 PMC2935780

[82] Kuroki T, Yendamuri S, Trapasso F, Matsuyama A, Aqeilan RI, Alder H, et al The tumor suppressor gene *WWOX* at *FRA16D* is involved in pancreatic carcinogenesis. Clin Cancer Res. 2004; 10: 2459–2465. 10.1158/1078-0432.CCR-03-0096 . 15073125

[83] Engehausen DG, Endele S, Krause SF, Rith T, Schrott KM, Akcetin Z. Polymorphisms in the human progesterone receptor (PGR) gene of two human prostate adenocarcinoma cell lines. Anticancer Res. 2005; 25: 1607–1609. . 16033068

[84] Matouk IJ, DeGroot N, Mezan S, Ayesh S, Abu-lail R, Hochberg A, et al The H19 non-coding RNA is essential for human tumor growth. PLOS ONE. 2007; 2: e845 10.1371/journal.pone.0000845 PMCID: PMC1959184. 17786216 PMC1959184

[85] Guilbert A, Dhennin-Duthille I, Hiani YE, Haren N, Khorsi H, Sevestre H, et al Expression of *TRPC6* channels in human epithelial breast cancer cells. BMC Cancer. 2008; 8: 125 10.1186/1471-2407-8-125 . 18452628 PMC2409351

[86] Veeriah S, Brennan C, Meng S, Singh B, Fagin JA, Solit DB, et al The tyrosine phosphatase PTPRD is a tumor suppressor that is frequently inactivated and mutated in glioblastoma and other human cancers. Proc Natl Acad Sci U S A. 2009; 106: 9435–9440. 10.1073/pnas.0900571106 . 19478061 PMC2687998

[87] Von Holstein SL, Fehr A, Heegaard S, Therkildsen MH, Stenman G. *CRTC1*-*MAML2* gene fusion in mucoepidermoid carcinoma of the lacrimal gland. Oncol Rep. 2012; 27: 1413–1416. 10.3892/or.2012.1676 . 22323114

[88] Feigenberg T, Gofrit ON, Pizov G, Hochberg A, Benshushan A. Expression of the H19 oncofetal gene in premalignant lesions of cervical cancer: a potential targeting approach for development of nonsurgical treatment of high-risk lesions. ISRN Obstet Gynecol. 2013; 2013: 137509 10.1155/2013/137509 . 23984081 PMC3747480

[89] Mo ML, Okamoto J, Chen Z, Hirata T, Mikami I, Bosco-Clément G, et al Down-regulation of SIX3 is associated with clinical outcome in lung adenocarcinoma. PLOS ONE. 2013; 8: e71816 10.1371/journal.pone.0071816 . 23977152 PMC3745425

[90] Yan L, Zhou J, Gao Y, Ghazal S, Lu L, Bellone S, et al Regulation of tumor cell migration and invasion by the H19/let-7 axis is antagonized by metformin-induced DNA methylation. Oncogene. 2015; 34: 3076–3084. 10.1038/onc.2014.236 . 25088204

[91] Ohana P, Kopf E, Bibi O, Ayesh S, Schneider T, Laster M, et al The expression of the H19 gene and its function in human bladder carcinoma cell lines. FEBS Lett. 1999; 454: 81–84. 10.1016/S0014-5793(99)00780-2 . 10413100

[92] Ohana P, Bibi O, Matouk I, Levy C, Birman T, Ariel I, et al Use of H19 regulatory sequences for targeted gene therapy in cancer. Int J Cancer. 2002; 98: 645–650. 10.1002/ijc.10243 . 11920631

[93] Matouk I, Ohana P, Ayesh S, Sidi A, Czerniak A, de Groot N, et al The Oncofetal H19 RNA in human cancer, from the bench to the patient. Cancer Therapy. 2005; 3: 249–266.

[94] Yang J, Mani SA, Donaher JL, Ramaswamy S, Itzykson RA, Come C, et al Twist, a master regulator of morphogenesis, plays an essential role in tumor metastasis. Cell. 2004; 117: 927–939. 10.1016/j.cell.2004.06.006 . 15210113

[95] Ostman A, Hellberg C, Böhmer FD. Protein-tyrosine phosphatases and cancer. Nat Rev Cancer. 2006; 6: 307–320. 10.1038/nrc1837 . 16557282

[96] Nakamura M, Kishi M, Sakaki T, Hashimoto H, Nakase H, Shimada K, et al Novel tumor suppressor loci on 6q22–23 in primary central nervous system lymphomas. Cancer Res. 2003; 63: 737–741. . 12591717

[97] Meehan M, Parthasarathi L, Moran N, Jefferies CA, Foley N, Lazzari E, et al Protein tyrosine phosphatase receptor delta acts as a neuroblastoma tumor suppressor by destabilizing the aurora kinase A oncogene. Mol Cancer. 2012; 11: 6 10.1186/1476-4598-11-6 . 22305495 PMC3395855

[98] Ortiz B, Fabius AW, Wu WH, Pedraza A, Brennan CW, Schultz N, et al Loss of the tyrosine phosphatase PTPRD leads to aberrant STAT3 activation and promotes gliomagenesis. Proc Natl Acad Sci U S A. 2014; 111: 8149–8154. 10.1073/pnas.1401952111 . 24843164 PMC4050622

[99] Schrock MS and Huebner K. *WWOX*: a fragile tumor suppressor. Exp Biol Med (Maywood). 2015; 240: 296–304. 25538133 10.1177/1535370214561590 PMC4471953

[100] Hezova R, Ehrmann J, Kolar Z. *WWOX*, a new potential tumor suppressor gene. Biomed Pap Med Fac Univ Palacky Olomouc Czech Repub. 2007; 151: 11–15. . 17690733 10.5507/bp.2007.002

[101] Yang J, Zhang W. WWOX tumor suppressor gene. Histol Histopathol. 2008; 23: 877–882. . 18437686 10.14670/HH-23.877

[102] Aldaz CM, Ferguson BW, Abba MC. *WWOX* at the crossroads of cancer, metabolic syndrome related traits and CNS pathologies. Biochim Biophys Acta. 2014; 1846: 188–200. 10.1016/j.bbcan.2014.06.001 . 24932569 PMC4151823

[103] Ekizoglu S, Bulut P, Karaman E, Kilic E, Buyru N. Epigenetic and genetic alterations affect the *WWOX* gene in head and neck squamous cell carcinoma. PLOS ONE. 2015; 10: e0115353 10.1371/journal.pone.0115353 . 25612104 PMC4303423

[104] Abu-Remaileh M, Joy-Dodson E, Schueler-Furman O, Aqeilan RI. Pleiotropic functions of tumor suppressor *WWOX* in normal and cancer cells. J Biol Chem. 2015; 290: 30728–30735. 10.1074/jbc.R115.676346 . 26499798 PMC4692203

[105] Yan H, Tong J, Lin X, Han Q, Huang H. Effect of the *WWOX* gene on the regulation of the cell cycle and apoptosis in human ovarian cancer stem cells. Mol Med Rep. 2015; 12: 1783–1788. 10.3892/mmr.2015.3640 . 25891642 PMC4464321

