# Supplementary figures and images for: Genomic Alteration in Head and Neck Squamous Cell Carcinoma (HNSCC) Cell Lines Inferred from Karyotyping, Molecular Cytogenetics, and Array Comparative Genomic Hybridization
Source: PLoS One. 2016 Aug 8;11(8):e0160901. doi: 10.1371/journal.pone.0160901 (PMC4976893; doi:10.1371/journal.pone.0160901)

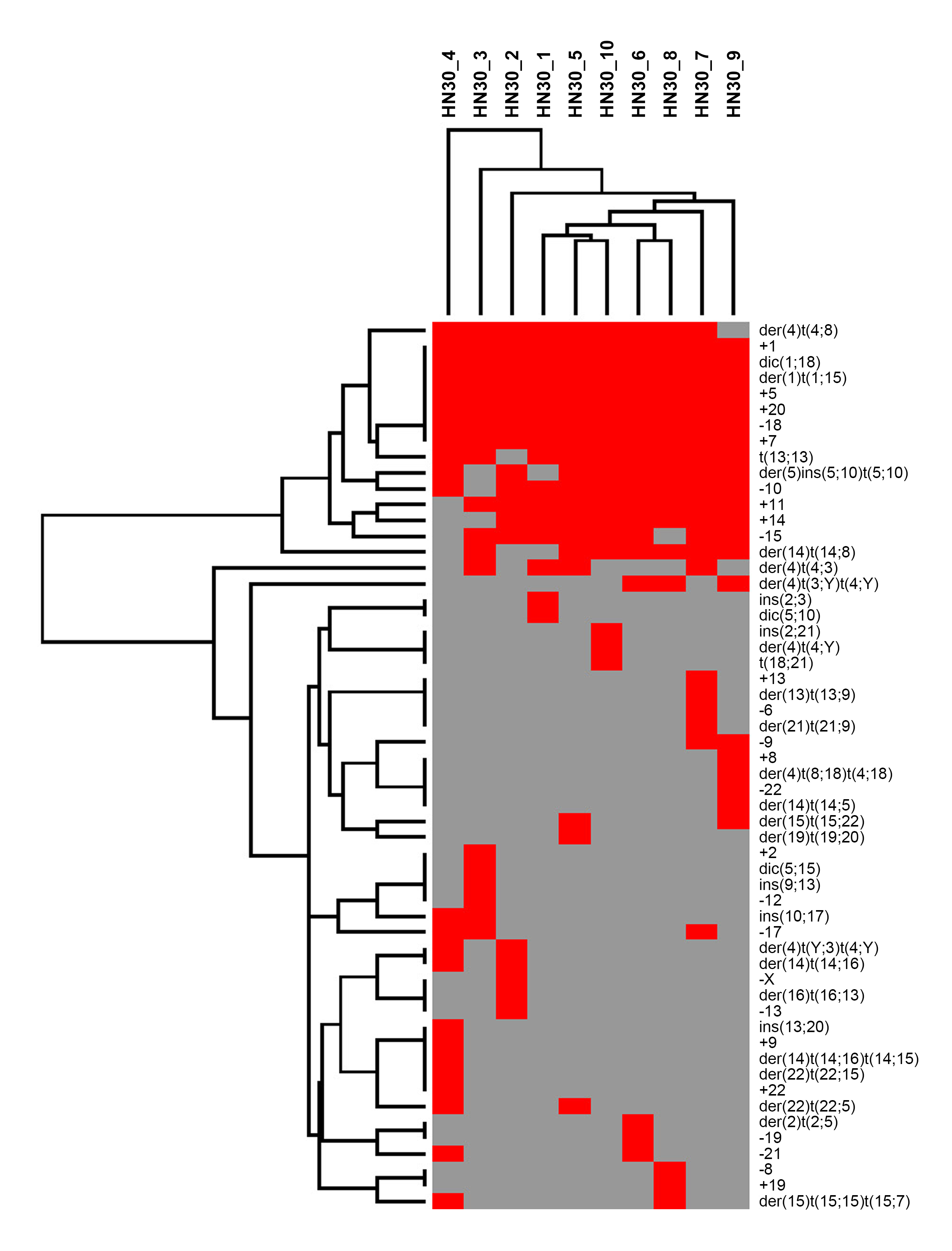

Supplement: S1 Fig — Each column refers to a metaphase and each row to type of a chromosomal abnormality. Red indicates the presence of each abnormality. Black indicates the absence of each abnormality. (TIF) [file pone.0160901.s001.tif]

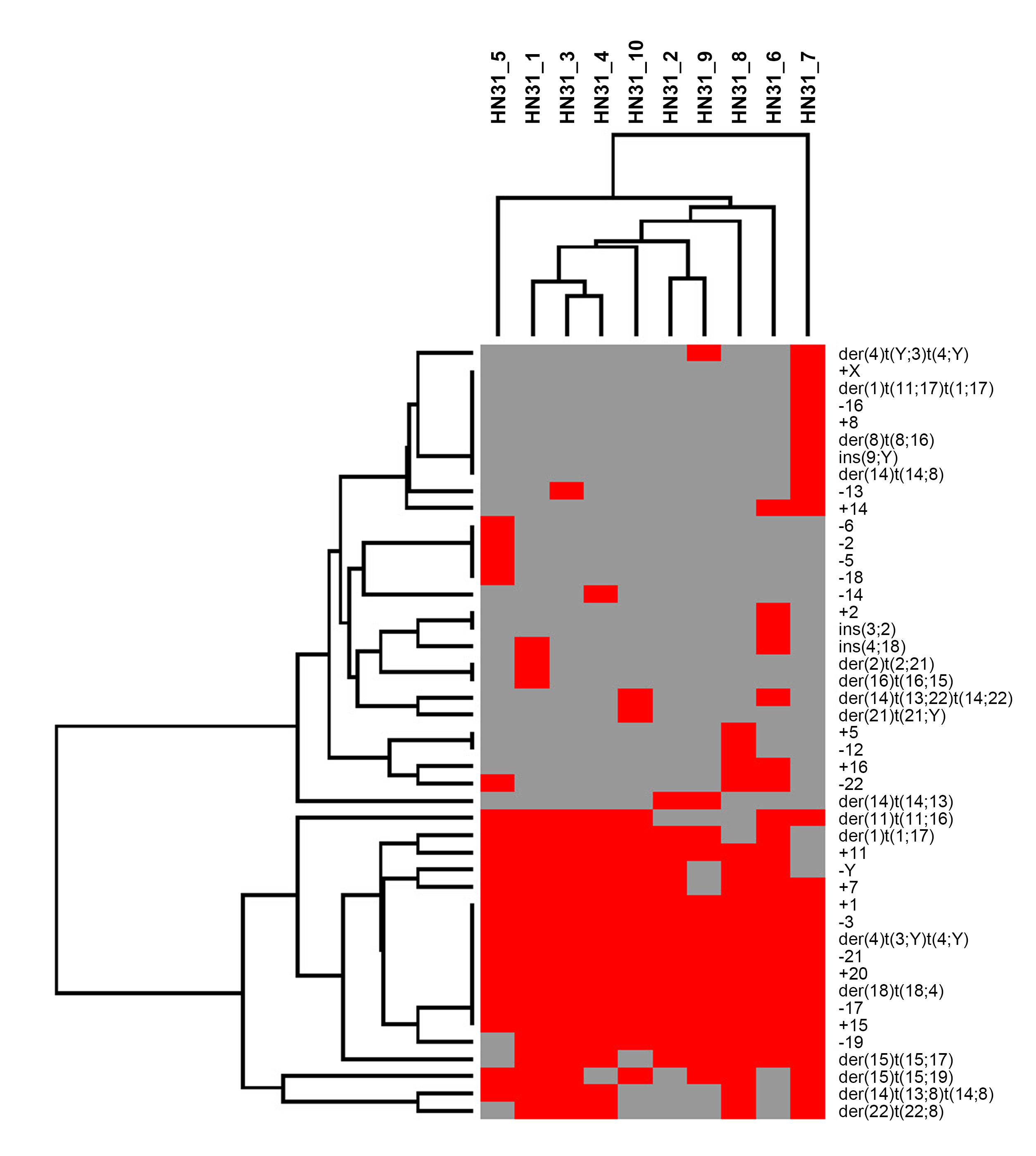

Supplement: S2 Fig — Each column refers to a metaphase and each row to type of a chromosomal abnormality. Red indicates the presence of each abnormality. Black indicates the absence of each abnormality. (TIF) [file pone.0160901.s002.tif]

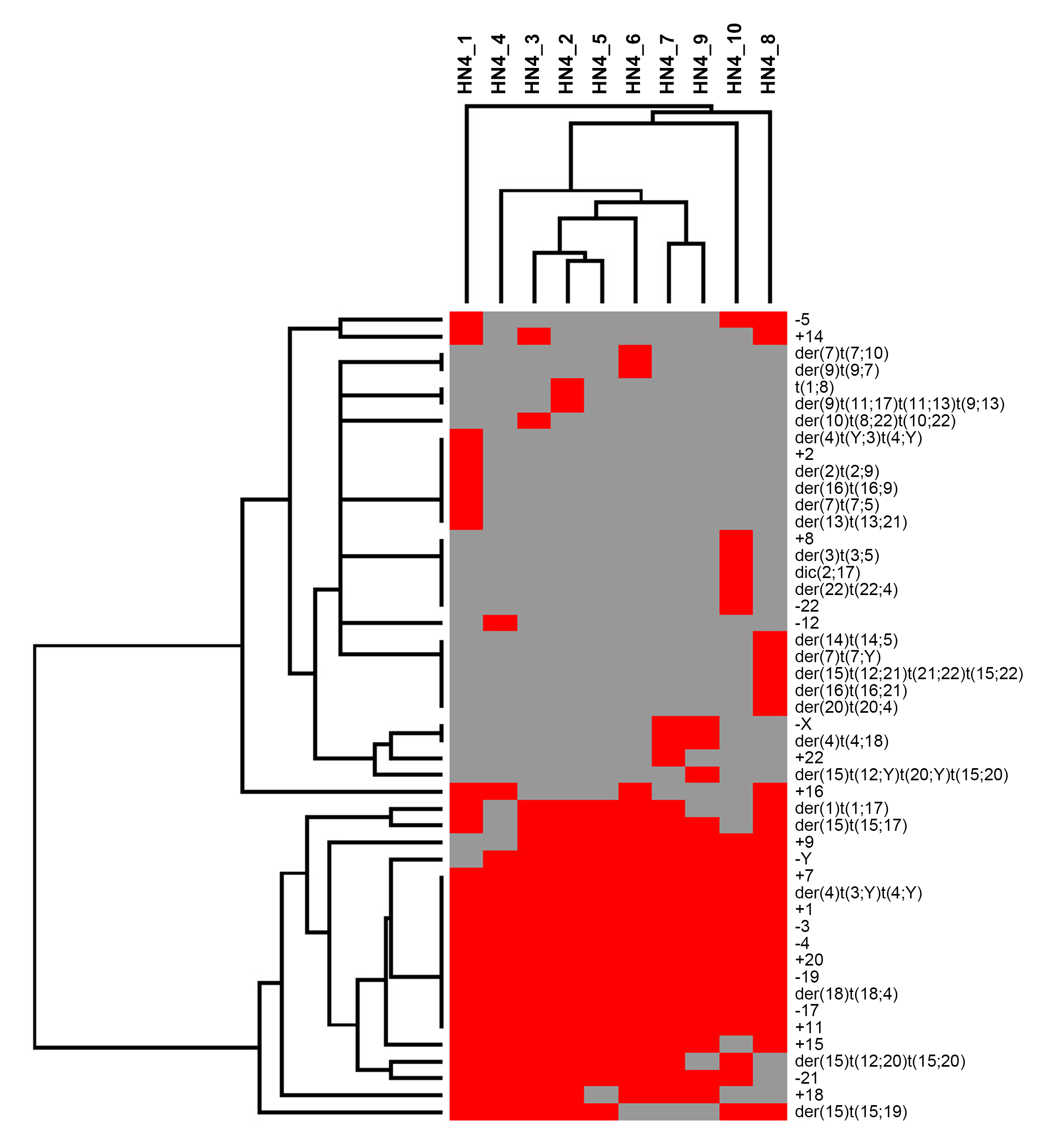

Supplement: S3 Fig — Each column refers to a metaphase and each row to type of a chromosomal abnormality. Red indicates the presence of each abnormality. Black indicates the absence of each abnormality. (TIF) [file pone.0160901.s003.tif]

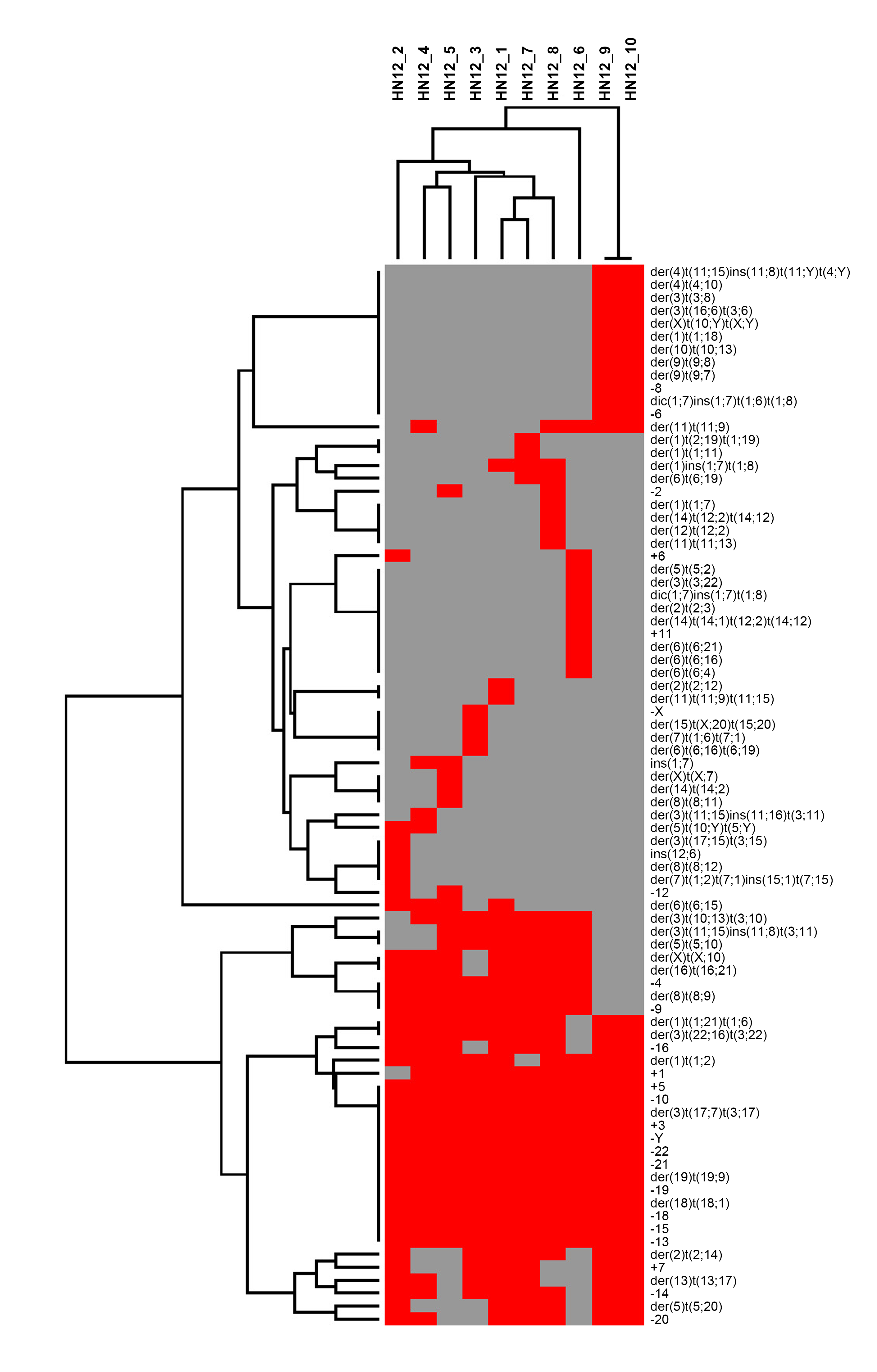

Supplement: S4 Fig — Each column refers to a metaphase and each row to type of a chromosomal abnormality. Red indicates the presence of each abnormality. Black indicates the absence of each abnormality. (TIF) [file pone.0160901.s004.tif]

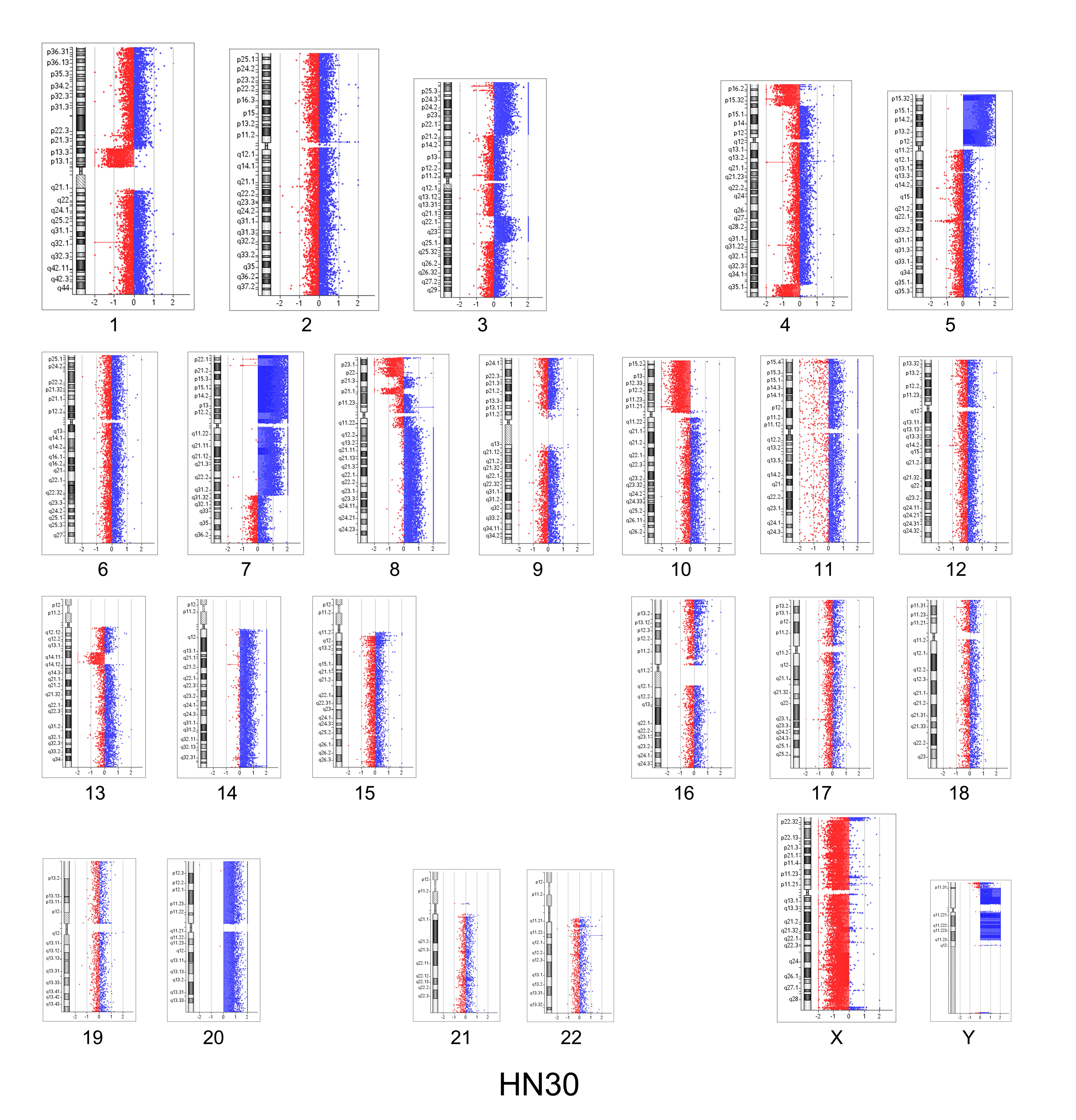

Supplement: S5 Fig — Detailed genomic profiles on chromosome, the X-axis represents the normalize log2 ratio fluorescence intensity thresholds -0.9 (loss) and 0.53 (gain), while the Y-axis represents the ideogram of human chromosome. (JPG) [file pone.0160901.s005.jpg]

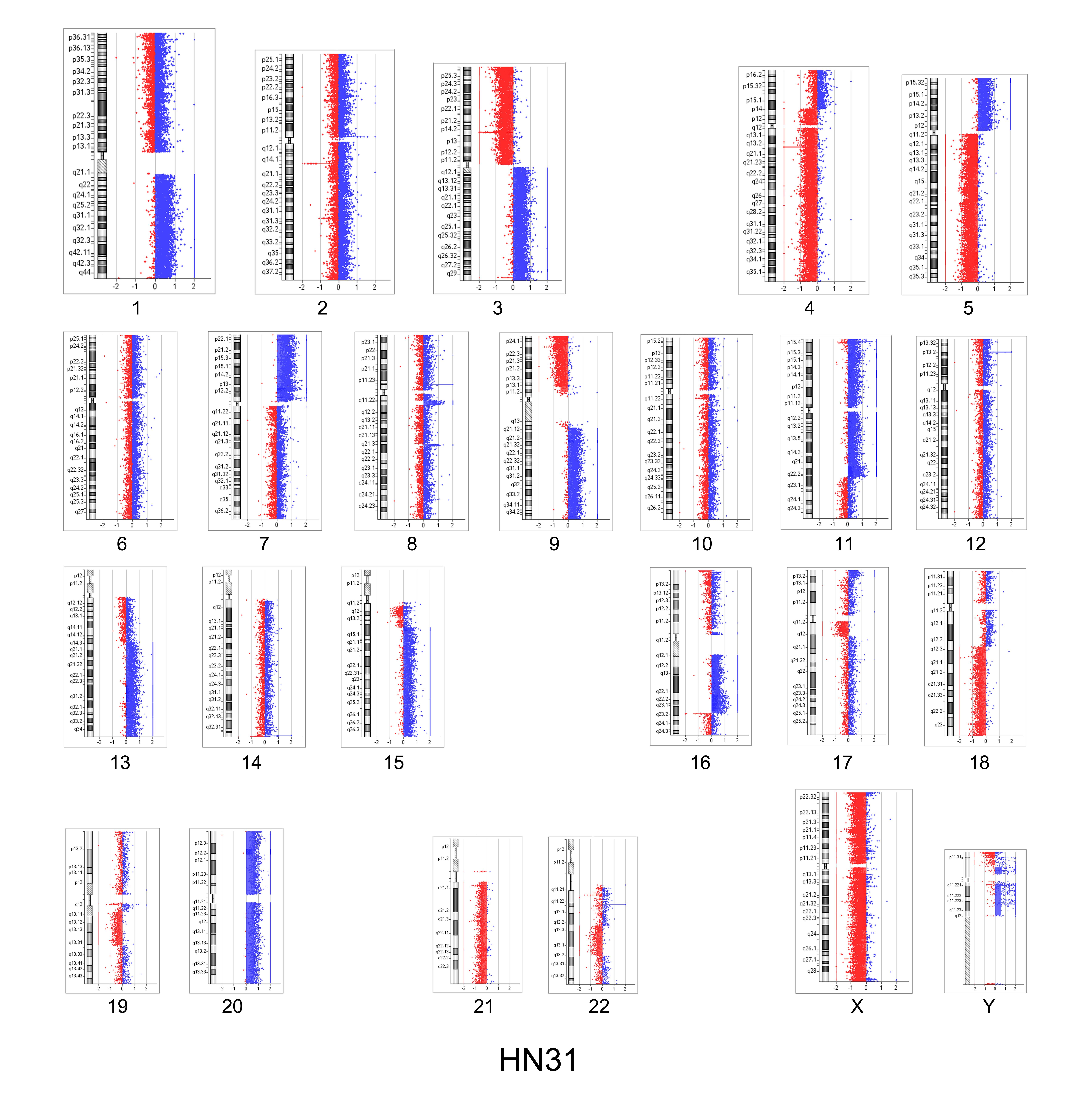

Supplement: S6 Fig — Detailed genomic profiles on chromosome, the X-axis represents the normalize log2 ratio fluorescence intensity thresholds -0.9 (loss) and 0.53 (gain), while the Y-axis represents the ideogram of human chromosome. (JPG) [file pone.0160901.s006.jpg]

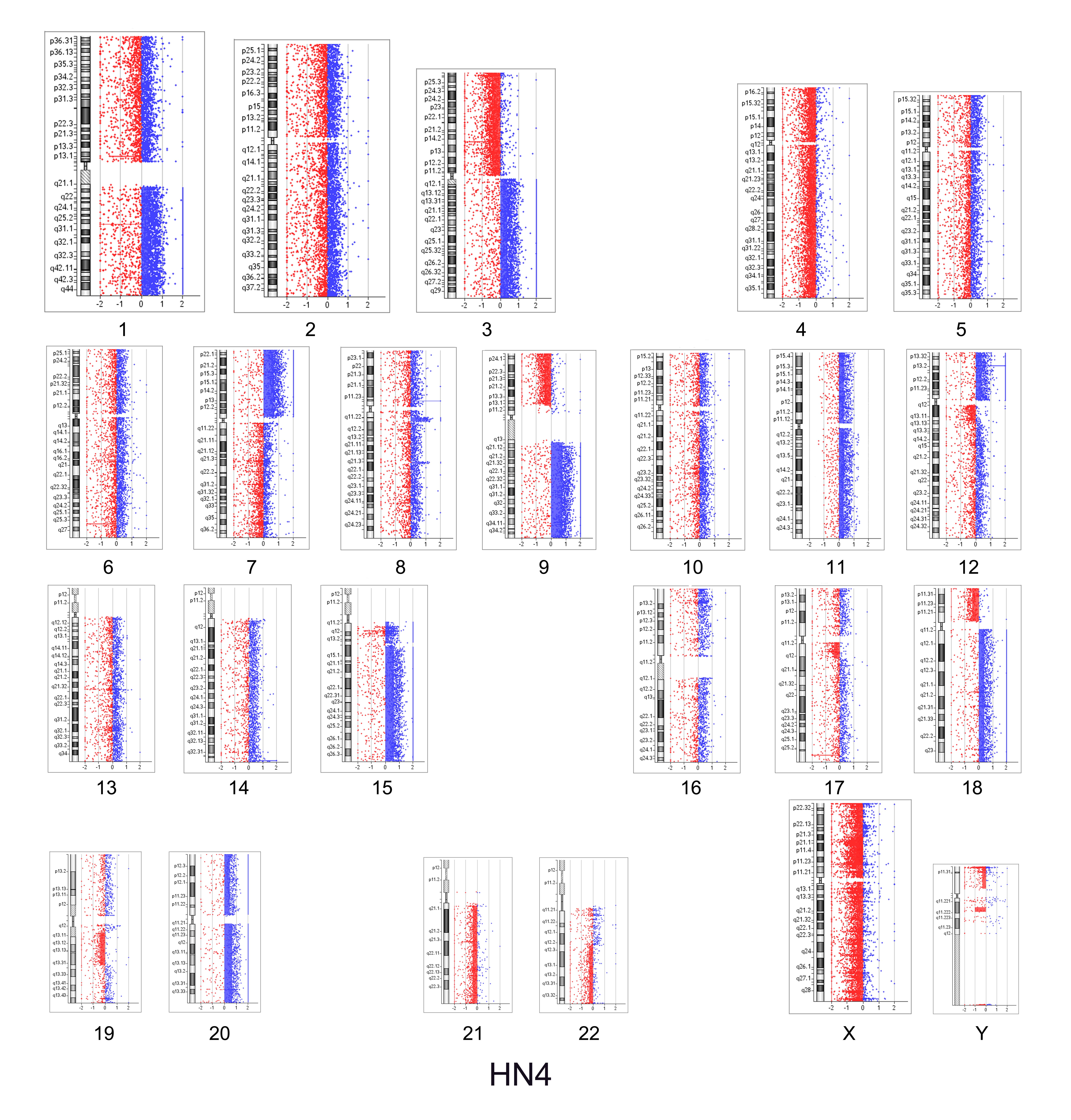

Supplement: S7 Fig — Detailed genomic profiles on chromosome, the X-axis represents the normalize log2 ratio fluorescence intensity thresholds -0.9 (loss) and 0.53 (gain), while the Y-axis represents the ideogram of human chromosome. (JPG) [file pone.0160901.s007.jpg]

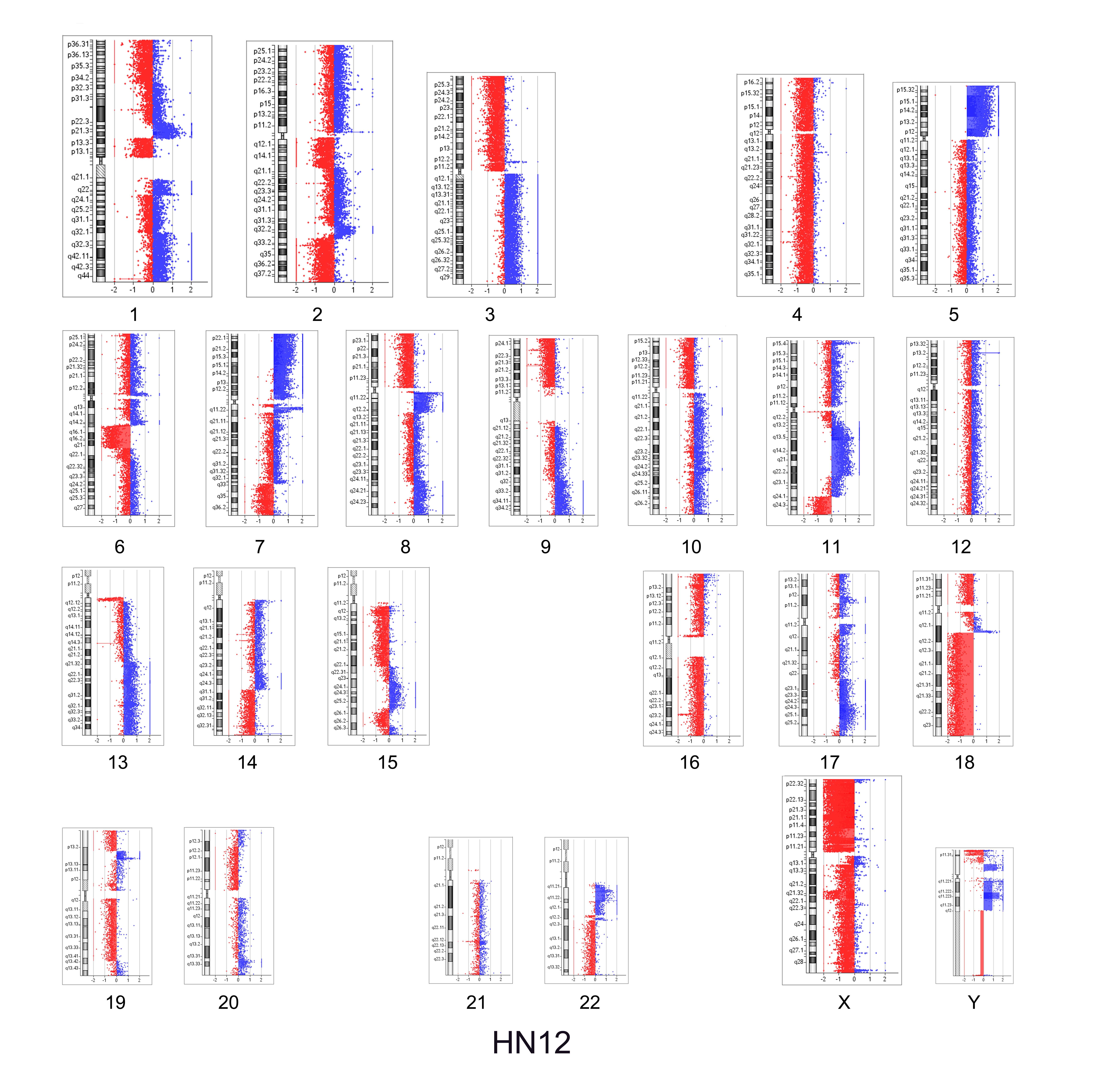

Supplement: S8 Fig — Detailed genomic profiles on chromosome, the X-axis represents the normalize log2 ratio fluorescence intensity thresholds -0.9 (loss) and 0.53 (gain), while the Y-axis represents the ideogram of human chromosome. (JPG) [file pone.0160901.s008.jpg]
