# Supplementary material for: Genomic Alteration in Head and Neck Squamous Cell Carcinoma (HNSCC) Cell Lines Inferred from Karyotyping, Molecular Cytogenetics, and Array Comparative Genomic Hybridization
Source: PLoS One. 2016 Aug 8;11(8):e0160901. doi: 10.1371/journal.pone.0160901 (PMC4976893; doi:10.1371/journal.pone.0160901)
Supplement: S1 Table — (DOCX) [file pone.0160901.s009.docx]

**S1 Table** Genome view of chromosome copy number variation (CNV).

| cell line | chromosome | chromosome banding | start–stop (bp) | size(kb) | amp / del | genes |
| --- | --- | --- | --- | --- | --- | --- |
|  |  |  |  |  |  |  |
| HN30 | 1 | p22.2^1^ | 89074748–89218821 | 144 | -0.96 | *PKN2* |
|  |  | p21.1–p11.2^1^ | 102541046–121330906 | 18,790 | -0.90 | *COL11A1, AMY2B, AMY2A, AMY1A, AMY1C,  AMY1B, PRMT6, NTNG1, SARS, CELSR2,ORT1, PSMA5, GPR61, GNAI3, GNAT2,  AMPD2, GSTM4, GSTM2, GSTM1, GSTM5,  GSTM3, CSF1, ALX3, UBL4B, KCNC4,  RBM15, HBXIP, PROK1, CYMP, KCNA10,  KCNA3,  CD53, CHIA, WDR77, ADORA3,  RAP1A, DDX20, KCND3, CAPZA1, MOV10,  PPM1J, SLC16A1, LRIG2, PHTF1, PTPN22,  AP4B1,  DCLRE1B, SYT6, TRIM33, BCAS2,  AMPD1, NRAS, CSDE1, SYCP1, TSHB,  VANGL1, CASQ2, NHLH2, SLC22A15, ATP1A1,  CD58,  IGSF3, CD2, PTGFRN, TTF2,  TRIM45, MAN1A2, WDR3, TBX15, WARS2,  HAO2, HSD3B2, HSD3B1, PHGDH, HMGCS2,  REG4,  ADAM30, NOTCH2, FCGR1B* |
|  |  | q31.3^1^ | 196744721–196799302 | 55 | -3.80 | *CFHR3, CFHR1* |
|  | 2 | p11.2 | 89141608–89301214 | 160 | 1.56 |  |
|  | 3 | p26.3–p21.31 | 117735–48924847 | 48,807 | 0.55 | *CHL1, CNTN6, CNTN4, IL5RA, TRNT1,  CRBN, SETMAR, SUMF1, ITPR1, GRM7,  LMCD1, CAV3, OXTR, RAD18, SRGAP3,  MTMR14, BRPF1, OGG1, CAMK1, IL17RC,  CRELD1, FANCD2, VHL, GHRL, ATP2B2,  SLC6A1, HRH1, SYN2, TIMP4, PPARG,  TSEN2,  MKRN2, RAF1, NUP210, FBLN2,  WNT7A, TMEM43, XPC, LSM3, SLC6A6,  NR2C2, MRPS25, CAPN7, SH3BP5, EAF1,  COLQ,  BTD, ANKRD28, DPH3, DAZL,  SATB1, RAB5A, SGOL1, UBE2E2, RPL15,  THRB, RARB, TOP2B, NGLY1, OXSM,  SLC4A7, EOMES, AZI2, RBMS3, TGFBR2,  OSBPL10, GPD1L, CCR4, GLB1, CRTAP, FBXL2, UBP1, CLASP2, PDCD6IP, MIR1282,  STAC, MLH1,  GOLGA4, ITGA9, PLCD1,  DLEC1, ACAA1, MYD88, XYLB,  ACVR2B,  SCN5A, SCN10A, SCN11A, WDR48, GORASP1,  CX3CR1, CCR8, SLC25A38, MOBP, MYRIP,  ENTPD3, CTNNB1, TRAK1,  CCK, LYZL4,  VIPR1, SS18L2, NKTR, CCBP2, CYP8B1,  SNRK,  ABHD5, ZNF35, TGM4, CDCP1,  LARS2, LIMD1, SACM1L, SLC6A20, LZTFL1,  CCR9, CXCR6, XCR1, CCR1, CCR3,  CCR2, CCR5,  CCRL2, LTF, RTP3,  LRRC2, TDGF1, ALS2CL, TMIE, MYL3,   SETD2, PTPN23, SCAP, SMARCC1, MAP4,  CDC25A, CAMP, NME6, FBXW12, ATRIP,  TREX1, PFKFB4, UCN2, COL7A1, UQCRC1, SLC26A6, CELSR3, NCKIPSD, IP6K2, PRKAR2A, SLC25A20* |
|  |  | p26.2 | 3529292–3587832 | 59 | -1.11 |  |
|  |  | p26.1 | 7442823–7657894 | 215 | -0.90 | *GRM7* |
|  |  | p12.1^1^ | 85615568–85870596 | 255 | -0.90 | *CADM2* |
|  |  | q21.1–q24 | 123520161–146985724 | 23466 | 0.56 | *MYLK, ROPN1, UMPS, MUC13, ZNF148,  SNX4, OSBPL11, SLC41A3, KLF15, UROC1,  TXNRD3, PLXNA1, MCM2, ABTB1, MGLL,   RUVBL1, GATA2, RPN1, ACAD9, GP9,  ISY1, MBD4, RHO,  PLXND1, TRH,  ATP2C1, NEK11, ACPP, CCRL1, UBA5,  NPHP3, BFSP2, TOPBP1, TF, KY,  EPHB1, PCCB, SOX14, MRAS, PIK3CB, FOXL2, MRPS22, COPB2, RBP2, RBP1,  NMNAT3, SPSB4, ZBTB38, RASA2, RNF7,  GRK7, ATP1B3, TFDP2, XRN1, ATR,  PLS1,  TRPC1, PCOLCE2, CHST2, SLC9A9,  C3orf58, PLOD2, PLSCR4,  PLSCR2, PLSCR1* |
|  | 4 | p15.33 | 13106682–13360120 | 253 | -4.33 | *HSP90AB2P* |
|  |  | q13.2 | 69392576–69483277 | 91 | 1.12 | *UGT2B17, UGT2B15* |
|  |  | q13.2 | 70148989–70230159 | 81 | -2.31 | *UGT2B28* |
|  |  | q31.21 | 144842588–144905281 | 63 | -1.30 |  |
|  |  | q34.3–q35.2^1^ | 180247704–190469337 | 10222 | -0.90 | *DCTD, ING2, IRF2, CASP3, MLF1IP,  ACSL1, SLC25A4, TLR3,  CYP4V2, KLKB1,  F11, MTNR1A, FAT1* |
|  | 5 | p15.33–p11^1^ | 22149–46365277 | 46,343 | 1.28 | *SDHA, AHRR, SLC9A3, TPPP, SLC12A7,  SLC6A19, SLC6A18,  TERT, CLPTM1L, SLC6A3,  LPCAT1, MRPL36, NDUFS6, IRX4,  IRX2,  IRX1, ADAMTS16, MED10, NSUN2, SRD5A1,  ADCY2,  MTRR, CCT5, ROPN1L, DAP,  CTNND2, DNAH5, TRIO, ANKH,  FBXL7,  MYO10, BASP1, CDH18, CDH12, PMCHL1,  CDH10,  CDH9, CDH6, DROSHA, PDZD2,  GOLPH3, MTMR12, NPR3,  TARS, ADAMTS12,  SLC45A2, AMACR, RAI14, RAD1, AGXT2,   PRLR, IL7R, SKP2, SLC1A3, NIPBL,  NUP155, GDNF, LIFR, OSMR, C9,  DAB2, PTGER4, PRKAA1, RPL37, CARD6,  C7, C6, OXCT1, FBXO4, GHR,  SEPP1, ZNF131, HMGCS1, CCL28, PAIP1,  NNT,  FGF10, MRPS30* |
|  |  | q14.3^1^ | 83617152–83816674 | 200 | -0.99 | *EDIL3* |
|  |  | q22.2–q22.3^1^ | 112480515–114095601 | 1615 | -0.91 | *MCC, KCNN2* |
|  | 7 | p22.3–p11.2^1^ | 54185–57923933 | 57870 | 1.46 | *FAM20C, PDGFA, PRKAR1B, SUN1, ZFAND2A,  MAFK, MAD1L1, FTSJ2, NUDT1, EIF3B,  LFNG, GNA12, CARD11, RADIL,  PAPOLB,  ACTB, FSCN1, RNF216, OCM, PMS2,  CYTH3, ZNF12, C1GALT1, COL28A1, RPA3,  ICA1, THSD7A, ETV1, DGKB,  MEOX2,  SOSTDC1, AGR2, AGR3, AHR, SNX13,  PRPS1L1,  HDAC9, TWISTNB, MACC1, ABCB5,  SP8, SP4, DNAH11,  CDCA7L, IL6,  FAM126A, KLHL7, IGF2BP3, STK31, NPY,  MPP6, DFNA5, OSBPL3, CYCS, NFE2L3,  HOXA1, HOXA2, HOXA3,  HOXA4, HOXA5,  HOXA6, HOXA7, HOXA9, MIR196B, HOXA10,  HOXA11, HOXA13, EVX1, TAX1BP1, JAZF1,  CPVL, CHN2, WIPF3, ZNRF2, NOD1,  GGCT, GARS, CRHR2, INMT, AQP1,  GHRHR,  ADCYAP1R1, NEUROD6, LSM5, AVL9,  NT5C3, RP9, BBS9, AAA1, NPSR1,  TBX20, SEPT7, AOAH, ELMO1, TXNDC3,  SFRP4,  STARD3NL, AMPH, VPS41, POU6F2,  RALA, C7orf11, C7orf10,  INHBA, GLI3,  PSMA2, MRPL32, BLVRA, MRPS24, PGAM2,   POLD2, GCK, CAMK2B, NPC1L1, TMED4,  OGDH, PPIA, CCM2, RAMP3, ADCY1,  IGFBP1, IGFBP3, TNS3, PKD1L1, HUS1,  UPP1, ABCA13, VWC2, IKZF1, DDC,  GRB10, HPVC1, EGFR, LANCL2,  SEPT14,  MRPS17, GBAS, PHKG1* |
|  |  | p22.2 | 3384223–3470044 | 86 | -0.90 | *SDK1* |
|  |  | p21.3 | 8751128–8971052 | 220 | -1.16 | *NXPH1* |
|  |  | q11.1–q31.31^1^ | 61059509–118972081 | 57913 | 0.96 | *ZNF107, ZNF138, ZNF117, ERV3, ZNF92,  GUSB, ASL, TPST1,  KCTD7, RABGEF1,  SBDS, TRIM50, FKBP6, FZD9, BAZ1B,   BCL7B, TBL2, MLXIPL, VPS37D, STX1A,  WBSCR26, CLDN3,  CLDN4, WBSCR27, WBSCR28,  ELN, LIMK1, EIF4H, LAT2, RFC2, CLIP2, GTF2IRD1, GTF2I, NCF1, TRIM74,  TRIM73, HIP1, CCL26, CCL24, POR,  MDH2, HSPB1, YWHAG, SRCRB4D, ZP3,  UPK3B, POMZP3, FGL2, PTPN12, GNAI1,  CD36, HGF, PCLO, SEMA3E, GRM3,  CROT, ABCB4, ABCB1, SLC25A40, DBF4,  ADAM22, SRI, STEAP4, STEAP2, GTPBP10,  FZD1, MTERF, AKAP9, CYP51A1,  KRIT1,  PEX1, CDK6, SAMD9, SAMD9L, CALCR,  TFPI2, GNGT1, COL1A2, CASD1, SGCE,  PEG10, PPP1R9A, PON1, PON3, PON2,  ASB4, PDK4, SLC25A13, SHFM1, DLX6,  DLX5, TAC1, ASNS,  LMTK2, BRI3,  BAIAP2L1, NPTX2, TRRAP, SMURF1, MYH16,   PDAP1, CPSF4, CYP3A5, CYP3A7, CYP3A4,  CYP3A43, GJC3,  AZGP1, ZNF3, MCM7,  MIR25, MIR93, MIR106B, AP4M1, TAF6,  CNPY4, GAL3ST4, STAG3, PILRB, PILRA,  TSC22D4, FBXO24,  PCOLCE, TFR2, ACTL6B,  GNB2, GIGYF1, POP7, EPO, ZAN,   EPHB4, TRIP6, ACHE, MUC12, MUC17,  SERPINE1, AP1S1, VGF, PLOD3, FIS1,  ORAI2, POLR2J, POLR2J2, ARMC10, NAPEPLD,  DNAJC2, PSMC2, SLC26A5, RELN, LHFPL3,  MLL5, NAMPT,  PIK3CG, PRKAR2B, COG5,  GPR22, SLC26A4, CBLL1, SLC26A3, DLD,  LAMB1, NRCAM, PNPLA8, THAP5, DNAJB9,  IMMP2L,  DOCK4, ZNF277, IFRD1, GPR85,  PPP1R3A, FOXP2, TES, CAV2, CAV1,  MET, CAPZA2, ST7, WNT2, ASZ1,  CFTR, CTTNBP2,  ANKRD7* |
|  | 8 | p23.3–p22 | 686538–15306009 | 14619 | -0.90 | *DLGAP2, CLN8, ARHGEF10, MYOM2, CSMD1, MCPH1, ANGPT2, DEFB1, DEFA6, DEFA4, DEFA1, DEFA5, FAM90A14, FAM90A13, FAM90A5, FAM90A20, DEFB103A, FAM90A7, FAM90A19,  FAM90A18,  FAM90A8, FAM90A9, FAM90A10, MFHAS1, TNKS,  MIR1241, RP1L1, SOX7, PINX1, MTMR9,  FAM167A, BLK, GATA4, FDFT1, CTSB,  DLC1* |
|  |  | p23.1 | 12241093–12467543 | 226 | 0.64 | *FAM66A, DEFB109P1, FAM90A25P, FAM86B2* |
|  |  | p22 | 15952011–16241027 | 289 | 0.63 | *MSR1* |
|  |  | p21.2–p12 | 24193014–29000762 | 4808 | -0.91 | *ADAM28, ADAMDEC1, ADAM7, NEFM, NEFL,  GNRH1, EBF2,  BNIP3L, DPYSL2, ADRA1A,  PTK2B, CHRNA2, EPHX2, CLU,  SCARA3,  ESCO2, ELP3, PNOC, FBXO16, FZD3,  EXTL3, KIF13B* |
|  |  | p11.22 | 39258894–39381514 | 123 | 4.79 | *ADAM5P, ADAM3A* |
|  |  | q11.23–q24.3^1^ | 55445546–146280020 | 90834 | 0.57 | *RP1, TGS1, LYN, RPS20, MOS,  PLAG1, CHCHD7, PENK, CYP7A1, NSMAF,  TOX, CA8, RAB2A, CHD7, ASPH,  NKAIN3, GGH, TTPA, CYP7B1, PDE7A,  CRH, ADHFE1, MYBL1, SGK3, SNHG6,   SNORD87, CSPP1, ARFGEF1, PREX2, SULF1,  PRDM14, EYA1,  TRPA1, KCNB2, TERF1,  RPL7, RDH10, STAU2, TCEB1, TMEM70,  JPH1, GDAP1, HNF4G, ZFHX4, PEX2,  PKIA, IL7, STMN2,  MRPS28, FABP5,  PMP2, FABP4, IMPA1, CA1, CA3,  CA2, WWP1, FAM82B, CPNE3, CNGB3,  MMP16, DECR1, CALB1, TMEM55A, OTUD6B,  TMEM67, PDP1, CDH17, GEM, RAD54B,  ESRP1,  C8orf38, GDF6, UQCRB, PTDSS1,  SDC2, MTDH, MATN2, RPL30, HRSP12,  KCNS2, OSR2, COX6C, FBXO43, POLR2K,  SPAG1,  PABPC1, YWHAZ, GRHL2, NCALD,  RRM2B, ODF1, AZIN1,  ATP6V1C1, BAALC,  FZD6, CTHRC1, SLC25A32, RIMS2, TM7SF4,  DPYS, ZFPM2, OXR1, ABRA, ANGPT1,  EIF3E, TRHR, PKHD1L1, EBAG9, SYBU,  KCNV1, CSMD3, TRPS1, EIF3H, RAD21,   SLC30A8, EXT1, TNFRSF11B, COLEC10, NOV,  ENPP2, DEPTOR, COL14A1, MTBP, SNTB1,  HAS2, ZHX1, ATAD2, FBXO32,  RNF139,  NDUFB9, MTSS1, SQLE, KIAA0196, FAM84B,  MYC,  PVT1, ASAP1, ADCY8, EFR3A,  OC90, HHLA1, KCNQ3, TG, SLA,  WISP1, NDRG1, KHDRBS3, COL22A1, KCNK9,  EIF2C2, PTK2,  GPR20, PTP4A3, BAI1,  ARC, JRK, PSCA, SLURP1, LYNX1,  LY6D, GML, CYP11B1, CYP11B2, LY6E,  LY6H, GPIHBP1, GLI4,  TOP1MT, MAFA,  NAPRT1, EEF1D, TSTA3, FAM83H, SCRIB,   GRINA, SPATC1, GPAA1, CYC1, SHARPIN,  SCXA, BOP1, HSF1,  DGAT1, SCRT1,  GPR172A, SLC39A4, VPS28, GPT, RECQL4,   ZNF34, RPL8, ZNF7, COMMD5, ZNF16* |
|  | 10 | p15.3–p11.1 | 102539–38649695 | 38547 | -0.92 | *DIP2C, ADARB2, PFKP, KLF6, AKR1C1,  AKR1C2, AKR1C3,  AKR1C4, NET1, CALML3,  GDI2, FBXO18, IL15RA, IL2RA, RBM17,  PFKFB3, PRKCQ, ITIH5, ITIH2, KIN,  ATP5C1, TAF3, GATA3,  USP6NL, UPF2,  OPTN, MCM10, PHYH, PRPF18, HSPA14,   SUV39H2, DCLRE1C, RPP38, NMT2, PTER,  RSU1, CUBN,  TRDMT1, VIM, PTPLA,  STAM, MRC1, CACNB2, PLXDC2, NEBL,  BMI1, SPAG6, PTF1A, OTUD1, ARHGAP21,  PRTFDC1, MYO3A, GAD2, PDSS1, ABI1,  ANKRD26, YME1L1, PTCHD3, RAB18, MKX, MPP7, BAMBI, SVIL, MAP3K8, LYZL2,  ZEB1, KIF5B, EPC1,  ITGB1, NRP1,  PARD3, CUL2, CREM, CCNY, GJD4,  FZD8,  ANKRD30A, ZNF25, ZNF33A* |
|  | 12 | q14.1 | 62636682–62918916 | 282 | -1.01 | *USP15, MON2* |
|  | 14 | q11.2–q32.33^1^ | 19265142–107287505 | 88022 | 0.53 | *PARP2, TEP1, OSGEP, ANG, RNASE4,  RNASE6, RNASE1,  RNASE3, RNASE2, SLC39A2,  NDRG2, RNASE7, RNASE8, ZNF219, RPGRIP1,  CHD8, RAB2B, METTL3, SALL2, DAD1,  OXA1L,*  *SLC7A7, MRPL52, MMP14, LRP10,  PSMB5, PSMB11, CEBPE,*  *SLC7A8, BCL2L2,  PABPN1, EFS, IL25, MYH6, MYH7,  NGDN,*  *THTPA, DHRS4, CPNE6, NRL,  PCK2, PSME1, PSME2, RNF31,*  *REC8,  TSSK4, CHMP4A, GMPR2, TINF2, TGM1,  RABGGTA,*  *LTB4R2, LTB4R, ADCY4, RIPK3,  NFATC4, CBLN3, CMA1, CTSG, GZMH,  GZMB, NOVA1, PRKD1, COCH, AP4S1,  ARHGAP5,*  *NPAS3, EGLN3, SNX6, CFL2,  BAZ1A, SRP54, KIAA0391, PSMA6, NFKBIA,  MBIP, NKX21, NKX28, PAX9, SLC25A21,  MIPOL1,  FOXA1, SSTR1, SEC23A, SIP1,  PNN, FBXO33, LRFN5, FSCB,  FANCM,  MDGA2, RPS29, RPL36AL, MGAT2, C14orf104,  POLE2, SOS2, L2HGDH, MAP4K5, PYGL,  TRIM9, GNG2, C14orf166,  NID2, PTGDR,  PTGER2, PSMC6, FERMT2, BMP4, CDKN3,   SAMD4A, GCH1, SOCS4, LGALS3, FBXO34,  KTN1, OTX2, PSMA3, ARID4A, TIMM9,  DACT1, DAAM1, RTN1, DHRS7, SIX6,  SIX1,  SIX4, MNAT1, TRMT5, TMEM30B,  PRKCH, HIF1A, SYT16,  KCNH5, PPP2R5E,  SGPP1, SYNE2, ESR2, HSPA2, SPTB,  GPX2,  FNTB, MAX, FUT8, MPP5,  PIGH, ARG2, RDH11, RDH12,  ZFYVE26,  RAD51L1, ZFP36L1, ACTN1, ERH, SLC10A1,  SMOC1, SYNJ2BP, ADAM21, ADAM20, MAP3K9,  RGS6, DPF3, RBM25,  PSEN1, NUMB,  C14orf169, ACOT2, DNAL1, ENTPD5, ALDH6A1,  ABCD4, TMEM90A, NPC2, LTBP2, DLST,  PGF, EIF2B2, MLH3,  ACYP1, NEK9,  TMED10, FOS, BATF, FLVCR2, C14orf1,  TTLL5,  TGFB3, ESRRB, NGB, POMT2,  GSTZ1, ISM2, SPTLC2, ALKBH1, NRXN3,  DIO2, TSHR, SEL1L, FLRT2, GALC,  GPR65, KCNK10,  SPATA7, TTC8, FOXN3,  TDP1, KCNK13, PSMC1, CALM1,  RPS6KA5,  GPR68, FBLN5, TRIP11, ATXN3, LGMN,  GOLGA5,  CHGA, BTBD7, ASB2, OTUB2,  DDX24, IFI27, SERPINA10,  SERPINA6, SERPINA1,  SERPINA4, SERPINA5, SERPINA3, GSC, DICER1,  CLMN, C14orf49, GLRX5, TCL6, TCL1B,  TCL1A,  BDKRB2, BDKRB1, PAPOLA, VRK1,  BCL11B, CCNK, CYP46A1,  EML1, DEGS2,  YY1, WARS, DLK1, MEG3, RTL1,  MIR431,  MIR433, MIR127, MIR136, MIR376B,  MIR134, DIO3OS, DIO3,  PPP2R5C, DYNC1H1,  HSP90AA1, RAGE, AMN, TNFAIP2, MARK3,  CKB, KLC1, XRCC3, C14orf2, MIR203,  INF2, AKT1, CDCA4, GPR132, JAG2,  BRF1, MTA1, CRIP2, CRIP1* |
|  | 15 | q11.1–q11.2 | 20481702–22698581 | 2217 | 0.75 | *BCL8, HERC2P3, GOLGA6L6, GOLGA8C, POTEB,  NF1P1* |
|  | 17 | q23.1 | 57751694–57999267 | 248 | -0.90 | *CLTC, PTRH2, VMP1, MIR21, TUBD1* |
|  | 18 | q23 | 77992253–78012829 | 21 | -0.93 | *PARD6G* |
|  | 20 | p13–p11.1^1^ | 67778–26312663 | 26245 | 0.94 | *SOX12, NRSN2, RBCK1, TBC1D20, CSNK2A1,  TCF15, ANGPT4, RSPO4, SNPH, FKBP1A,  SIRPB2, SIRPG, PDYN, STK35, TGM3,  TGM6, TMC2, IDH3B, EBF4, PTPRA,  GNRH2, MRPS26, OXT, AVP, ITPA,  SLC4A11, ATRN, ADAM33, HSPA12B, SPEF1,  CENPB, CDC25B, MAVS, PANK2, RNF24,  ADRA1D, PRNP, PRND, SLC23A2, C20orf30,  PCNA, CDS2, PROKR2, CHGB, MCM8,  BMP2, HAO1, PLCB4, SNAP25, MKKS,  JAG1, SPTLC3, TASP1, C20orf7, MACROD2,  FLRT3, OTOR, PCSK2, BFSP1, DSTN,  RRBP1, SNX5, ZNF133, RBBP9, SEC23B,  DTD1, SLC24A3, RIN2, CRNKL1, INSM1,  XRN2, NKX2–4, NKX2–2, PAX1, FOXA2,  SSTR4, THBD, NXT1, NAPB, CST11,  CST3, CST4, CST1, CST2, CST5,  GGTLC1, VSX1, ENTPD6, PYGB, GINS1,  NANP, PLCB1* |
|  |  | q11.21–q13.33 | 29462044–62949149 | 33487 | 0.91 | *DEFB118, HM13, ID1, COX4I2, MYLK2,  PDRG1, HCK, KIF3B, ASXL1, DNMT3B,  MAPRE1, BASE, PLUNC, CDK5RAP1, CBFA2T2,  NECAB3, E2F1, CHMP4B, ASIP, AHCY,  ITCH, DYNLRB1, PIGU, NCOA6, GGT7,  ACSS2, GSS, MYH7B, TRPC4AP, PROCR,  EIF6, UQCC, GDF5, CEP250, SPAG4,  NFS1, MYL9, TGIF2, SLA2, NDRG3,  SAMHD1, RBL1, RPN2, GHRH, SRC,  NNAT, CTNNBL1, TGM2, BPI, LBP,  MAFB, TOP1, PLCG1, ZHX3, LPIN3,  SGK2, MYBL2, JPH2, HNF4A, SERINC3,  PKIG, ADA, WISP2, KCNK15, RIMS4,  YWHAB, STK4, WFDC5, WFDC12, PI3,  SEMG1, SEMG2, MATN4, SDC4, SYS1,  DBNDD2, UBE2C, TNNC2, ACOT8, CTSA,  PLTP, ZNF335, MMP9, SLC12A5, CD40,  CDH22, ELMO2, SLC13A3, SLC2A10, EYA2,  NCOA3, SULF2, PREX1, ARFGEF2, CSE1L,  KCNB1, PTGIS, B4GALT5, SLC9A8, SPATA2,  RNF114, SNAI1, UBE2V1, CEBPB, PTPN1,  PARD6B, BCAS4, DPM1, KCNG1, NFATC2,  SALL4, ZNF217, BCAS1, CYP24A1, PFDN4,  DOK5, MC3R, AURKA, TFAP2C, BMP7,  SPO11, RBM38, CTCFL, PCK1, ZBP1,  PMEPA1, RAB22A, VAPB, STX16, MIR296,  GNAS, TH1L, CTSZ, TUBB1, ATP5E,  EDN3, SYCP2, PPP1R3D, CDH4, TAF4,  PSMA7, SS18L1, GTPBP5, OSBPL2, LAMA5,  RPS21, GATA5, SLCO4A1, NTSR1, C20orf20,  COL9A3, C20orf11, SLC17A9, HAR1B, HAR1A,  BIRC7, NKAIN4, ARFGAP1, CHRNA4, KCNQ2,  EEF1A2, GMEB2, STMN3, RTEL1, TNFRSF6B,  LIME1, TPD52L2, UCKL1, SOX18* |
|  | 22 | q11.21 | 19747165–19747992 | 1 | 1.10 | *TBX1* |
|  |  | q11.22 | 23056562–23228483 | 172 | 3.76 | *MIR650* |
| HN31 | 1 | p36.31^1^ | 6999378–7084687 | 85 | 0.89 | *CAMTA1* |
|  |  | q32.2^1^ | 209501836–209614549 | 113 | 1.11 | *LOC642587, MIR205* |
|  | 2 | p21 | 45168836–45169314 | 0.4 | 0.93 | *SIX3* |
|  |  | p11.2 | 89141608–89301214 | 160 | 1.37 |  |
|  |  | q14.1 | 117979245–118398138 | 419 | -1.09 |  |
|  | 3 | p14.2^1^ | 60515742–60597480 | 82 | -1.65 | *FHIT* |
|  |  | q27.3–q28 | 187497728–190341772 | 2844 | 0.65 | *LPP, TP63, CLDN1, CLDN16, IL1RAP* |
|  | 4 | q13.2 | 69392576–69483277 | 91 | -2.63 | *UGT2B17, UGT2B15* |
|  | 7 | p22.3–p11.2^1^ | 54185–57842077 | 57788 | 0.69 | *FAM20C, PDGFA, PRKAR1B, SUN1, ZFAND2A,  MAFK, MAD1L1, FTSJ2, NUDT1, EIF3B,  LFNG, GNA12, CARD11, RADIL, PAPOLB,  ACTB, FSCN1, RNF216, OCM, PMS2,  CYTH3, ZNF12, C1GALT1, COL28A1, RPA3,  ICA1, THSD7A, ETV1, DGKB, MEOX2,  SOSTDC1, AGR2, AGR3, AHR, SNX13,  PRPS1L1, HDAC9, TWISTNB,  MACC1, ABCB5,  SP8, SP4, DNAH11, CDCA7L, IL6,  FAM126A,  KLHL7, IGF2BP3, STK31, NPY,  MPP6, DFNA5, OSBPL3, CYCS,  NFE2L3,  HOXA1, HOXA2, HOXA3, HOXA4, HOXA5,  HOXA6,  HOXA7, HOXA9, MIR196B, HOXA10,  HOXA11, HOXA13, EVX1,  TAX1BP1, JAZF1,  CPVL, CHN2, WIPF3, ZNRF2, NOD1,  GGCT,  GARS, CRHR2, INMT, AQP1,  GHRHR, ADCYAP1R1, NEUROD6,  LSM5, AVL9,  NT5C3, RP9, BBS9, AAA1, NPSR1,  TBX20, SEPT7,  AOAH, ELMO1, TXNDC3,  SFRP4, STARD3NL, AMPH, VPS41,  POU6F2,  RALA, C7orf11, C7orf10, INHBA, GLI3,  PSMA2,  MRPL32, BLVRA, MRPS24, PGAM2,  POLD2, GCK, CAMK2B,  NPC1L1, TMED4,  OGDH, PPIA, CCM2, RAMP3, ADCY1,  IGFBP1, IGFBP3, TNS3, PKD1L1, HUS1,  UPP1, ABCA13, VWC2, IKZF1, DDC,  GRB10, HPVC1, EGFR, LANCL2, SEPT14,  MRPS17, GBAS, PHKG1* |
|  | 8 | p22 | 15952011–16010296 | 52 | 1.02 | *MSR1* |
|  |  | p11.22 | 39258894–39381514 | 123 | 4.72 | *ADAM5P, ADAM3A* |
|  |  | q11.21–q12.1 | 52127282–55854108 | 3727 | 0.78 | *RB1CC1, OPRK1, TCEA1, LYPLA1, MRPL15, SOX17, RP1* |
|  |  | q21.12–q21.3 | 86847986–88292389 | 1444 | 0.74 | *WWP1, FAM82B, CPNE3, CNGB3* |
|  | 9 | p23^1^ | 10023842–10101447 | 78 | -1.04 | *PTPRD* |
|  |  | q21.2–q21.31 | 80102875–82257893 | 2155 | 0.61 | *GNA14, GNAQ, PSAT1* |
|  | 11 | p15.5 | 2016675–2016774 | 0.1 | 1.76 | *H19* |
|  |  | p15.3–p15.2 | 10903031–14815348 | 3912 | 0.57 | *MICAL2, MICALCL, TEAD1, ARNTL, PTH,  SPON1, RRAS2,  PSMA1, PDE3B* |
|  |  | q21–q22.3 | 95519128–103695043 | 8176 | 0.76 | *MTMR2, MAML2, JRKL, CNTN5, PGR,  TRPC6, ANGPTL5, YAP1, TMEM123, MMP7,  MMP20, MMP8, MMP10, MMP1, MMP3,   MMP12, MMP13, DYNC2H1* |
|  | 12 | p13.31 | 9637323–9693948 | 57 | 4.21 |  |
|  | 13 | q31.1–q31.3 | 79553800–91458655 | 11905 | 0.53 | *NDFIP2, SPRY2, SLITRK1, SLITRK6, SLITRK5* |
|  |  | q32.3–q33.1 | 101587236–102442729 | 855 | 0.74 | *NALCN, ITGBL1, FGF14* |
|  | 14 | q32.33^1^ | 106371690–106538480 | 167 | 3.54 | *KIAA0125, ADAM6* |
|  |  | q32.33^1^ | 106803248–106957950 | 155 | 0.87 | *NCRNA00221* |
|  | 15 | q14 | 35582852–37588674 | 2006 | 0.56 | *MEIS2, ATPBD4, MIR3942, C15orf41, CSNK1A1P1,  LOC145845* |
|  | 16 | p11.2^1^ | 28861531–28929651 | 68 | 0.74 | *SH2B1, ATP2A1, RABEP2* |
|  |  | q22.1–q23.1^1^ | 68156107–77352056 | 9196 | 0.74 | *NFATC3, ESRP2, SLC7A6, ZFP90, CDH3,  CDH1, HAS3, CIRH1A, SNTB2, VPS4A,  PDF, COG8, TERF2, CYB5B, NQO1,  WWP2,  MIR140, AARS, COG4, SF3B3,  IL34, VAC14, HYDIN, CALB2,  ZNF23,  ZNF19, TAT, PHLPP2, AP1G1, PKD1L3,  HP, HPR,  DHX38, ZFHX3, PSMD7,  GLG1, FA2H, ZNRF1, LDHD, CTRB1,   BCAR1, CFDP1, CHST6, CHST5, GABARAPL2,  ADAT1, KARS,  TERF2IP, ADAMTS18* |
|  |  | q23.1 | 78633183–78779049 | 146 | -1.36 | *WWOX* |
|  | 17 | q21.31 | 44254355–44351152 | 97 | 0.94 | *KIAA1267, LOC644246* |
|  | 19 | q11–q12 | 28431784–29178957 | 747 | 0.55 |  |
|  | 20 | p13–p11.1^1^ | 67778–26312663 | 26245 | 0.72 | *SOX12, NRSN2, RBCK1, TBC1D20, CSNK2A1,  TCF15, ANGPT4,  RSPO4, SNPH, FKBP1A,  SIRPB2, SIRPG, PDYN, STK35, TGM3,  TGM6, TMC2, IDH3B, EBF4, PTPRA,  GNRH2, MRPS26, OXT,  AVP, ITPA,  SLC4A11, ATRN, ADAM33, HSPA12B, SPEF1,  CENPB, CDC25B, MAVS, PANK2, RNF24,  ADRA1D, PRNP, PRND,  SLC23A2, C20orf30,  PCNA, CDS2, PROKR2, CHGB, MCM8,   BMP2, HAO1, PLCB4, SNAP25, MKKS,  JAG1, SPTLC3, TASP1,  C20orf7, MACROD2,  FLRT3, OTOR, PCSK2, BFSP1, DSTN,   RRBP1, SNX5, ZNF133, RBBP9, SEC23B,  DTD1, SLC24A3, RIN2, CRNKL1, INSM1,  XRN2, NKX24, NKX22, PAX1, FOXA2,  SSTR4,  THBD, NXT1, NAPB, CST11,  CST3, CST4, CST1, CST2, CST5,   GGTLC1, VSX1, ENTPD6, PYGB, GINS1,  NANP, PLCB1* |
|  |  | q11.21–q13.33 | 29462044–62949149 | 33487 | 0.68 | *DEFB118, HM13, ID1, COX4I2, MYLK2,  PDRG1, HCK, KIF3B,  ASXL1, DNMT3B,  MAPRE1, BASE, PLUNC, CDK5RAP1,  CBFA2T2,  NECAB3, E2F1, CHMP4B, ASIP, AHCY,  ITCH,  DYNLRB1, PIGU, NCOA6, GGT7,  ACSS2, GSS, MYH7B, TRPC4AP, PROCR,  EIF6, UQCC, GDF5, CEP250, SPAG4,  NFS1, MYL9,  TGIF2, SLA2, NDRG3,  SAMHD1, RBL1, RPN2, GHRH, SRC,   NNAT, CTNNBL1, TGM2, BPI, LBP,  MAFB, TOP1, PLCG1, ZHX3, LPIN3,  SGK2, MYBL2, JPH2, HNF4A, SERINC3,  PKIG, ADA,  WISP2, KCNK15, RIMS4,  YWHAB, STK4, WFDC5, WFDC12, PI3,  SEMG1, SEMG2, MATN4, SDC4, SYS1,  DBNDD2, UBE2C, TNNC2, ACOT8, CTSA,  PLTP, ZNF335, MMP9, SLC12A5, CD40,  CDH22, ELMO2, SLC13A3, SLC2A10, EYA2,  NCOA3, SULF2, PREX1,  ARFGEF2, CSE1L,  KCNB1, PTGIS, B4GALT5, SLC9A8, SPATA2,  RNF114, SNAI1, UBE2V1, CEBPB, PTPN1,  PARD6B, BCAS4,  DPM1, KCNG1, NFATC2,  SALL4, ZNF217, BCAS1, CYP24A1,  PFDN4,  DOK5, MC3R, AURKA, TFAP2C, BMP7,  SPO11, RBM38, CTCFL, PCK1, ZBP1,  PMEPA1, RAB22A, VAPB, STX16, MIR296,  GNAS, TH1L, CTSZ, TUBB1, ATP5E,  EDN3, SYCP2, PPP1R3D,  CDH4, TAF4,  PSMA7, SS18L1, GTPBP5, OSBPL2, LAMA5,  RPS21, GATA5, SLCO4A1, NTSR1, C20orf20,  COL9A3, C20orf11,  SLC17A9, HAR1B, HAR1A,  BIRC7, NKAIN4, ARFGAP1, CHRNA4, KCNQ2,  EEF1A2, GMEB2, STMN3, RTEL1, TNFRSF6B,  LIME1, TPD52L2, UCKL1, SOX18* |
|  | 22 | q11.21 | 19747933–19749476 | 2 | -1.09 | *TBX1* |
|  |  | q11.22 | 23056562–23228483 | 172 | 3.79 | *MIR650* |
| HN4 | 1 | p13.2^1^ | 115419373–115493167 | 74 | -1.53 | *SYCP1* |
|  |  | q25.3^1^ | 180746048–180858503 | 112 | -2.67 | *XPR1* |
|  | 3 | p14.2^1^ | 60629805–61027287 | 397 | -1.70 | *FHIT* |
|  | 6 | q25.3^1^ | 158677452–158812696 | 135 | -2.31 | *TULP4* |
|  | 7 | p22.3–p11.2^1^ | 65558–57417402 | 57352 | 0.67 | *FAM20C, PDGFA, PRKAR1B, SUN1, ZFAND2A,  MAFK, MAD1L1, FTSJ2, NUDT1, EIF3B,  LFNG, GNA12, CARD11, RADIL,  PAPOLB,  ACTB, FSCN1, RNF216, OCM, PMS2,  CYTH3, ZNF12, C1GALT1, COL28A1, RPA3,  ICA1, THSD7A, ETV1, DGKB,  MEOX2,  SOSTDC1, AGR2, AGR3, AHR, SNX13,  PRPS1L1, HDAC9, TWISTNB, MACC1, ABCB5,  SP8, SP4, DNAH11, CDCA7L, IL6,  FAM126A, KLHL7, IGF2BP3, STK31, NPY,  MPP6, DFNA5,  OSBPL3, CYCS, NFE2L3,  HOXA1, HOXA2, HOXA3, HOXA4,  HOXA5,  HOXA6, HOXA7, HOXA9, MIR196B, HOXA10,  HOXA11, HOXA13, EVX1, TAX1BP1, JAZF1,  CPVL, CHN2, WIPF3, ZNRF2, NOD1,  GGCT, GARS, CRHR2, INMT, AQP1,  GHRHR,  ADCYAP1R1, NEUROD6, LSM5, AVL9,  NT5C3, RP9, BBS9, AAA1, NPSR1,  TBX20, SEPT7, AOAH, ELMO1, TXNDC3,  SFRP4,  STARD3NL, AMPH, VPS41, POU6F2,  RALA, C7orf11, C7orf10,  INHBA, GLI3,  PSMA2, MRPL32, BLVRA, MRPS24, PGAM2,   POLD2, GCK, CAMK2B, NPC1L1, TMED4,  OGDH, PPIA, CCM2, RAMP3, ADCY1,  IGFBP1, IGFBP3, TNS3, PKD1L1, HUS1,  UPP1, ABCA13, VWC2, IKZF1, DDC,  GRB10, HPVC1, EGFR, LANCL2,  SEPT14,  MRPS17, GBAS, PHKG1* |
|  | 8 | p11.22 | 39258894–39381514 | 123 | 4.56 | *ADAM5P, ADAM3A* |
|  |  | q11.21–q12.1 | 52101905–55756576 | 3655 | 0.73 | *RB1CC1, OPRK1, TCEA1, LYPLA1, MRPL15,  SOX17, RP1* |
|  |  | q21.2–q21.3 | 86868329–88385497 | 1517 | 0.75 | *WWP1, FAM82B, CPNE3, CNGB3* |
|  | 9 | q13–q34.3 | 68369294–140948378 | 72579 | 0.77 | *FOXD4L2, CBWD3, FOXD4L3, PGM5, PIP5K1B,  PRKACG, FXN, TJP2, APBA1, MAMDC2,  SMC5, TRPM3, MIR204, TMEM2, GDA,  ZFAND5, TMC1, ALDH1A1, ANXA1, RORB,  TRPM6, OSTF1, PCSK5, RFK, GCNT1,  PCA3, VPS13A, GNA14, GNAQ, PSAT1,  TLE1, FRMD3, UBQLN1, SLC28A3, NTRK2,  AGTPBP1, ISCA1, GAS1, DAPK1, SPIN1,  S1PR3, SECISBP2, GADD45G, SYK, AUH,  NFIL3, ROR2, SPTLC1, IARS, NOL8,  CENPP, OGN, ASPN, ECM2, BICD2,  NINJ1, WNK2, FAM120A, PHF2, BARX1,  MIRLET7A1, MIRLET7F1, MIRLET7D, ZNF169, FBP2,  FBP1, MIR23B, MIR27B, FANCC, PTCH1,  HSD17B3, SLC35D2, CDC14B, CTSL2, XPA,  FOXE1,  HEMGN, NANS, TRIM14, CORO2A,  TBC1D2, GABBR2, COL15A1, TGFBR1, ALG2,  STX17, INVS, TMEFF1, BAAT, MRPL50,   ZNF189, ALDOB, GRIN3A, CYLC2, ABCA1,  SLC44A1, FSD1L, FKTN, TAL2, RAD23B,  KLF4, ACTL7B, ACTL7A, IKBKAP, CTNNAL1,  MIR32, C9orf4, PALM2, AKAP2, TXN,  SVEP1, MUSK, LPAR1,  UGCG, ROD1,  SLC46A2, ZFP37, SLC31A1, PRPF4, ALAD,   POLE3, RGS3, AMBP, COL27A1, ORM1,  ORM2, DFNB31, ATP6V1G1, TNFSF15, TNFSF8,  TNC, DEC1, PAPPA, ASTN2, TRIM32,   TLR4, DBC1, CDK5RAP2, MEGF9, PSMD5,  PHF19, TRAF1, C5,  CEP110, RAB14,  GSN, GGTA1, NDUFA8, PTGS1, PDCL,  GPR21, STRBP, CRB2, LHX2, NEK6,  PSMB7, NR5A1, MIR181A2, MIR181B2, PPP6C,  HSPA5, GAPVD1, PBX3, LMX1B, ZBTB34,  SLC2A8,  ZNF79, RPL12, LRSAM1, STXBP1,  CDK9, FPGS, ENG, AK1,  PIP5KL1,  FAM102A, NAIF1, PTGES2, LCN2, DNM1,  TRUB2,  COQ4, SLC27A4, URM1, ODF2,  GLE1, SPTAN1, SET, PKN3,  ENDOG,  CCBL1, LRRC8A, CRAT, PPP2R4, PRRX2,  PTGES,  TOR1B, TOR1A, FNBP1, NCS1,  ASS1, ABL1, LAMC3, NUP214,  POMT1,  UCK1, RAPGEF1, MED27, SETX, TTF1,  BARHL1, TSC1, GFI1B, CEL, RALGDS,  GBGT1, OBP2B, ABO, SURF6, RPL7A,   SURF1, SURF2, SURF4, REXO4, ADAMTS13,  SLC2A6,  ADAMTSL2, DBH, SARDH, VAV2,  RXRA, COL5A1, FCN2, FCN1, OLFM1,  MRPS2, LCN1, OBP2A, PAEP, LCN9,  KCNT1, UBAC1,  LHX3, QSOX2, CARD9,  PMPCA, INPP5E, SEC16A, NOTCH1,  MIR126,  AGPAT2, LCN10, LCN6, LCN8, C9orf86,  PHPT1, C8G,  LCN12, PTGDS, CLIC3,  ABCA2, FUT7, NPDC1, ENTPD2,  C9orf140,  GRIN1, ANAPC2, SSNA1, SLC34A3, COBRA1,  NELF,  PNPLA7, MRPL41, ZMYND19, EHMT1,  CACNA1B* |
|  | 12 | p13.31 | 9637323–9713425 | 76 | 3.37 |  |
|  | 14 | q32.33^1^ | 106453638–106538480 | 85 | 4.05 |  |
|  | 15 | q14–q26.3 | 34656203–102465355 | 67809 | 0.63 | *GOLGA8B, GJD2, ACTC1, MEIS2, SPRED1,  RASGRP1, THBS1,  GPR176, SRP14, BMF,  BUB1B, PAK6, PLCB2, IVD, FAM82A2,   DLL4, INO80, CHP, NUSAP1, NDUFAF1,  RTF1, ITPKA, LTK,  TYRO3, MGA,  PLA2G4B, SPTBN5, EHD4, PLA2G4D, VPS39,   GANC, CAPN3, SNAP23, CDAN1, TTBK2,  UBR1, CCNDBP1,  EPB42, TGM7, TUBGCP4,  TP53BP1, MAP1A, CKMT1B, STRC,  ELL3,  MFAP1, EIF3J, SPG11, B2M, SORD,  DUOX2, DUOXA2,  DUOXA1, DUOX1, SLC28A2,  GATM, SLC30A4, PLDN, SLC24A5, SLC12A1,  DUT, FBN1, EID1, COPS2, GALK2,  FGF7, SLC27A2,  HDC, GABPB1, TRPM7,  SPPL2A, AP4E1, CYP19A1, GLDN,  DMXL2,  SCG3, TMOD2, TMOD3, LEO1, BCL2L10,  GNB5,  MYO5C, MYO5A, ARPP19, ONECUT1,  RAB27A, PIGB, DYX1C1, NEDD4, RFX7,  MNS1, TCF12, CGNL1, GRINL1A, ALDH1A2,   AQP9, LIPC, ADAM10, MYO1E, GCNT3,  ANXA2, NARG2, RORA, TLN2, TPM1,  LACTB, RPS27L, APH1B, CA12, USP3,  SNX1,  CSNK1G1, KIAA0101, PIF1, SPG21,  MTFMT, PDCD7, CILP,  DPP8, SLC24A1,  RAB11A, MEGF11, TIPIN, MAP2K1, RPL4,   ZWILCH, SMAD6, SMAD3, IQCH, MAP2K5,  PIAS1, CLN6,  ITGA11, CORO2B, ANP32A,  NOX5, GLCE, KIF23, RPLP1, TLE3,  UACA, THAP10, NR2E3, MYO9A, PKM2,  CELF6, HEXA, ARIH1,  BBS4, ADPGK,  NEO1, HCN4, NPTN, CD276, LOXL1,  STOML1,  PML, ISLR, STRA6, SEMA7A,  UBL7, ARID3B, CLK3, CYP1A1,  CYP1A2,  CSK, SCAMP2, MPI, COX5A, PPCDC,  MAN2C1, SIN3A, IMP3, CSPG4, FBXO22,  NRG4, ETFA, SCAPER, RCN2, PSTPIP1,  HMG20A, LINGO1, IDH3A, CRABP1, IREB2,  PSMA4, CHRNA5,  CHRNA3, CHRNB4, ADAMTS7,  MORF4L1, CTSH, BCL2A1,  ZFAND6, FAH,  ARNT2, KIAA1199, MESDC2, IL16, STARD5,   MEX3B, RPS17, AP3B2, SCARNA15, HOMER2,  TM6SF1, BNC1,  SH3GL3, ADAMTSL3, NMB,  SLC28A1, PDE8A, AKAP13, NTRK3, MRPL46,  MRPS11, AEN, ISG20, ACAN, MFGE8,  ABHD2, RLBP1, FANCI, POLG, MIR93,  RHCG, PEX11A, MESP2, ANPEP, IDH2,   IQGAP1, CRTC3, BLM, FURIN, FES,  MAN2A2, UNC45A, VPS33B, SLCO3A1, CHD2,  RGMA, NR2F2, IGF1R, MEF2A, ALDH1A3,   LRRK1, CHSY1, SELS, SNRPA1, PCSK6,  TM2D3* |
|  | 17 | q23^1^ | 78008301–78111444 | 103 | -2.76 | *GAA, TBC1D16, CCDC40* |
|  | 20 | q11.21–q13.33 | 29483588–62880583 | 33397 | 0.66 | *DEFB118, HM13, ID1, COX4I2, MYLK2,  PDRG1, HCK, KIF3B,  ASXL1, DNMT3B,  MAPRE1, BASE, PLUNC, CDK5RAP1,  CBFA2T2,  NECAB3, E2F1, CHMP4B, ASIP, AHCY,  ITCH,  DYNLRB1, PIGU, NCOA6, GGT7,  ACSS2, GSS, MYH7B, TRPC4AP, PROCR,  EIF6, UQCC, GDF5, CEP250, SPAG4,  NFS1, MYL9,  TGIF2, SLA2, NDRG3,  SAMHD1, RBL1, RPN2, GHRH, SRC,   NNAT, CTNNBL1, TGM2, BPI, LBP,  MAFB, TOP1, PLCG1, ZHX3, LPIN3,  SGK2, MYBL2, JPH2, HNF4A, SERINC3,  PKIG, ADA,  WISP2, KCNK15, RIMS4,  YWHAB, STK4, WFDC5, WFDC12, PI3,  SEMG1, SEMG2, MATN4, SDC4, SYS1,  DBNDD2, UBE2C, TNNC2, ACOT8, CTSA,  PLTP, ZNF335, MMP9, SLC12A5, CD40,  CDH22, ELMO2, SLC13A3, SLC2A10, EYA2,  NCOA3, SULF2, PREX1,  ARFGEF2, CSE1L,  KCNB1, PTGIS, B4GALT5, SLC9A8, SPATA2,  RNF114, SNAI1, UBE2V1, CEBPB, PTPN1,  PARD6B, BCAS4,  DPM1, KCNG1, NFATC2,  SALL4, ZNF217, BCAS1, CYP24A1,  PFDN4,  DOK5, MC3R, AURKA, TFAP2C, BMP7,  SPO11, RBM38, CTCFL, PCK1, ZBP1,  PMEPA1, RAB22A, VAPB, STX16, MIR296,  GNAS, TH1L, CTSZ, TUBB1, ATP5E,  EDN3, SYCP2, PPP1R3D,  CDH4, TAF4,  PSMA7, SS18L1, GTPBP5, OSBPL2, LAMA5,  RPS21, GATA5, SLCO4A1, NTSR1, C20orf20,  COL9A3, C20orf11,  SLC17A9, HAR1B, HAR1A,  BIRC7, NKAIN4, ARFGAP1, CHRNA4, KCNQ2,  EEF1A2, GMEB2, STMN3, RTEL1, TNFRSF6B,  LIME1,  TPD52L2, UCKL1, SOX18* |
| HN12 | 1 | p31.1^1^ | 72329328–72493839 | 165 | -0.95 | *NEGR1* |
|  |  | p22.3–p21.2^1^ | 85988179–101724358 | 15736 | 0.67 | *DDAH1, CYR61, COL24A1, CLCA2, CLCA1,  SEP15, HS2ST1,  LMO4, PKN2, GTF2B,  CCBL2, GBP3, GBP1, GBP2, GBP7,  GBP4, GBP5, GBP6, LRRC8B, LRRC8C,  LRRC8D, CDC7, TGFBR3,  BRDT, GFI1,  EVI5, RPL5, MTF2, DR1, GCLM,  ABCA4,  ARHGAP29, ABCD3, F3, CNN3,  ALG14, PTBP2, DPYD, LPPR4,  FRRS1,  AGL, SLC35A3, DBT, CDC14A, VCAM1,  EXTL2, SLC30A7, DPH5, S1PR1* |
|  |  | q23.1–q23.2^1^ | 158043247–160135187 | 2092 | 0.61 | *CD1D, CD1A, CD1C, CD1B, CD1E,  SPTA1, MNDA, PYHIN1,  IFI16, AIM2,  FCER1A, APCS, CRP, SLAMF8, CCDC19,  TAGLN2, IGSF9, PIGM, KCNJ10, IGSF8,  ATP1A2, ATP1A4* |
|  |  | q44 | 246261013–246341018 | 80 | -1.62 | *SMYD3* |
|  | 2 | p21 | 45168836–45169314 | 0.5 | 1.05 | *SIX3* |
|  |  | p11.2 | 89141608–89258800 | 117 | 1.56 |  |
|  |  | q22.1^1^ | 141735849–141882126 | 146 | -0.90 | *LRP1B* |
|  |  | q32.1–q32.3 | 185591359–194235687 | 8644 | 0.53 | *ZNF804A, ITGAV, CALCRL, TFPI, GULP1,  DIRC1, COL3A1, COL5A2, SLC40A1, PMS1,  MSTN, HIBCH, INPP1, NAB1, GLS,  STAT1, STAT4, MYO1B, OBFC2A, SDPR,  TMEFF2, PCGEM1* |
|  |  | q33.3 | 205559838–205624937 | 65 | -1.60 | *PARD3B* |
|  | 3 | p13^1^ | 71506648–71724981 | 218 | -1.23 | *FOXP1, MIR1284* |
|  |  | p12.2^1^ | 81413060–82635750 | 1223 | 0.64 | *GBE1* |
|  | 4 | q13.2 | 69392576–69483277 | 91 | 0.56 | *UGT2B17, UGT2B15* |
|  |  | q22.1^1^ | 91842748–91940897 | 98 | -1.53 | *FAM190A* |
|  |  | q31.21 | 143140695–143249560 | 109 | -1.60 | *INPP4B* |
|  |  | q31.3 | 152332224–153015219 | 683 | -1.03 | *FAM160A1, PET112L* |
|  | 5 | p15.33–p11^1^ | 22149–46365277 | 46,343 | 0.99 | *SDHA, AHRR, SLC9A3, TPPP, SLC12A7,  SLC6A19, SLC6A18, TERT, CLPTM1L, SLC6A3,  LPCAT1, MRPL36, NDUFS6, IRX4, IRX2,  IRX1, ADAMTS16, MED10, NSUN2, SRD5A1,  ADCY2, MTRR, CCT5, ROPN1L, DAP,  CTNND2, DNAH5, TRIO, ANKH, FBXL7,  MYO10, BASP1, CDH18, CDH12, PMCHL1,  CDH10, CDH9, CDH6, DROSHA, PDZD2,  GOLPH3, MTMR12, NPR3, TARS, ADAMTS12,  SLC45A2, AMACR, RAI14, RAD1, AGXT2,  PRLR, IL7R, SKP2, SLC1A3, NIPBL,  NUP155, GDNF, LIFR, OSMR, C9,  DAB2, PTGER4, PRKAA1, RPL37, CARD6,  C7, C6, OXCT1, FBXO4, GHR,  SEPP1, ZNF131, HMGCS1, CCL28, PAIP1,  NNT, FGF10, MRPS30* |
|  | 6 | p12.1^1^ | 56252017–56548108 | 296 | 0.75 | *DST* |
|  |  | p12.1–p11.1^1^ | 56677461–58774324 | 2097 | 0.55 | *RAB23, BEND6, KIAA1586, ZNF451* |
|  |  | q14.3–q21 | 87044032–108146477 | 21102 | -1.24 | *HTR1E, CGA, GJB7, SLC35A1, RARS2,  ORC3, SPACA1, CNR1, RNGTT, PNRC1,  GABRR1, GABRR2, RRAGD, ANKRD6, CASP8AP2,  GJA10, BACH2, MAP3K7, MANEA, FUT9,  GPR63, NDUFAF4, POU3F2, FBXL4, CCNC,  MCHR2, SIM1, GRIK2, LIN28B, BVES,  PREP, PRDM1, AIM1, PDSS2* |
|  | 7 | p22.3–p11.2 | 54185–57262076 | 57208 | 0.73 | *FAM20C, PDGFA, PRKAR1B, SUN1, ZFAND2A,  MAFK, MAD1L1, FTSJ2, NUDT1, EIF3B,  LFNG, GNA12, CARD11, RADIL, PAPOLB,  ACTB, FSCN1, RNF216, OCM, PMS2,  CYTH3, ZNF12, C1GALT1, COL28A1, RPA3,  ICA1, THSD7A, ETV1, DGKB, MEOX2,  SOSTDC1, AGR2, AGR3, AHR, SNX13,  PRPS1L1, HDAC9, TWISTNB, MACC1, ABCB5,  SP8, SP4, DNAH11, CDCA7L, IL6,  FAM126A, KLHL7, IGF2BP3, STK31, NPY,  MPP6, DFNA5, OSBPL3, CYCS, NFE2L3,  HOXA1, HOXA2, HOXA3, HOXA4, HOXA5,  HOXA6, HOXA7, HOXA9, MIR196B, HOXA10,  HOXA11, HOXA13, EVX1, TAX1BP1, JAZF1,  CPVL, CHN2, WIPF3, ZNRF2, NOD1,  GGCT, GARS, CRHR2, INMT, AQP1,  GHRHR, ADCYAP1R1, NEUROD6, LSM5, AVL9,  NT5C3, RP9, BBS9, AAA1, NPSR1,  TBX20, SEPT7, AOAH, ELMO1, TXNDC3,  SFRP4, STARD3NL, AMPH, VPS41, POU6F2,  RALA, C7orf11, C7orf10, INHBA, GLI3,  PSMA2, MRPL32, BLVRA, MRPS24, PGAM2,  POLD2, GCK, CAMK2B, NPC1L1, TMED4,  OGDH, PPIA, CCM2, RAMP3, ADCY1,  IGFBP1, IGFBP3, TNS3, PKD1L1, HUS1,  UPP1, ABCA13, VWC2, IKZF1, DDC,  GRB10, HPVC1, EGFR, LANCL2, SEPT14,  MRPS17, GBAS, PHKG1* |
|  |  | q11.21 | 64440267–66709382 | 2269 | 1.56 | *ZNF117, ERV3, ZNF92, GUSB, ASL,  TPST1, KCTD7, RABGEF1, SBDS* |
|  | 8 | q11.1–q12.3 | 47735940–63817073 | 16081 | 0.74 | *CEBPD, PRKDC, MCM4, SNAI2, SNTG1,  RB1CC1, OPRK1, TCEA1, LYPLA1, MRPL15,  SOX17, RP1, TGS1, LYN, RPS20,  MOS, PLAG1, CHCHD7, PENK, CYP7A1,  NSMAF, TOX, CA8, RAB2A, CHD7,  ASPH, NKAIN3* |
|  | 9 | p23^1^ | 9400269–9782886 | 383 | -1.24 | *PTPRD* |
|  |  | p21.3 | 20809914–20951944 | 142 | -1.48 | *KIAA1797* |
|  |  | p21.3^1^ | 21993401–22036505 | 43 | -5.43 | *CDKN2A, CDKN2B* |
|  | 10 | q24.2–q24.31 | 99970823–102300490 | 2330 | 0.62 | *LOXL4, HPS1, CNNM1, GOT1, SLC25A28,  COX15, CUTC, ABCC2, CPN1, ERLIN1,  CHUK, BLOC1S2, PKD2L1, SCD, WNT8B,  NDUFB8, HIF1AN* |
|  | 11 | p15.5 | 2016612–2017012 | 0.4 | 1.34 | *H19* |
|  |  | p15.5 | 2017725–2020975 | 3 | 0.57 | *H19, MIR675* |
|  |  | q11 | 55385617–55450788 | 65 | -1.76 | *OR4P4, OR4S2, OR4C6* |
|  |  | q13.2–q22.3^1^ | 67793815–105046236 | 37,252 | 0.84 | *ALDH3B1, NDUFS8, TCIRG1, SUV420H1, C11orf24,  LRP5, GAL, MTL5, CPT1A, MRPL21,  IGHMBP2, MRGPRD, MRGPRF, TPCN2, MYEOV,  CCND1, ORAOV1, FGF19, FGF4, FGF3,  ANO1, FADD, PPFIA1, CTTN, SHANK2,  DHCR7, NADSYN1, KRTAP5–9, NUMA1, LRTOMT,  FOLR1, FOLR2, INPPL1, PHOX2A, ARAP1,  P2RY2, P2RY6, RELT, PLEKHB1, RAB6A,  MRPL48, CHCHD8, UCP2, UCP3, PPME1,  P4HA3, PGM2L1, KCNE3, XRRA1, NEU3,  ARRB1, RPS3, DGAT2, UVRAG, WNT11,  C11orf30, TSKU, CAPN5, OMP, MYO7A,  PAK1, AQP11, CLNS1A, RSF1, THRSP,  ALG8, GAB2, NARS2, PRCP, RAB30,  DLG2, TMEM126A, SYTL2, PICALM, EED,  ME3, FZD4, RAB38, CTSC, TYR,  NOX4, FOLH1B, NAALAD2, FAT3, MTNR1B,  TAF1D, GPR83, MRE11A, FUT4, MTMR2,  MAML2, JRKL, CNTN5, PGR, TRPC6,  ANGPTL5, YAP1, TMEM123, MMP7, MMP20,  MMP8, MMP10, MMP1, MMP3, MMP12,  MMP13, DYNC2H1, PDGFD, CASP12, CASP4,  CASP5, CASP1* |
|  |  | q24.1–q25^1^ | 121308802–134934196 | 13625 | -0.90 | *SORL1, MIR125B1, BLID, MIRLET7A2, UBASH3B,  CRTAM, HSPA8, CLMP, SCN3B, ZNF202,  TBRG1, NRGN, ROBO3, ROBO4, HEPN1,  HEPACAM, PKNOX2, FEZ1, EI24, CHEK1,  ACRV1, HYLS1, SRPR, TIRAP, KIRREL3,  ETS1, FLI1, KCNJ1, KCNJ5, BARX2,  NFRKB, APLP2, ST14, ADAMTS8, NTM,  OPCML, SPATA19, JAM3, VPS26B, ACAD8,  B3GAT1* |
|  | 12 | p13.31 | 9637323–9693948 | 57 | 2.99 |  |
|  | 13 | q12.11 | 19590141–21861250 | 2271 | -1.19 | *MPHOSPH8, PSPC1, GJA3, GJB2, GJB6,  CRYL1, IL17D, LATS2, MRP63* |
|  |  | q14.3^1^ | 51159942–51229962 | 70 | -4.20 |  |
|  | 14 | q11.2^1^ | 19265142–20421677 | 1157 | 0.86 | *OR11H12, POTEG, POTEM* |
|  |  | q21.1^1^ | 41616413–41657239 | 41 | -1.18 |  |
|  |  | q21.2^1^ | 45565742–45604491 | 39 | -1.05 | *PRPF39, SNORD127, FKBP3* |
|  |  | q32.33^1^ | 106371690–106538480 | 167 | 3.59 | *KIAA0125, ADAM6* |
|  | 16 | p13.3 | 1634585–1804845 | 170 | -1.47 | *IFT140, CRAMP1L, HN1L, MAPK8IP3, MIR3177* |
|  |  | p11.2–p11.1^1^ | 34226241–35148939 | 923 | -0.98 | *UBE2MP1, LOC283914, LOC146481, LOC100130700, FLJ26245* |
|  |  | q23.1^1^ | 78206297–78818301 | 612 | -1.76 | *WWOX* |
|  | 17 | p12^1^ | 11529154–14822108 | 3293 | 0.61 | *DNAH9, ZNF18, MYOCD, ELAC2, HS3ST3A1,  COX10, HS3ST3B1* |
|  |  | q24.2–q25.1 | 65356908–73108973 | 7752 | 0.74 | *KPNA2, ARSG, PRKAR1A, FAM20A, ABCA8,  ABCA9, ABCA6, ABCA10, ABCA5, MAP2K6,  KCNJ16, KCNJ2, SOX9, SSTR2, COG1,  CDC42EP4, RPL38, DNAI2, GPRC5C, CD300LB,  RAB37, CD300LF, SLC9A3R1, GRIN2C, FDXR,  USH1G, C17orf28* |
|  | 18 | q12.1 | 27459126–28515161 | 1056 | 1.34 | *MIR302F* |
|  |  | q12.1–q23^1^ | 28552510–78012829 | 49460 | -1.39 | *DSC3, DSC2, DSC1, DSG1, DSG4,  DSG3, DSG2, TTR, B4GALT6, MEP1B,  DTNA, MAPRE2, ZNF397, ZNF271, ZNF24,  ZNF396, GALNT1, MIR187, FHOD3, CELF4,  SYT4, SETBP1, SLC14A2, SLC14A1, ATP5A1,  SMAD2, SMAD7, DYM, RPL17, LIPG,  MYO5B, MBD1, MAPK4, MRO, ME2,  ELAC1, SMAD4, MEX3C, DCC, MBD2,  POLI, STARD6, TCF4, ONECUT2, FECH,  NARS, ATP8B1, NEDD4L, MALT1, GRP,  RAX, LMAN1, CCBE1, MC4R, CDH20,  PIGN, TNFRSF11A, BCL2, VPS4B, SERPINB5,  SERPINB13, SERPINB4, SERPINB3, SERPINB2, SERPINB10,  HMSD, SERPINB8, CDH7, CDH19, DSEL,  CD226, CBLN2, NETO1, FBXO15, CYB5A,  ZNF236, MBP, GALR1, SALL3, NFATC1,  CTDP1, TXNL4A, PARD6G* |
|  | 19 | p13.2 | 8476231–12111843 | 3636 | 0.61 | *MYO1F, ADAMTS10, OR7D4, ZNF266, ZNF121,  UBL5, PIN1, COL5A3, RDH8, ANGPTL6,  PPAN, P2RY11, EIF3G, DNMT1, S1PR2,  MRPL4, ICAM1, ICAM5, RAVER1, ICAM3,  TYK2, PDE4A, KEAP1, S1PR5, CDKN2D,  AP1M2, SLC44A2, ILF3, QTRT1, DNM2,  MIR199A1, TMED1, LDLR, EPOR, PRKCSH,  ELAVL3, ECSIT, CNN1, ACP5, ZNF627,  ZNF69* |
|  | 21 | q22.12 | 36205879–36232084 | 26 | -1.23 | *RUNX1* |
|  | 22 | q11.21–q11.22 | 18230460–23442209 | 5212 | 0.90 | *BID, MICAL3, PEX26, TUBA8, USP18,  DGCR6, PRODH, DGCR2, TSSK2, SLC25A1,  CLTCL1, HIRA, UFD1L, CLDN5, SEPT5,  GP1BB, TBX1, GNB1L, TXNRD2, COMT,  ARVCF, DGCR8, TRMT2A, ZDHHC8, RTN4R,  DGCR6L, RIMBP3, ZNF74, PI4KA, SERPIND1,  SNAP29, CRKL, LZTR1, SLC7A4, BCRP2,  RIMBP3C, RIMBP3B, HIC2, UBE2L3, SDF2L1,  PPIL2, YPEL1, MAPK1, TOP3B, VPREB1,  PRAME, GGTLC2, RTDR1, GNAZ* |
|  |  | q12.2 | 30413840–30647965 | 234 | 1.03 | *MTMR3, LIF* |

^1^The region was reported in The Cancer Genome Atlas (TCGA) [29].
