# Supplementary material for: Genomic Alteration in Head and Neck Squamous Cell Carcinoma (HNSCC) Cell Lines Inferred from Karyotyping, Molecular Cytogenetics, and Array Comparative Genomic Hybridization
Source: PLoS One. 2016 Aug 8;11(8):e0160901. doi: 10.1371/journal.pone.0160901 (PMC4976893; doi:10.1371/journal.pone.0160901)
Supplement: S2 Table — (DOCX) [file pone.0160901.s010.docx]

**S2 Table** Genome view of chromosome copy number variation (CNV) in HN30 cell line.

| chromosome | start–stop (bp) | size (kb) | cytoband | #probes | amp/del | p–value | annotations |
| --- | --- | --- | --- | --- | --- | --- | --- |
| chr1 | 16346492–16383299 | 37 | p36.13 | 31 | 0.35 | 2.70E–10 | *CLCNKA, CLCNKB* |
| chr1 | 89074748–89218821 | 144 | p22.2 | 6 | -0.96 | 1.56E–15 | *PKN2* |
| chr1 | 102541046–121330906 | 18,790 | p21.1 – p11.2 | 682 | -0.90 | NA | *COL11A1, RNPC3, AMY2B…* |
| chr1 | 196744721–196799302 | 55 | q31.3 | 3 | -3.80 | NA | *CFHR3, CFHR1* |
| chr2 | 89141608–89301214 | 160 | p11.2 | 7 | 1.56 | 1.90E–36 |  |
| chr3 | 117735–48924847 | 48,807 | p26.3 – p21.31 | 1845 | 0.55 | NA | *CHL1, CNTN6, CNTN4...* |
| chr3 | 3529292–3587832 | 59 | p26.2 | 4 | -1.11 | 5.74E–26 |  |
| chr3 | 7442823–7657894 | 215 | p26.1 | 8 | -0.90 | 1.18E–35 | *GRM7* |
| chr3 | 85615568–85870596 | 255 | p12.1 | 10 | -0.90 | 1.10E–19 | *CADM2* |
| chr3 | 123520161–146985724 | 23,466 | q21.1 – q24 | 776 | 0.56 | NA | *MYLK, CCDC14, ROPN1...* |
| chr4 | 72447–19532988 | 19,461 | p16.3 – p15.31 | 704 | -0.80 | NA | *ZNF595, ZNF718, ZNF876P...* |
| chr4 | 1986020–1988403 | 2 | p16.3 | 5 | 0.44 | 9.93E–19 | *WHSC2, MIR943* |
| chr4 | 13106682–13360120 | 253 | p15.33 | 10 | -4.33 | NA | *HSP90AB2P* |
| chr4 | 69392576–69483277 | 91 | q13.2 | 4 | 1.12 | 6.54E–13 | *UGT2B17, UGT2B15* |
| chr4 | 70148989–70230159 | 81 | q13.2 | 3 | -2.31 | 4.46E–29 | *UGT2B28* |
| chr4 | 144842588–144905281 | 63 | q31.21 | 3 | -1.30 | 4.88E–13 |  |
| chr4 | 180247704–190469337 | 10,222 | q34.3 – q35.2 | 361 | -0.90 | NA | *NCRNA00290, MGC45800, MIR1305...* |
| chr5 | 22149–46365277 | 46,343 | p15.33 – p11 | 1645 | 1.28 | NA | *PLEKHG4B, LRRC14B, CCDC127...* |
| chr5 | 1340550–5932396 | 4,592 | p15.33 – p15.32 | 150 | 1.54 | 1.33E–24 | *CLPTM1L, SLC6A3, LPCAT1...* |
| chr5 | 31538275–44613465 | 13,075 | p13.3 – p12 | 453 | 1.19 | 4.02E–10 | *C5orf22, PDZD2, MIR4279...* |
| chr5 | 83617152–83816674 | 200 | q14.3 | 7 | -0.99 | 7.55E–17 | *EDIL3* |
| chr5 | 112480515–114095601 | 1,615 | q22.2 – q22.3 | 54 | -0.91 | NA | *MCC, TSSK1B, YTHDC2...* |
| chr7 | 54185–57923933 | 57,870 | p22.3 – p11.2 | 2072 | 1.46 | NA | *FAM20C, LOC100288524, LOC442497…* |
| chr7 | 54185–3190067 | 3,136 | p22.3 – p22.2 | 126 | 1.64 | 3.02E–10 | *FAM20C, LOC100288524, LOC442497…* |
| chr7 | 3384223–3470044 | 86 | p22.2 | 4 | -0.90 | NA | *SDK1* |
| chr7 | 8751128–8971052 | 220 | p21.3 | 8 | -1.16 | NA | *NXPH1* |
| chr7 | 9155395–49095021 | 39,940 | p21.3 – p12.2 | 1403 | 1.52 | 7.34E–13 | *PER4, NDUFA4, PHF14...* |
| chr7 | 54224697–57923933 | 3,699 | p11.2 | 138 | 1.00 | NA | *HPVC1, VSTM2A, SEC61G...* |
| chr7 | 61059509–118972081 | 57,913 | q11.1 – q31.31 | 2372 | 0.96 | NA | *LOC643955, LOC100287704, LOC100287834...* |
| chr7 | 78554758–84763446 | 6,209 | q21.11 | 208 | 1.12 | 8.60E–14 | *MAGI2, MIR548M, GNAI1...* |
| chr8 | 686538–15306009 | 14,619 | p23.3 – p22 | 541 | -0.90 | NA | *LOC286083, DLGAP2, CLN8...* |
| chr8 | 3460000–4047203 | 587 | p23.2 | 19 | -3.83 | NA | *CSMD1* |
| chr8 | 12241093–12467543 | 226 | p23.1 | 4 | 0.64 | 6.98E–22 | *FAM66A, DEFB109P1, FAM90A25P...* |
| chr8 | 15952011–16241027 | 289 | p22 | 10 | 0.63 | 1.98E–10 | *MSR1* |
| chr8 | 24193014–29000762 | 4,808 | p21.2 – p12 | 161 | -0.91 | NA | *ADAM28, ADAMDEC1, ADAM7...* |
| chr8 | 39258894–39381514 | 123 | p11.22 | 5 | 4.79 | NA | *ADAM5P, ADAM3A* |
| chr8 | 55445546–146280020 | 90,834 | q11.23 – q24.3 | 3259 | 0.57 | NA | *RP1, XKR4, SBF1P1...* |
| chr10 | 102539–38649695 | 38,547 | p15.3 – p11.1 | 1466 | -0.92 | NA | *ZMYND11, DIP2C, C10orf108...* |
| chr10 | 34035598–34609123 | 574 | p11.22 – p11.21 | 21 | -3.84 | NA | *PARD3* |
| chr11 | 210300–51538651 | 51,328 | p15.5 – p11.12 | 2197 | 0.34 | NA | *RIC8A, SIRT3, PSMD13...* |
| chr11 | 54829323–134934196 | 80,105 | q11 – q25 | 2682 | 0.34 | NA | *TRIM48, OR4A16, OR4A15...* |
| chr12 | 62636682–62918916 | 282 | q14.1 | 9 | -1.01 | 1.91E–21 | *USP15, MON2* |
| chr13 | 36729959–44826157 | 8,096 | q13.3 – q14.11 | 282 | -0.65 | NA | *SOHLH2, C13orf38– SOHLH2, C13orf38…* |
| chr14 | 19265142–107287505 | 88,022 | q11.2 – q32.33 | 3360 | 0.53 | NA | *OR11H12, POTEG, POTEM...* |
| chr14 | 41616413–41657239 | 41 | q21.1 | 3 | -0.79 | 2.25E–13 |  |
| chr14 | 106371690–106538480 | 167 | q32.33 | 5 | 4.24 | NA | *KIAA0125, ADAM6* |
| chr14 | 106602267–106957950 | 356 | q32.33 | 11 | 1.57 | 7.96E–28 | *NCRNA00226, NCRNA00221* |
| chr15 | 20481702–22698581 | 2,217 | q11.1 – q11.2 | 25 | 0.75 | 4.61E–32 | *HERC2P3, GOLGA6L6, GOLGA8C...* |
| chr17 | 12728154–13549498 | 821 | p12 | 26 | 0.40 | 5.23E–11 | *ARHGAP44, ELAC2, HS3ST3A1* |
| chr17 | 57751694–57999267 | 248 | q23.1 | 10 | -0.90 | 6.41E–19 | *CLTC, PTRH2, VMP1...* |
| chr18 | 77992253–78012829 | 21 | q23 | 6 | -0.93 | 2.67E–13 | *PARD6G* |
| chr20 | 67778–26312663 | 26,245 | p13 – p11.1 | 927 | 0.94 | NA | *DEFB125, DEFB126, DEFB127...* |
| chr20 | 29462044–62949149 | 33,487 | q11.21 – q13.33 | 1289 | 0.91 | NA | *FRG1B, DEFB115, DEFB116...* |
| chr22 | 19747165–19747992 | 1 | q11.21 | 4 | 1.10 | 2.11E–12 | *TBX1* |
| chr22 | 23056562–23228483 | 172 | q11.22 | 7 | 3.76 | NA | *MIR650* |

NA indicates expression not detectable.
