# Supplementary material for: Genomic Alteration in Head and Neck Squamous Cell Carcinoma (HNSCC) Cell Lines Inferred from Karyotyping, Molecular Cytogenetics, and Array Comparative Genomic Hybridization
Source: PLoS One. 2016 Aug 8;11(8):e0160901. doi: 10.1371/journal.pone.0160901 (PMC4976893; doi:10.1371/journal.pone.0160901)
Supplement: S3 Table — (DOCX) [file pone.0160901.s011.docx]

**S3 Table** Genome view of chromosome copy number variation (CNV) in HN31 cell line.

| chromosome | start–stop (bp) | size (kb) | cytoband | #probes | amp/del | p–value | annotations |
| --- | --- | --- | --- | --- | --- | --- | --- |
| chr1 | 6999378–7084687 | 85 | p36.31 | 5 | 0.89 | 1.75E–14 | *CAMTA1* |
| chr1 | 143730352–249212668 | 105,482 | q21.1 – q44 | 3650 | 0.44 | NA | *FLJ39739, LOC100286793, PPIAL4G...* |
| chr1 | 209501836–209614549 | 113 | q32.2 | 5 | 1.11 | 5.32E–10 | *LOC642587, MIR205* |
| chr2 | 45168836–45169314 | 0 | p21 | 4 | 0.93 | 2.38E–12 | *SIX3* |
| chr2 | 45169685–45171338 | 2 | p21 | 16 | -0.31 | 1.31E–11 | *SIX3* |
| chr2 | 45172007–45172394 | 0 | p21 | 7 | 0.68 | 6.09E–11 | *SIX3* |
| chr2 | 89141608–89301214 | 160 | p11.2 | 7 | 1.37 | 2.80E–45 |  |
| chr2 | 117979245–118398138 | 419 | q14.1 | 17 | -1.09 | NA |  |
| chr3 | 62199–90458199 | 90,396 | p26.3 – p11.1 | 3256 | -0.50 | NA | *CHL1, CNTN6, CNTN4...* |
| chr3 | 62199–8547786 | 8,486 | p26.3 – p26.1 | 458 | -0.62 | 3.27E–23 | *CHL1, CNTN6, CNTN4...* |
| chr3 | 22940916–39149735 | 16,209 | p24.3 – p22.2 | 540 | -0.43 | 4.30E–14 | *UBE2E2, UBE2E1, NKIRAS1...* |
| chr3 | 60515742–60597480 | 82 | p14.2 | 3 | -1.65 | 2.16E–14 | *FHIT* |
| chr3 | 60629805–60877621 | 248 | p14.2 | 9 | -4.59 | NA | *FHIT* |
| chr3 | 60922966–61027287 | 104 | p14.2 | 5 | -1.60 | 4.69E–21 | *FHIT* |
| chr3 | 93538467–197837049 | 104,299 | q11.1 – q29 | 3583 | 0.44 | NA | *PROS1, ARL13B, STX19...* |
| chr3 | 187497728–190341772 | 2,844 | q27.3 – q28 | 146 | 0.65 | 5.35E–25 | *LOC339929, LPP, FLJ42393...* |
| chr3 | 195413025–195457706 | 45 | q29 | 3 | -0.62 | 1.63E–14 | *SDHAP2, MIR570, MUC20* |
| chr4 | 34787128–49505025 | 14,718 | p15.1 – p11 | 505 | -0.45 | NA | *ARAP2, DTHD1, KIAA1239...* |
| chr4 | 52701080–190790881 | 138,090 | q12 – q35.2 | 4506 | -0.46 | NA | *DCUN1D4, LRRC66, SGCB...* |
| chr4 | 69392576–69483277 | 91 | q13.2 | 4 | -2.63 | 9.17E–36 | *UGT2B17, UGT2B15* |
| chr4 | 108017707–114333471 | 6,316 | q25 – q26 | 233 | -0.56 | 1.37E–10 | *PAPSS1, SGMS2, CYP2U1...* |
| chr4 | 155104776–156950295 | 1,846 | q31.3 – q32.1 | 59 | -0.66 | 6.91E–11 | *DCHS2, PLRG1, FGB...* |
| chr5 | 57640–46365277 | 46,308 | p15.33 – p11 | 1643 | 0.43 | NA | *PLEKHG4B, LRRC14B, CCDC127...* |
| chr5 | 95243–4641468 | 4,546 | p15.33 – p15.32 | 226 | 0.31 | 1.91E–12 | *PLEKHG4B, LRRC14B, CCDC127...* |
| chr5 | 49638923–180684501 | 131,046 | q11.1 – q35.3 | 4413 | -0.48 | NA | *EMB, PARP8, ISL1...* |
| chr5 | 50483986–78674268 | 28,190 | q11.1 – q14.1 | 881 | -0.42 | 1.09E–12 | *ISL1, PELO, ITGA1...* |
| chr5 | 96482585–101310605 | 4,828 | q15 – q21.1 | 155 | -0.62 | 6.22E–13 | *RIOK2, RGMB, FLJ35946...* |
| chr5 | 101856676–109182598 | 7,326 | q21.1 – q21.3 | 236 | -0.38 | 7.18E–11 | *PAM, GIN1, PPIP5K2...* |
| chr5 | 154149241–166134854 | 11,986 | q33.2 – q34 | 387 | -0.61 | 1.95E–25 | *LARP1, C5orf4, CNOT8...* |
| chr7 | 54185–57842077 | 57,788 | p22.3 – p11.2 | 2071 | 0.69 | NA | *FAM20C, LOC100288524, LOC442497...* |
| chr7 | 3859349–5578181 | 1,719 | p22.2 – p22.1 | 60 | 0.49 | 1.99E–10 | *SDK1, FOXK1, KIAA0415...* |
| chr7 | 45953258–48774243 | 2,821 | p12.3 | 95 | 0.90 | 7.39E–18 | *IGFBP3, TNS3, C7orf65...* |
| chr7 | 49267969–50962948 | 1,695 | p12.2 – p12.1 | 93 | 0.42 | 1.45E–25 | *VWC2, ZPBP, C7orf72...* |
| chr7 | 54273841–55357325 | 1,083 | p11.2 | 37 | 0.96 | 7.02E–12 | *VSTM2A, SEC61G, EGFR* |
| chr7 | 121914744–122098748 | 184 | q31.32 | 33 | 0.42 | 6.09E–23 | *FEZF1, LOC154860, CADPS2* |
| chr8 | 15952011–16010296 | 58 | p22 | 3 | 1.02 | 2.81E–11 | *MSR1* |
| chr8 | 39258894–39381514 | 123 | p11.22 | 5 | 4.72 | NA | *ADAM5P, ADAM3A* |
| chr8 | 52127282–55854108 | 3,727 | q11.21 – q12.1 | 120 | 0.78 | NA | *PXDNL, PCMTD1, ST18...* |
| chr8 | 86847986–88292389 | 1,444 | q21.2 – q21.3 | 51 | 0.74 | NA | *PSKH2, ATP6V0D2, SLC7A13...* |
| chr9 | 115981–43469545 | 43,354 | p24.3 – p12 | 1536 | -0.50 | NA | *FOXD4, CBWD1, C9orf66...* |
| chr9 | 274517–3497979 | 3,223 | p24.3 – p24.2 | 175 | -0.61 | 8.38E–11 | *DOCK8, KANK1, DMRT1...* |
| chr9 | 10023842–10101447 | 78 | p23 | 4 | -1.04 | 2.27E–11 | *PTPRD* |
| chr9 | 71260258–141089296 | 69,829 | q21.11 – q34.3 | 2640 | 0.44 | NA | *PIP5K1B, FAM122A, PRKACG...* |
| chr9 | 80102875–82257893 | 2,155 | q21.2 – q21.31 | 81 | 0.61 | 1.87E–10 | *GNA14, GNAQ, CEP78...* |
| chr9 | 137039812–141008915 | 3,969 | q34.2 – q34.3 | 181 | 0.32 | 1.34E–10 | *RXRA, COL5A1, MIR3689A...* |
| chr10 | 45169698–45426457 | 257 | q11.21 | 11 | -0.52 | 4.56E–13 | *LOC220980, TMEM72* |
| chr10 | 61427922–62849169 | 1,421 | q21.2 | 49 | 0.26 | 2.46E–14 | *SLC16A9, CCDC6, C10orf40...* |
| chr11 | 210300–51538651 | 51,328 | p15.5 – p11.12 | 2197 | 0.38 | NA | *RIC8A, SIRT3, PSMD13...* |
| chr11 | 2016675–2016774 | 0 | p15.5 | 3 | 1.76 | 2.76E–18 | *H19* |
| chr11 | 10903031–14815348 | 3,912 | p15.3 – p15.2 | 127 | 0.57 | 3.98E–18 | *GALNTL4, CSNK2A1P, MIR4299...* |
| chr11 | 23437404–29474251 | 6,037 | p14.3 – p14.1 | 197 | 0.51 | 8.85E–13 | *LUZP2, ANO3, MUC15...* |
| chr11 | 55050707–103903754 | 48,853 | q11 – q22.3 | 1642 | 0.45 | NA | *OR4A16, OR4A15, OR4C15...* |
| chr11 | 55093530–92652140 | 37,559 | q11 – q14.3 | 1275 | 0.39 | 1.43E–23 | *OR4A16, OR4A15, OR4C15...* |
| chr11 | 95519128–103695043 | 8,176 | q21 – q22.3 | 268 | 0.76 | NA | *FAM76B, CEP57, MTMR2...* |
| chr12 | 9637323–9693948 | 57 | p13.31 | 3 | 4.21 | NA |  |
| chr13 | 49910490–115077940 | 65,167 | q14.2 – q34 | 2755 | 0.44 | NA | *CAB39L, SETDB2, PHF11…* |
| chr13 | 79553800–91458655 | 11,905 | q31.1 – q31.3 | 422 | 0.53 | 7.04E–15 | *RBM26, NDFIP2, SPRY2...* |
| chr13 | 91910086–101258636 | 9,349 | q31.3 – q32.3 | 569 | 0.34 | 1.75E–23 | *MIR17HG, MIR17, MIR18A...* |
| chr13 | 101587236–102442729 | 855 | q32.3 – q33.1 | 35 | 0.74 | 1.23E–13 | *NALCN, ITGBL1, FGF14* |
| chr14 | 106371690–106538480 | 167 | q32.33 | 5 | 3.54 | NA | *KIAA0125, ADAM6* |
| chr14 | 106803248–106957950 | 155 | q32.33 | 5 | 0.87 | 7.05E–16 | *NCRNA00221* |
| chr14 | 107148739–107182658 | 34 | q32.33 | 10 | -0.67 | 1.58E–18 |  |
| chr15 | 35455543–102399819 | 66,944 | q14 – q26.3 | 2692 | 0.38 | NA | *LOC723972, ATPBD4, MIR3942...* |
| chr15 | 35582852–37588674 | 2,006 | q14 | 75 | 0.56 | 1.84E–10 | *ATPBD4, MIR3942, C15orf41...* |
| chr16 | 96766–802416 | 706 | p13.3 | 105 | -0.47 | NA | *POLR3K, SNRNP25, RHBDF1...* |
| chr16 | 28861531–28929651 | 68 | p11.2 | 4 | 0.74 | 6.21E–10 | *SH2B1, ATP2A1, RABEP2* |
| chr16 | 34001104–35194100 | 1,193 | p11.2 – p11.1 | 69 | 0.29 | 4.99E–24 | *UBE2MP1, LOC283914, LOC146481...* |
| chr16 | 46441545–78344964 | 31,903 | q11.2 – q23.1 | 1223 | 0.48 | NA | *ANKRD26P1, SHCBP1, VPS35...* |
| chr16 | 46500741–65713545 | 19,213 | q11.2 – q21 | 773 | 0.37 | 4.99E–35 | *ANKRD26P1, SHCBP1, VPS35...* |
| chr16 | 68156107–77352056 | 9,196 | q22.1 – q23.1 | 300 | 0.74 | NA | *NFATC3, ESRP2, PLA2G15...* |
| chr16 | 78633183–78779049 | 146 | q23.1 | 7 | -1.36 | 4.01E–43 | *WWOX* |
| chr17 | 1693–3585764 | 3,584 | p13.3 – p13.2 | 259 | 0.41 | NA | *DOC2B, RPH3AL, C17orf97...* |
| chr17 | 7579695–7590367 | 11 | p13.1 | 7 | -0.58 | 1.32E–10 | *TP53, WRAP53* |
| chr17 | 25343175–32693506 | 7,350 | q11.1 – q12 | 356 | -0.47 | NA | *WSB1, LOC440419, KSR1...* |
| chr17 | 44254355–44351152 | 97 | q21.31 | 3 | 0.94 | 2.59E–10 | *KIAA1267, LOC644246* |
| chr18 | 35824979–78012829 | 42,188 | q12.2 – q23 | 1713 | -0.49 | NA | *LOC647946, KC6, PIK3C3...* |
| chr19 | 28057285–28419244 | 362 | q11 | 46 | -0.55 | NA | *LOC148189* |
| chr19 | 28431784–29178957 | 747 | q11 – q12 | 28 | 0.55 | 1.23E–33 |  |
| chr19 | 31448423–44284417 | 12,836 | q12 – q13.31 | 429 | -0.45 | NA | *DKFZp566F0947, TSHZ3, THEG5...* |
| chr20 | 67778–26312663 | 26,245 | p13 – p11.1 | 926 | 0.72 | NA | *DEFB125, DEFB126, DEFB127...* |
| chr20 | 7439090–9606616 | 2,168 | p12.3 – p12.2 | 71 | 0.53 | 1.51E–11 | *HAO1, TMX4, PLCB1...* |
| chr20 | 29462044–62949149 | 33,487 | q11.21 – q13.33 | 1288 | 0.68 | NA | *FRG1B, DEFB115, DEFB116...* |
| chr21 | 10701593–10944060 | 242 | p11.2 – p11.1 | 6 | -0.60 | 7.19E–10 | *TPTE* |
| chr21 | 14389029–48090317 | 33,701 | q11.2 – q22.3 | 1595 | -0.47 | NA | *ANKRD30BP2, MIR3156–3, POTED...* |
| chr21 | 36272934–37182165 | 909 | q22.12 | 103 | -0.29 | 1.02E–13 | *RUNX1, C21orf96, MIR802* |
| chr22 | 19747933–19749476 | 2 | q11.21 | 4 | -1.09 | 1.26E–19 | *TBX1* |
| chr22 | 23056562–23228483 | 172 | q11.22 | 7 | 3.79 | NA | *MIR650* |
| chr22 | 30579046–41294768 | 10,716 | q12.2 – q13.2 | 409 | -0.47 | NA | *LIF, OSM, GATSL3...* |

NA indicates expression not detectable.
