# Supplementary material for: Genomic Alteration in Head and Neck Squamous Cell Carcinoma (HNSCC) Cell Lines Inferred from Karyotyping, Molecular Cytogenetics, and Array Comparative Genomic Hybridization
Source: PLoS One. 2016 Aug 8;11(8):e0160901. doi: 10.1371/journal.pone.0160901 (PMC4976893; doi:10.1371/journal.pone.0160901)
Supplement: S4 Table — (DOCX) [file pone.0160901.s012.docx]

**S4 Table** Genome view of chromosome copy number variation (CNV) in HN4 cell line.

| chromosome | start–stop (bp) | size (kb) | cytoband | #probes | amp/del | p–value | annotations |
| --- | --- | --- | --- | --- | --- | --- | --- |
| chr1 | 115419373–115493167 | 74 | p13.2 | 3 | -1.53 | 4.32E–10 | *SYCP1* |
| chr1 | 145466663–249208332 | 103,742 | q21.1 – q44 | 2321 | 0.38 | NA | *POLR3GL, ANKRD34A, LIX1L...* |
| chr1 | 180746048–180858503 | 112 | q25.3 | 3 | -2.67 | 4.34E–15 | *XPR1* |
| chr3 | 62199–90458199 | 90,396 | p26.3 – p11.1 | 2012 | -0.42 | NA | *CHL1, CNTN6, CNTN4...* |
| chr3 | 60629805–61027287 | 397 | p14.2 | 11 | -1.70 | 4.14E–19 | *FHIT* |
| chr3 | 93724712–197861598 | 104,137 | q11.1 – q29 | 2248 | 0.39 | NA | *ARL13B, STX19, DHFRL1...* |
| chr4 | 72447–49083290 | 49,011 | p16.3 – p11 | 1008 | -0.40 | NA | *ZNF595, ZNF718, ZNF876P...* |
| chr4 | 52703247–190642600 | 137,939 | q12 – q35.2 | 2774 | -0.39 | NA | *DCUN1D4, LRRC66, SGCB...* |
| chr6 | 158677452–158812696 | 135 | q25.3 | 3 | -2.31 | 3.81E–12 | *TULP4* |
| chr7 | 65558–57417402 | 57,352 | p22.3 – p11.2 | 1306 | 0.67 | NA | *FAM20C, LOC100288524, LOC442497...* |
| chr8 | 39258894–39381514 | 123 | p11.22 | 5 | 4.56 | 7.04E–34 | *ADAM5P, ADAM3A* |
| chr8 | 52101905–55756576 | 3,655 | q11.21 – q12.1 | 79 | 0.73 | NA | *PXDNL, PCMTD1, ST18...* |
| chr8 | 86868329–88385497 | 1,517 | q21.2 – q21.3 | 34 | 0.75 | 2.51E–22 | *PSKH2, ATP6V0D2, SLC7A13...* |
| chr9 | 229198–40162142 | 39,933 | p24.3 – p13.1 | 927 | -0.44 | NA | *DOCK8, KANK1, DMRT1...* |
| chr9 | 68369294–140948378 | 72,579 | q13 – q34.3 | 1717 | 0.77 | NA | *LOC642236, LOC100132352, PGM5P2...* |
| chr9 | 131640726–132130630 | 490 | q34.11 | 11 | -0.27 | 3.79E–12 | *CCBL1, LRRC8A, PHYHD1...* |
| chr11 | 325838–51538651 | 51,213 | p15.5 – p11.12 | 1379 | 0.32 | NA | *B4GALNT4, PKP3, SIGIRR...* |
| chr11 | 55050707–134934196 | 79,883 | q11 – q25 | 1712 | 0.33 | NA | *OR4A16, OR4A15, OR4C15...* |
| chr12 | 361756–34827047 | 34,465 | p13.33 – p11.1 | 793 | 0.39 | NA | *SLC6A13, KDM5A, CCDC77...* |
| chr12 | 9637323–9713425 | 76 | p13.31 | 3 | 3.37 | 1.75E–28 |  |
| chr12 | 38029401–49054117 | 11,025 | q11 – q13.11 | 268 | -0.36 | 1.80E–37 | *ALG10B, CPNE8, KIF21A...* |
| chr14 | 106453638–106538480 | 85 | q32.33 | 3 | 4.05 | 1.70E–21 |  |
| chr15 | 20055137–32432126 | 12,377 | q11.1 – q13.3 | 548 | 0.27 | 7.32E–43 | *HERC2P3, GOLGA6L6, GOLGA8C...* |
| chr15 | 34656203–102465355 | 67,809 | q14 – q26.3 | 1710 | 0.63 | NA | *LPCAT4, GOLGA8A, MIR1233–1...* |
| chr17 | 25278114–32747839 | 7,470 | q11.1 – q12 | 198 | -0.42 | 4.51E–38 | *WSB1, LOC440419, KSR1...* |
| chr17 | 78008301–78111444 | 103 | q25.3 | 3 | -2.76 | 6.72E–16 | *TBC1D16, CCDC40, GAA...* |
| chr18 | 148963–15072794 | 14,924 | p11.32 – p11.21 | 352 | -0.46 | NA | *USP14, THOC1, COLEC12...* |
| chr18 | 18652010–77961779 | 59,310 | q11.1 – q23 | 1450 | 0.39 | NA | *ROCK1, GREB1L, ESCO1...* |
| chr19 | 31139484–44264906 | 13,125 | q12 – q13.31 | 265 | -0.42 | NA | *DKFZp566F0947, TSHZ3, THEG5...* |
| chr20 | 135931–25678197 | 25,542 | p13 – p11.1 | 578 | 0.47 | NA | *DEFB127, DEFB128, DEFB129...* |
| chr20 | 29483588–62880583 | 33,397 | q11.21 – q13.33 | 831 | 0.66 | NA | *FRG1B, DEFB115, DEFB116...* |
| chr21 | 15499847–48090317 | 32,590 | q11.2 – q22.3 | 981 | -0.37 | NA | *LIPI, RBM11, ABCC13...* |
| chr22 | 30579046–51193680 | 20,615 | q12.2 – q13.33 | 540 | -0.32 | NA | *LIF, OSM, GATSL3...* |

NA indicates expression not detectable.
