# Supplementary material for: Genomic Alteration in Head and Neck Squamous Cell Carcinoma (HNSCC) Cell Lines Inferred from Karyotyping, Molecular Cytogenetics, and Array Comparative Genomic Hybridization
Source: PLoS One. 2016 Aug 8;11(8):e0160901. doi: 10.1371/journal.pone.0160901 (PMC4976893; doi:10.1371/journal.pone.0160901)
Supplement: S5 Table — (DOCX) [file pone.0160901.s013.docx]

**S5 Table** Genome view of chromosome copy number variation (CNV) in HN12 cell line.

| chromosome | start–stop (bp) | size (kb) | cytoband | #probes | amp/del | p–value | annotations |
| --- | --- | --- | --- | --- | --- | --- | --- |
| chr1 | 1050011–45503258 | 44,453 | p36.33 – p34.1 | 1495 | -0.35 | NA | *C1orf159, MIR200B, MIR200A...* |
| chr1 | 11204969–11537283 | 332 | p36.22 | 12 | 0.26 | 2.85E–13 | *MTOR, ANGPTL7, UBIAD1* |
| chr1 | 72329328–72493839 | 165 | p31.1 | 8 | -0.95 | 1.86E–20 | *NEGR1* |
| chr1 | 85988179–101724358 | 15,736 | p22.3 – p21.2 | 529 | 0.67 | NA | *DDAH1, CYR61, ZNHIT6...* |
| chr1 | 86497961–88265607 | 1,768 | p22.3 | 57 | 0.33 | 3.26E–19 | *COL24A1, ODF2L, CLCA2...* |
| chr1 | 94517761–98775497 | 4,258 | p22.1 – p21.3 | 143 | 0.84 | 6.20E–13 | *ABCA4, ARHGAP29, ABCD3...* |
| chr1 | 101747810–121330906 | 19,583 | p21.2 – p11.2 | 708 | -0.50 | NA | *OLFM3, DNAJA1P5, COL11A1...* |
| chr1 | 145421747–160186485 | 14,765 | q21.1 – q23.2 | 480 | 0.37 | NA | *TXNIP, POLR3GL, ANKRD34A...* |
| chr1 | 158043247–160135187 | 2,092 | q23.1 – q23.2 | 71 | 0.61 | 4.78E–12 | *KIRREL, LOC646268, CD1D...* |
| chr1 | 198734946–248113238 | 49,378 | q32.1 – q44 | 1776 | 0.26 | NA | *MIR181B1, MIR181A1, NR5A2...* |
| chr1 | 246261013–246341018 | 80 | q44 | 4 | -1.62 | 2.34E–35 | *SMYD3* |
| chr2 | 40404940–40645883 | 241 | p22.1 | 10 | -0.62 | 7.74E–12 | *SLC8A1* |
| chr2 | 45168836–45169314 | 0 | p21 | 4 | 1.05 | 2.76E–13 | *SIX3* |
| chr2 | 45172007–45172394 | 0 | p21 | 7 | 0.96 | 1.22E–18 | *SIX3* |
| chr2 | 89141608–89258800 | 117 | p11.2 | 6 | 1.56 | 3.62E–35 |  |
| chr2 | 95529039–125904475 | 30,375 | q11.1 – q14.3 | 1050 | -0.46 | NA | *LOC442028, TEKT4, MAL...* |
| chr2 | 141735849–141882126 | 146 | q22.1 | 6 | -0.90 | 3.66E–16 | *LRP1B* |
| chr2 | 185591359–194235687 | 8,644 | q32.1 – q32.3 | 289 | 0.53 | NA | *ZNF804A, FSIP2, ZC3H15...* |
| chr2 | 198593008–243040276 | 44,447 | q33.1 – q37.3 | 1658 | -0.51 | NA | *BOLL, PLCL1, SATB2...* |
| chr2 | 205559838–205624937 | 65 | q33.3 | 4 | -1.60 | 4.91E–14 | *PARD3B* |
| chr3 | 62199–90282104 | 90,220 | p26.3 – p11.1 | 3249 | -0.53 | NA | *CHL1, CNTN6, CNTN4...* |
| chr3 | 62199–9808463 | 9,746 | p26.3 – p25.3 | 497 | -0.62 | 5.32E–14 | *CHL1, CNTN6, CNTN4...* |
| chr3 | 71506648–71724981 | 218 | p13 | 8 | -1.23 | 4.01E–12 | *FOXP1, MIR1284* |
| chr3 | 81413060–82635750 | 1,223 | p12.2 | 38 | 0.64 | NA | *GBE1* |
| chr3 | 93538467–197837049 | 104,299 | q11.1 – q29 | 3582 | 0.37 | NA | *PROS1, ARL13B, STX19...* |
| chr4 | 72447–49505025 | 49,433 | p16.3 – p11 | 1679 | -0.48 | NA | *ZNF595, ZNF718, ZNF876P...* |
| chr4 | 52701080–190874516 | 138,173 | q12 – q35.2 | 4499 | -0.46 | NA | *DCUN1D4, LRRC66, SGCB...* |
| chr4 | 69392576–69483277 | 91 | q13.2 | 4 | 0.56 | 1.19E–12 | *UGT2B17, UGT2B15* |
| chr4 | 91842748–91940897 | 98 | q22.1 | 4 | -1.53 | 1.17E–13 | *FAM190A* |
| chr4 | 92046537–104396298 | 12,350 | q22.1 – q24 | 393 | -0.37 | 5.96E–10 | *FAM190A, GRID2, ATOH1...* |
| chr4 | 143140695–143249560 | 109 | q31.21 | 4 | -1.60 | 4.56E–15 | *INPP4B* |
| chr4 | 152332224–153015219 | 683 | q31.3 | 24 | -1.03 | 1.31E–21 | *FAM160A1, PET112L* |
| chr5 | 22149–46365277 | 46,343 | p15.33 – p11 | 1645 | 0.99 | NA | *PLEKHG4B, LRRC14B, CCDC127...* |
| chr5 | 9053543–28542839 | 19,489 | p15.31 – p14.1 | 626 | 1.07 | 2.43E–10 | *SEMA5A, SNORD123, TAS2R1...* |
| chr5 | 39523077–46175247 | 6,652 | p13.1 – p11 | 251 | 0.80 | 7.72E–27 | *PTGER4, TTC33, PRKAA1...* |
| chr6 | 1814088–2155812 | 342 | p25.3 | 12 | -0.65 | 8.10E–15 | *GMDS* |
| chr6 | 56252017–56548108 | 296 | p12.1 | 20 | 0.75 | 5.56E–31 | *DST* |
| chr6 | 56560310–56634993 | 75 | p12.1 | 16 | -0.63 | 4.57E–18 |  |
| chr6 | 56677461–58774324 | 2,097 | p12.1 – p11.1 | 68 | 0.55 | NA | *BEND6, KIAA1586, ZNF451...* |
| chr6 | 70647871–71377089 | 729 | q13 | 27 | -0.53 | 1.23E–20 | *COL19A1, COL9A1, FAM135A...* |
| chr6 | 81898407–86053138 | 4,155 | q14.1 – q14.3 | 132 | 0.45 | NA | *FAM46A, IBTK, TPBG...* |
| chr6 | 86105898–87004746 | 899 | q14.3 | 31 | -0.42 | 5.16E–16 | *NT5E, SNX14, SYNCRIP...* |
| chr6 | 87044032–108146477 | 21,102 | q14.3 – q21 | 824 | -1.24 | NA | *HTR1E, CGA, ZNF292...* |
| chr6 | 87044032–87463988 | 420 | q14.3 | 13 | -0.74 | 2.80E–10 |  |
| chr6 | 108263433–120105607 | 11,842 | q21 – q22.31 | 381 | -0.35 | NA | *SEC63, OSTM1, NR2E1...* |
| chr7 | 54185–57262076 | 57,208 | p22.3 – p11.2 | 2056 | 0.73 | NA | *FAM20C, LOC100288524, LOC442497...* |
| chr7 | 54185–27578446 | 27,524 | p22.3 – p15.2 | 963 | 0.90 | NA | *FAM20C, LOC100288524, LOC442497...* |
| chr7 | 28206079–53007809 | 24,802 | p15.1 – p12.1 | 917 | 0.56 | NA | *JAZF1, LOC100128081, CREB5...* |
| chr7 | 37343082–39222347 | 1,879 | p14.1 | 63 | 0.34 | 8.22E–10 | *ELMO1, GPR141, TXNDC3...* |
| chr7 | 49190284–50981208 | 1,791 | p12.2 – p12.1 | 97 | 0.35 | 2.92E–13 | *VWC2, ZPBP, C7orf72...* |
| chr7 | 64440267–66709382 | 2,269 | q11.21 | 68 | 1.56 | NA | *ZNF117, ERV3, CCT6P3...* |
| chr7 | 66849415–69458876 | 2,609 | q11.21 – q11.22 | 88 | 0.49 | NA | *AUTS2* |
| chr7 | 125897363–126043670 | 146 | q31.33 | 8 | -0.62 | 7.92E–10 |  |
| chr7 | 128584322–131521885 | 2,938 | q32.1 – q32.3 | 149 | 0.32 | 5.66E–40 | *IRF5, TNPO3, TPI1P2...* |
| chr7 | 131564190–158148967 | 26,585 | q32.3 – q36.3 | 1152 | -0.59 | NA | *PLXNA4, CHCHD3, EXOC4...* |
| chr7 | 155250850–157723045 | 2,472 | q36.3 | 178 | -0.45 | 2.04E–11 | *EN2, CNPY1, RBM33...* |
| chr8 | 119720–43708292 | 43,589 | p23.3 – p11.1 | 1579 | -0.49 | NA | *RPL23AP53, ZNF596, FBXO25...* |
| chr8 | 47735940–63817073 | 16,081 | q11.1 – q12.3 | 599 | 0.74 | NA | *NCRNA00293, LOC100287846, KIAA0146...* |
| chr8 | 47735940–49354391 | 1,618 | q11.1 – q11.21 | 94 | 1.17 | 1.40E–45 | *NCRNA00293, LOC100287846, KIAA0146...* |
| chr8 | 55158316–62267807 | 7,109 | q11.23 – q12.3 | 267 | 0.62 | 1.24E–12 | *SOX17, RP1, XKR4...* |
| chr8 | 119072248–146138887 | 27,067 | q24.11 – q24.3 | 898 | 0.38 | NA | *EXT1, SAMD12, TNFRSF11B...* |
| chr9 | 204193–39140270 | 38,936 | p24.3 – p13.1 | 1508 | -0.43 | NA | *C9orf66, DOCK8, KANK1...* |
| chr9 | 9400269–9782886 | 383 | p23 | 15 | -1.24 | 1.09E–27 | *PTPRD* |
| chr9 | 20809914–20951944 | 142 | p21.3 | 7 | -1.48 | 1.23E–21 | *KIAA1797* |
| chr9 | 21993401–22036505 | 43 | p21.3 | 3 | -5.43 | NA | *CDKN2A, CDKN2B–AS1, CDKN2B* |
| chr9 | 66710577–68327774 | 1,617 | q13 | 13 | -0.57 | 1.62E–12 | *LOC100133920, AQP7P1, FAM27B...* |
| chr9 | 113692821–141089296 | 27,396 | q31.3 – q34.3 | 1076 | 0.41 | NA | *LPAR1, OR2K2, KIAA0368...* |
| chr10 | 102539–39047586 | 38,945 | p15.3 – p11.1 | 1473 | -0.34 | NA | *ZMYND11, DIP2C, C10orf108...* |
| chr10 | 53670917–59783010 | 6,112 | q21.1 | 208 | 0.34 | 9.18E–13 | *PRKG1, DKK1, MBL2...* |
| chr10 | 99970823–102300490 | 2,330 | q24.2 – q24.31 | 82 | 0.62 | 1.97E–39 | *C10orf28, LOXL4, PYROXD2...* |
| chr11 | 2016612–2017012 | 0 | p15.5 | 6 | 1.34 | 1.14E–25 | *H19* |
| chr11 | 2017725–2020975 | 3 | p15.5 | 16 | 0.57 | 6.38E–11 | *H19, MIR675* |
| chr11 | 55385617–55450788 | 65 | q11 | 3 | -1.76 | 3.53E–25 | *OR4P4, OR4S2, OR4C6* |
| chr11 | 62685219–67511266 | 4,826 | q12.3 – q13.2 | 182 | 0.29 | 2.80E–42 | *CHRM1, SLC22A6, SLC22A8...* |
| chr11 | 67793815–105046236 | 37,252 | q13.2 – q22.3 | 1217 | 0.84 | NA | *ALDH3B1, NDUFS8, TCIRG1...* |
| chr11 | 67828291–74615074 | 6,787 | q13.2 – q13.4 | 228 | 0.63 | 3.24E–28 | *CHKA, SUV420H1, C11orf24...* |
| chr11 | 74920948–77862989 | 2,942 | q13.4 – q14.1 | 98 | 1.09 | 1.89E–17 | *LOC441617, ARRB1, MIR326...* |
| chr11 | 121308802–134934196 | 13,625 | q24.1 – q25 | 479 | -0.90 | NA | *SORL1, MIR100HG, MIR125B1...* |
| chr12 | 9637323–9693948 | 57 | p13.31 | 3 | 2.99 | 7.36E–37 |  |
| chr13 | 19590141–21861250 | 2,271 | q12.11 | 126 | -1.19 | NA | *PHF2P1, TUBA3C, ANKRD26P3...* |
| chr13 | 51159942–51229962 | 70 | q14.3 | 4 | -4.20 | NA |  |
| chr13 | 63901067–115092648 | 51,192 | q21.31 – q34 | 2262 | 0.50 | NA | *OR7E156P, PCDH9, KLHL1...* |
| chr13 | 77024504–79510601 | 2,486 | q22.2 – q31.1 | 117 | 0.29 | 3.08E–15 | *KCTD12, BTF3P11, CLN5...* |
| chr14 | 19265142–20421677 | 1,157 | q11.2 | 18 | 0.86 | 5.65E–26 | *OR11H12, POTEG, POTEM...* |
| chr14 | 41616413–41657239 | 41 | q21.1 | 3 | -1.18 | 6.71E–14 |  |
| chr14 | 45565742–45604491 | 39 | q21.2 | 3 | -1.05 | 2.19E–11 | *PRPF39, SNORD127, FKBP3* |
| chr14 | 66747335–77659407 | 10,912 | q23.3 – q24.3 | 375 | 0.34 | 1.79E–39 | *NCRNA00238, GPHN, FAM71D...* |
| chr14 | 77888839–107287505 | 29,399 | q24.3 – q32.33 | 1148 | -0.43 | NA | *C14orf148, VIPAR, AHSA1...* |
| chr14 | 106371690–106538480 | 167 | q32.33 | 5 | 3.59 | NA | *KIAA0125, ADAM6* |
| chr14 | 106561123–106957950 | 397 | q32.33 | 12 | 0.37 | 2.44E–21 | *NCRNA00226, NCRNA00221* |
| chr14 | 106990277–107281934 | 292 | q32.33 | 40 | -0.79 | 5.28E–15 |  |
| chr15 | 22698522–59955586 | 37,257 | q11.2 – q22.2 | 1986 | -0.52 | NA | *GOLGA8DP, GOLGA6L1, TUBGCP5...* |
| chr15 | 44567172–44630003 | 63 | q15.3 | 4 | 0.39 | 1.91E–10 | *CASC4* |
| chr15 | 69721961–86038871 | 16,317 | q23 – q25.3 | 560 | 0.42 | NA | *KIF23, RPLP1, LOC145837...* |
| chr15 | 88677600–97538579 | 8,861 | q25.3 – q26.2 | 381 | -0.52 | NA | *NTRK3, MRPL46, MRPS11...* |
| chr16 | 96766–35194100 | 35,097 | p13.3 – p11.1 | 1482 | -0.35 | NA | *POLR3K, SNRNP25, RHBDF1...* |
| chr16 | 1634585–1804845 | 170 | p13.3 | 6 | -1.47 | 3.86E–27 | *IFT140, CRAMP1L, HN1L...* |
| chr16 | 34226241–35148939 | 923 | p11.2 – p11.1 | 63 | -0.98 | NA | *UBE2MP1, LOC283914, LOC146481...* |
| chr16 | 46467474–90102469 | 43,635 | q11.2 – q24.3 | 1675 | -0.53 | NA | *ANKRD26P1, SHCBP1, VPS35...* |
| chr16 | 78206297–78818301 | 612 | q23.1 | 25 | -1.76 | NA | *WWOX* |
| chr17 | 11529154–14822108 | 3,293 | p12 | 110 | 0.61 | NA | *DNAH9, ZNF18, MAP2K4...* |
| chr17 | 25300199–30323894 | 5,024 | q11.1 – q11.2 | 276 | 0.42 | NA | *WSB1, LOC440419, KSR1...* |
| chr17 | 52817574–78416073 | 25,599 | q22 – q25.3 | 886 | 0.42 | NA | *TOM1L1, COX11, STXBP4...* |
| chr17 | 53760598–63826547 | 10,066 | q22 – q24.1 | 342 | 0.36 | 1.52E–23 | *TMEM100, PCTP, ANKFN1...* |
| chr17 | 65356908–73108973 | 7,752 | q24.2 – q25.1 | 280 | 0.74 | 2.82E–36 | *PSMD12, PITPNC1, NOL11...* |
| chr18 | 148963–14947543 | 14,799 | p11.32 – p11.21 | 561 | -0.51 | NA | *USP14, THOC1, COLEC12...* |
| chr18 | 18569095–20801289 | 2,232 | q11.1 – q11.2 | 97 | -0.44 | NA | *ROCK1, GREB1L, ESCO1...* |
| chr18 | 27459126–28515161 | 1,056 | q12.1 | 35 | 1.34 | NA | *MIR302F* |
| chr18 | 28552510–78012829 | 49,460 | q12.1 – q23 | 1958 | -1.39 | NA | *DSC3, DSC2, DSC1...* |
| chr19 | 259395–8439376 | 8,180 | p13.3 – p13.2 | 280 | -0.46 | NA | *PPAP2C, MIER2, THEG...* |
| chr19 | 8476231–12111843 | 3,636 | p13.2 | 106 | 0.61 | NA | *MARCH2, HNRNPM, PRAM1...* |
| chr19 | 9040014–11053972 | 2,014 | p13.2 | 59 | 0.37 | 9.13E–11 | *MUC16, OR1M1, OR7G2...* |
| chr19 | 11140861–11817283 | 676 | p13.2 | 22 | 1.27 | 2.79E–26 | *SMARCA4, LDLR, SPC24...* |
| chr19 | 21631482–22926398 | 1,295 | p12 | 42 | -0.52 | 6.04E–31 | *LOC400680, ZNF429, ZNF100...* |
| chr19 | 27764285–53242518 | 25,478 | q11 – q13.41 | 865 | -0.48 | NA | *LOC148189, LOC148145, UQCRFS1...* |
| chr20 | 7439090–26225182 | 18,786 | p12.3 – p11.1 | 654 | -0.36 | NA | *HAO1, TMX4, PLCB1...* |
| chr20 | 56033192–59901663 | 3,868 | q13.31 – q13.33 | 195 | 0.47 | NA | *CTCFL, PCK1, ZBP1...* |
| chr21 | 36205879–36232084 | 26 | q22.12 | 4 | -1.23 | 1.94E–17 | *RUNX1* |
| chr21 | 47631649–47836390 | 205 | q22.3 | 17 | -0.48 | 8.42E–12 | *LSS, MCM3AP– AS1, MCM3AP...* |
| chr22 | 17280628–18191038 | 910 | q11.1 – q11.21 | 82 | 0.32 | 2.30E–23 | *XKR3, HSFY1P1, GAB4...* |
| chr22 | 18230460–23442209 | 5,212 | q11.21 – q11.22 | 254 | 0.90 | NA | *BID, MIR3198, MICAL3...* |
| chr22 | 23056562–23228483 | 172 | q11.22 | 7 | 4.66 | NA | *MIR650* |
| chr22 | 23533447–28675052 | 5,142 | q11.23 – q12.1 | 210 | 0.37 | NA | *BCR, FBXW4P1, CES5AP1...* |
| chr22 | 28701765–29560371 | 859 | q12.1 | 37 | -0.46 | 1.16E–21 | *TTC28, CHEK2, HSCB...* |
| chr22 | 29622990–30397599 | 775 | q12.2 | 58 | 0.29 | 1.19E–14 | *EMID1, RHBDD3, EWSR1...* |
| chr22 | 30413840–30647965 | 234 | q12.2 | 11 | 1.03 | 9.81E–32 | *MTMR3, HORMAD2, LIF* |
| chr22 | 30672442–51177360 | 20,505 | q12.2 – q13.33 | 851 | -0.41 | NA | *GATSL3, TBC1D10A, SF3A1...* |

NA indicates expression not detectable.
